# Genetically predicted lifestyle factors, socioeconomic status and risk of coronary artery disease in individuals with diabetes: a Mendelian randomization study

**DOI:** 10.3389/fpubh.2023.1284958

**Published:** 2023-12-22

**Authors:** Zhenhua Mai, Shuang Wang, Hao Chen, Jingjing Zhang, Hao Liu, Le Zhao, Yongze Chen, Ruixian Huang, Hao Zhou, Xiaoming Chen, Yuanlin Ding, Danli Kong

**Affiliations:** ^1^Department of Critical Care Medicine, Affiliated Hospital of Guangdong Medical University, Zhanjiang, China; ^2^Department of Epidemiology and Medical Statistics School of Public Health, Guangdong Medical University, Dongguan, China; ^3^Department of Gastroenterology, Affiliated Hospital of Guangdong Medical University, Zhanjiang, China; ^4^Department of Hospital Infection Management of Nanfang Hospital, Southern Medical University, Guangzhou, China; ^5^Department of Endocrinology, Affiliated Hospital of Guangdong Medical University, Zhanjiang, China

**Keywords:** lifestyles, socioeconomic status, coronary artery disease, diabetes, Mendelian randomization

## Abstract

**Background:**

This study explores the causal links between genetically predicted lifestyle factors, socioeconomic status, and coronary artery disease (CAD) risk in individuals with diabetes using a bidirectional Mendelian-randomization approach.

**Methods:**

This study explored the potential causal relationships of lifestyle factors and socioeconomic status with the risk of CAD in diabetes patients by a bidirectional, two-sample Mendelian-randomization (MR) analysis.

**Results:**

Genetically predicted smoking initiation (*p* = 0.005, 95% *CI*: 1.08–1.55) and insomnia (*p* = 0.001, 95% *CI*: 1.06–1.29) were associated with a higher risk of CAD in individuals with diabetes, whereas educational attainment (*p* = 0.0001, 95% *CI*: 0.47–0.78) was associated with a lower risk of CAD. The lifetime smoking index (*p* = 0.016, 95% *CI*: 1.12–3.03) was suggestively associated with a higher risk of CAD, while household income before taxes (*p* = 0.048, 95% *CI*: 0.41–1.00) was suggestively associated with a lower risk of CAD. In addition, we observed a suggestive negative association between the genetically predicted risk of CAD and the lifetime smoking index (*p* = 0.016, 95% CI: 0.98–0.99) and a significant causal relationship between the risk of CAD and household income before taxes (*p* = 0.006, 95% *CI*: 0.97–0.99).

**Conclusion:**

The results of this study provide evidence that smoking initiation, lifetime smoking index and insomnia are associated with an increased risk of CAD in individuals with diabetes, educational attainment and household income before taxes are associated with a reduced risk of CAD in individuals with diabetes, and the possible role of lifetime smoking index and household income before taxes on the risk of CAD in individuals with diabetes. It provides an opportunity for the prevention and management of CAD in individuals with diabetes.

## Introduction

Among the top causes of mortality worldwide, cardiovascular disease is one of the most prevalent. The Global Burden of Cardiovascular Disease report reveals that 18.6 million people died from this condition in 2019, while the number of cases worldwide reached 523 million (1). Coronary artery disease (CAD) has the highest incidence among all types of cardiovascular disease and is widely prevalent across the globe. Diabetes mellitus (DM) is a significant risk factor for coronary heart disease (CHD). Diabetic individuals are two to three times more prone to develop CHD than nondiabetic individuals (2), and the prevalence of obstructive CAD is approximately 25% in diabetic individuals, which is substantially higher than that in nondiabetic individuals (3). In 2021, there were 2.30 million cardiovascular deaths. Additionally, 5.4 million deaths from elevated fasting plasma glucose (FPG) occur overall (4). Therefore, identifying potential risk factors for CAD in people with diabetes and taking timely interventions to reduce the occurrence of coronary events are of utmost importance.

Observational studies conducted previously have strongly linked lifestyle factors and socioeconomic status to the likelihood of developing CAD in people with diabetes (5–10). A prospective study reported that, in comparison to smokers with higher education levels, those with lower education had an increased risk of developing cardiovascular disease of 1.5% in women and 3.1% in men (8). Wang et al. reported that longer or shorter sleep was significantly linked to an increased risk of CHD. A one-hour reduction in sleep duration increased the risk of CHD by 11%, while a one-hour increase in sleep duration was associated with a 7% increase in the risk of CHD compared with 7 h of sleep per day (11). According to a cross-sectional study, health-related quality of life factors such as personal income level and exercise were found to have a negative association with the risk of stable angina, which is one of the most prevalent clinical types of CAD (12).

Nevertheless, observational studies are vulnerable to potential confounders, as well as reverse causation, which can lead to biased associations. For example, the association of alcohol consumption with cardiometabolic health-related diseases has been controversial for decades in epidemiological studies. Some observational studies exploring the association between alcohol consumption and cardiovascular disease have shown that moderate alcohol consumption is linked to a decreased risk of CAD (10, 13). In addition, for exposure factors that are detrimental to human health, such as smoking, it is challenging to explore causal associations through experimental design, and other clinical research methods are needed.

Mendelian randomization (MR) is an innovative method that employs genetic variation as an auxiliary variable to evaluate the causal relationship between risk factors and outcomes (14). MR uses the genetic nature of genes to model random assignment, using genes as a tool to explore whether the impact of a factor on a particular disease is indeed causal. Genetic variation is independent of other influences, so MR can effectively avoid bias caused by confounding factors and is less prone to reverse causality, which is common in observational studies (15–17). Utilizing a bidirectional two-sample MR study, we aimed to explore the causal link between lifestyle factors, socioeconomic status, and the risk of CAD in individuals with diabetes.

## Materials and methods

### Study design

**Figure 1:**
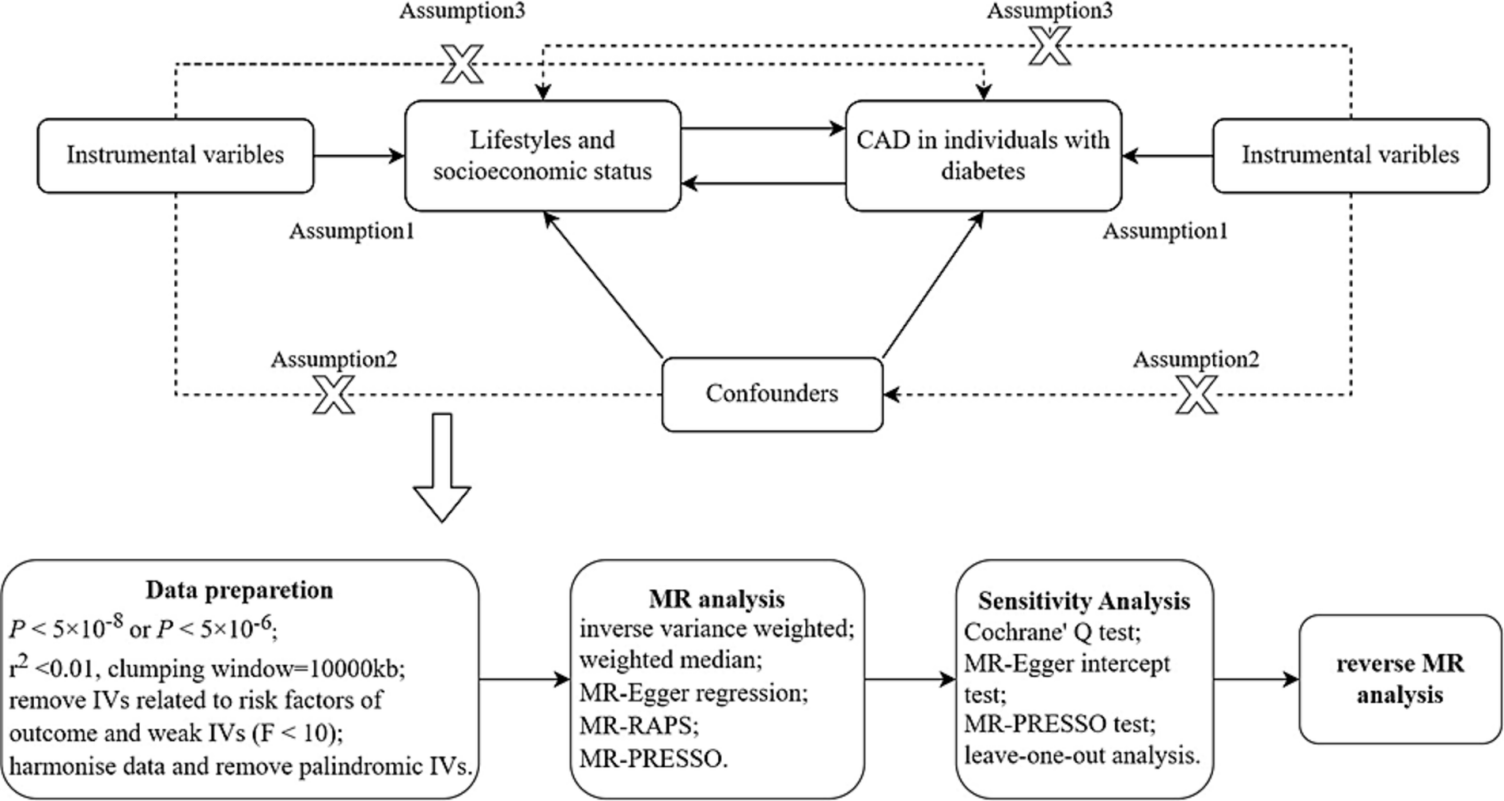
Diagram of MR framework in this study. CAD, coronary artery disease; IV, instrumental variable. Assumption 1 is that IVs must be strongly associated with exposure; assumption 2 is that IVs are not associated with any confounders in the exposure-outcome association; assumption 3 is that IVs should not be directly associated with outcome.

This is a bidirectional MR study. MR analysis uses genetic variants as instrumental variables (IVs) (18), and three key assumptions must be satisfied. The complete study design and the specific contents of the three assumptions are shown in Figure 1. This study was designed to determine the association between lifestyle factors, socioeconomic status and CAD in individuals with diabetes. First, we examined the causal relationship between lifestyle factors, socioeconomic status and CAD in individuals with diabetes. In addition, a reverse MR analysis was performed to examine the causal association between CAD in individuals with diabetes and lifestyle factors and socioeconomic status. Our study used abstract-level statistics only and thus did not involve ethical approval.

### Genome-wide association study summary data source

Single-nucleotide polymorphisms (SNPs) associated with seven lifestyle factors (including smoking initiation, lifetime smoking index, alcohol consumption, sleep duration, insomnia, physical activity, and coffee consumption) and three measures of socioeconomic status (including educational attainment, household income, and Townsend deprivation index) were obtained from genome-wide association studies (GWASs) corresponding to phenotypes retrieved from PubMed and MRC-IEU (19–24). Each phenotype is defined and described in detail in Supplementary Table S1.

Abstract-level data for the associations of lifestyle- and socioeconomic-associated SNPs with CAD in individuals with diabetes were extracted from a GWAS included in UK Biobank (25), including 15,666 individuals of European ancestry with diabetes (3,968 CAD patients and 11,698 control individuals). Diabetes status was defined according to an algorithm (26), screened from baseline UK biobank participants based on self-reported disease, medication, and age at the onset of diabetes. The criteria for CAD inclusion were CAD based on the UK Biobanks baseline assessment of verbal health interviews, combined with relevant data from hospital admission and death registries.

### Instrumental variables

We extracted SNPs associated with seven lifestyle factors, three measures of socioeconomic status, and CAD in individuals with diabetes, *p* < 5.0 × 10^−8^ (*p* < 5 × 10^−6^ in CAD in individuals with diabetes), ensuring associations between SNPs and each phenotype. After removal of the linkage disequilibrium (LD) (27), independent SNPs (if *r^2^* < 0.01 and clumping window > 10,000 kb, there is no LD in SNPs) were used as IVs. Physical activity was defined using average accelerometer-based physical activity (hereafter referred to as AMPA). To avoid pleiotropic effects, SNPs associated with multiple phenotypes were excluded. Moreover, we searched for the remaining SNPs in PhenoScanner, excluding those associated with other traits of genome-wide significance (28). Finally, the F-statistic was calculated to quantify the strength of the selected SNPs. An F-statistic greater than 10 suggested a strong correlation between the IVs and exposure factors. Matching SNPs were queried in the outcome GWAS, and if an SNP of the instrument was not available in the outcome GWAS, the SNP was assessed for the presence of a “proxy” SNP in the linkage imbalance at *r*^2^ > 0.8. Furthermore, the effect of the SNP on exposure should correspond to the same allele as the effect on the outcome, and palindromic SNPs are excluded.

### Statistical analysis

The inverse-variance weighted (IVW) method (29) was used as the main statistical analysis method. It is worth noting that fixed or random-effects IVW was selected based on the *p* value of the subsequent heterogeneity test (30). Weighted median (31), MR–Egger regression (32), MR-robust adjusted profile score (MR-RAPS) (33) and MR-pleiotropy residual sum and outlier (MR-PRESSO) (34) were used as four complementary analysis methods to check the consistency of the associations. IVW is primarily used for basic causal estimation, which will provide the most accurate results when all selected SNPs are valid IVs. The weighted median is obtained by first calculating the Wald ratio causal estimate for each SNP and then taking the estimate with the median inverse-variance weight. MR–Egger is an extension of IVW, which relaxes the assumption that any pleiotropy must be balanced. The significant intercept term indicates a bias in directivity pleiotropy, that is, the mean pleiotropy effect is not zero. MR-RAPS is an extension of IVW into a generic framework that allows for many weak tools. Exposure and outcome SNP effect estimates do not require sample overlap. MR-PRESSO is used to compare the actual distance and expected distance between genetic variation and regression line without horizontal pleiotropy and evaluate the causal estimate after removing the outlier.

To find the heterogeneity of SNP estimates in each MR association, we adopted the IVW method and MR–Egger regression, which quantified the heterogeneity by Cochran’s Q statistic. When the *p-*value was less than 0.05, there was heterogeneity. The *p-*value of the intercept test from MR–Egger regression was used to evaluate horizontal pleiotropy (32). In addition, MR-PRESSO was performed to assess the presence of pleiotropy by removing outliers and determining whether there was a material change in the causal effect before and after the removal of outliers (34). Finally, the robustness of the results was checked by leave-one-out analysis.

Causality was assessed as the odds ratio (*OR*) between exposure and outcome, as well as its 95% *CI* and *p-*value. A two-sided *p* < 0.05 was considered indicative of statistical significance. We used Bonferroni correction to further adjust the threshold for the number of phenotypes exposed (35). Therefore, for 10 exposures, the threshold of statistical significance was set at *p* < 0.05/10 = 0.005. When the *p* value was greater than 0.005 but less than 0.05, it was considered a suggestive association.

All analyses in this study were performed using the “TwoSampleMR,” “MendelianRandomization” and “MRPRESSO” packages in R version 4.2.2.

## Results

### Forwards MR analysis

A total of 286, 136, 76, 46, 198, 5, 10, 372, 48, and 17 independent SNPs were selected as instruments of smoking initiation, lifetime smoking index, alcohol consumption, sleep duration, insomnia, AMPA, coffee consumption, educational attainment, household income before tax, and Townsend deprivation index with respect to CAD in individuals with diabetes, respectively. Supplementary Tables S2–S11 list all SNPs selected above along with the F-statistics.

**Figure 2:**
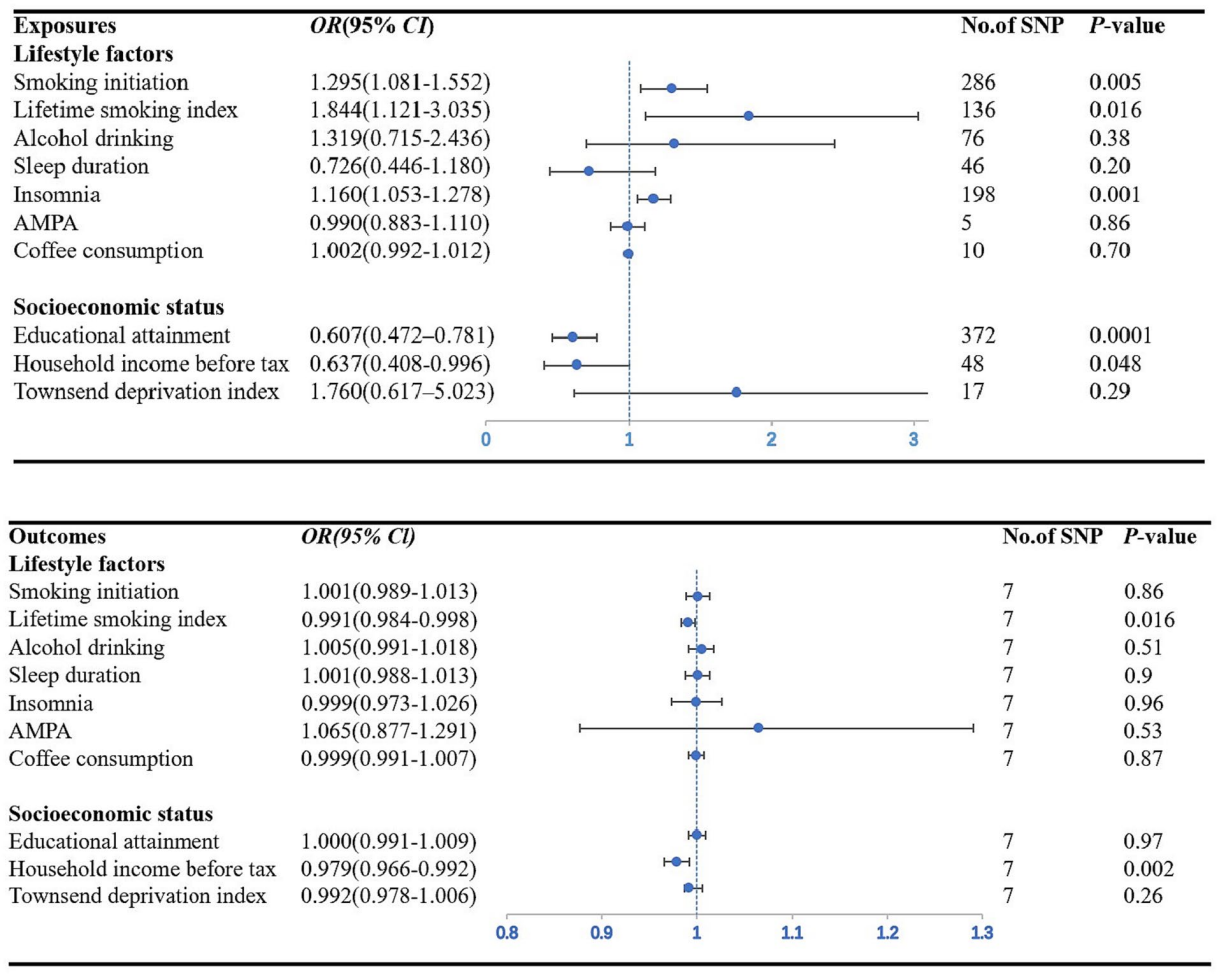
Forest plot showing IVW results from the MR study to evaluate potential causal associations between exposures and the risk of CAD in individuals with diabetes. AMPA accelerometer-based physical activity.

We found a significant causal relationship between smoking initiation, insomnia and CAD in individuals with diabetes. IVW analysis demonstrated that genetically predicted smoking initiation (*OR*: 1.30; *95% CI*: 1.08–1.55; *p* = 0.005) and insomnia (*OR*: 1.17; *95% CI*: 1.06–1.29; *p* = 0.001) were associated with an increased risk of CAD in individuals with diabetes. Similar associations were also observed in the MR-RAPS and MR-PRESSO methods (Supplementary Table S13). Based on Cochran’s *Q*-test, the results showed no significant heterogeneity (smoking initiation: *p* = 0.77; insomnia: *p* = 0.73). There is no evidence of horizontal pleiotropic bias by using MR–Egger regression (*P* for intercept = 0.39 for smoking initiation; *P* for intercept = 0.40 for insomnia) and MR-PRESSO global test (*p* = 0.77; *p* = 0.74) (Table 1). The leave-one-out analysis showed that the estimated effects detected did not depend on the specific SNP (Supplementary Figure S1). In addition, we observed a suggestive positive association between the genetically predicted lifetime smoking index and the risk of CAD in individuals with diabetes (*OR*: 1.84; *95% CI*: 1.12–3.03; *p* = 0.016) (Figure 2). MR–Egger regression and MR-PRESSO global test did not identify horizontal pleiotropy between IVs and outcomes (*p* = 0.30; *p* = 0.09), and Cochran’s Q test did not detect heterogeneity (Q = 157.50, *p* = 0.09) (Table 1). No association between alcohol consumption, sleep duration, AMPA, or coffee consumption and the risk of CAD in individuals with diabetes was found using any method (Table 1; Supplementary Table S13).

**Table 1:** Results of Mendelian randomized sensitivity analysis of exposure to outcome.

Exposures	MR-Egger		IVW		MR-Egger intercept		MR-PRESSO
	Q-statistic	*P*-value	Q-statistic	*P*-value	Intercept	*P*-value	*P*-value
Lifestyle factors							
Smoking initiation	266.49	0.76	267.24	0.77	0.0065	0.39	0.77
Lifetime smoking index	156.25	0.09	157.50	0.09	0.0118	0.30	0.09
Alcohol drinking	79.98	0.30	80.00	0.33	0.0012	0.91	0.33
Sleep duration	57.86	0.08	59.52	0.07	−0.0166	0.27	0.07
Insomnia	183.53	0.73	184.24	0.73	0.0071	0.40	0.74
AMPA	1.16	0.76	1.25	0.87	0.0184	0.79	0.87
Coffee consumption	5.00	0.76	5.15	0.82	0.0083	0.71	0.85
Socioeconomic statuses							
Educational attainment	369.07	0.50	369.16	0.52	0.0017	0.76	0.52
Average total household income before tax	44.06	0.55	44.38	0.58	−0.0113	0.57	0.60
Townsend deprivation index	12.57	0.64	14.98	0.53	−0.0671	0.14	0.54

AMPA, accelerometer-based physical activity.

Figure 2 plots the forest plot of the association between three socioeconomic status-related phenotypes and CAD in individuals with diabetes. In the primary analysis, genetic susceptibility to educational attainment was negatively associated with the risk of CAD in individuals with diabetes [*OR* (*95% CI*): 0.61 (0.47–0.78); *p* = 0.0001]. The MR-RAPS and MR-PRESSO results were consistent with the IVW method, and the results of the other MR methods showed a consistent but nonsignificant direction (Supplementary Table S13). To assess the robustness of the results, a set of sensitivity analyses was conducted, including Cochran’s Q test (*Q* = 369.16, *p* = 0.52), MR–Egger intercept (*p* = 0.76) and MR-PRESSO global test (*p* = 0.52) (Table 1). The leave-one-out analysis revealed that no SNP drove the results, indicating that none of the estimates were violated (Supplementary Figure S1). Furthermore, we observed nominal associations between genetically predicted average total household income before taxes and lower odds of the risk of CAD in individuals with diabetes (*OR*: 0.64; *95% CI*: 0.41–1.00; *p* = 0.048; Cochrane’s Q *p* = 0.58) (Figure 2). The results of MR–Egger regression did not suggest evidence of directional pleiotropy (*p* = 0.57), and the MR-PRESSO global test also showed no horizontal pleiotropy (*p* = 0.60) (Table 1). Nevertheless, there was no evidence of a causal relationship between the Townsend deprivation index and the risk of CAD in individuals with diabetes by IVW (*OR*: 1.76; *95% CI*: 0.62–5.02; *p* = 0.29) (Figure 2).

### Reverse MR analysis

**Figure 3:**
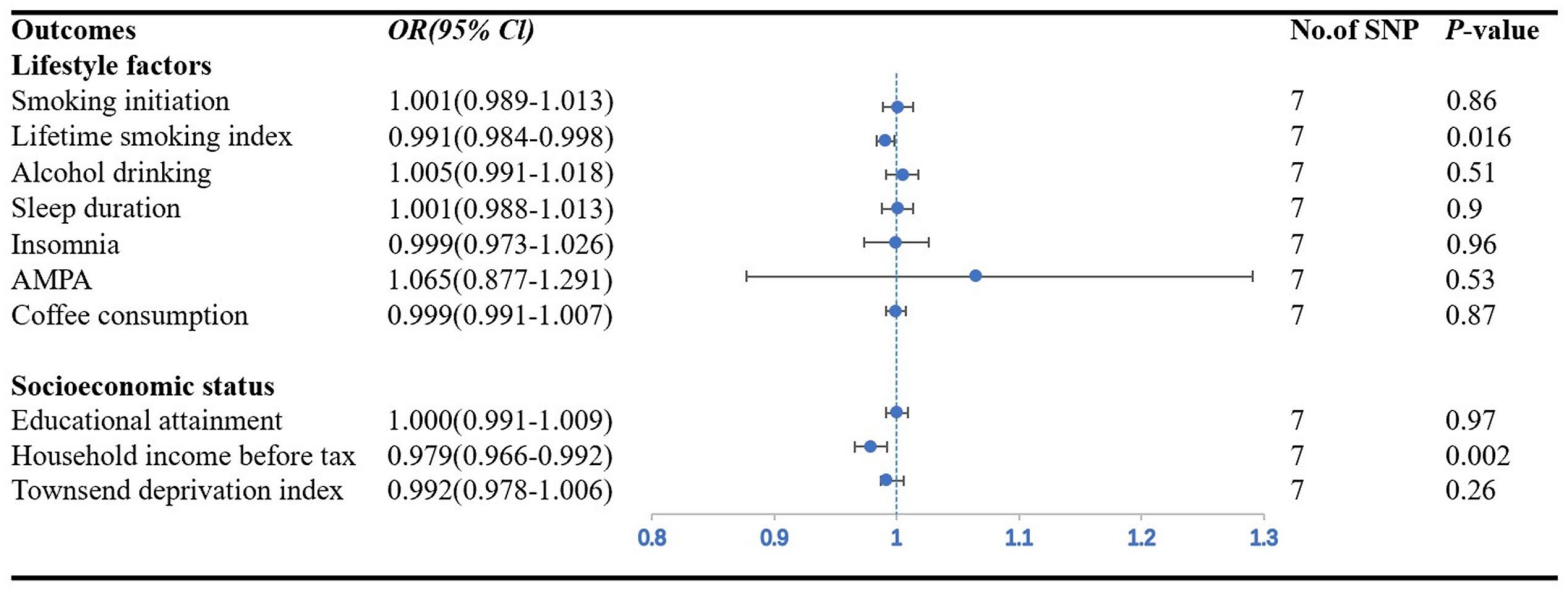
Forest plot showing IVW results from the MR study to evaluate potential causal associations between the risk of CAD in individuals with diabetes and outcomes. AMPA accelerometer-based physical activity.

We included 7 SNPs associated with CAD in individuals with diabetes, and all could be used to explore the association of the risk of CAD in individuals with diabetes with seven lifestyle factors and three measures of socioeconomic status. Summary information on the genetic instruments is shown in Supplementary Table S12. Figure 3 shows the forest diagram with the IVW method as the primary result.

Among the seven lifestyle factors, we observed a suggestive negative association between genetically predicted risk of CAD in individuals with diabetes and lifetime smoking index (*OR*: 0.99; *95% CI*: 0.98–0.99; *p* = 0.016) (Figure 3). MR–Egger regression and MR-PRESSO global test did not identify horizontal pleiotropy between IVs and outcomes (*p* = 0.50; *p* = 0.88), and Cochran’s Q test did not detect heterogeneity (*Q* = 2.59, *p* = 0.86) (Table 2).

**Table 2:** Results of Mendelian randomized sensitivity analysis of outcome to exposure.

Outcomes	MR-Egger		IVW		MR-Egger intercept		MR-PRESSO
	Q-statistic	*P*-value	Q-statistic	*P*-value	Intercept	*P*-value	*P*-value
Lifestyle factors							
Smoking initiation	9.37	0.10	9.43	0.15	−0.0007	0.86	0.15
Lifetime smoking index	2.07	0.84	2.59	0.86	−0.0015	0.50	0.88
Alcohol drinking	9.14	0.10	10.70	0.10	−0.0037	0.40	0.14
Sleep duration	0.57	0.98	0.58	0.99	0.0003	0.94	0.99
Insomnia	2.07	0.84	2.08	0.91	0.0006	0.94	0.92
AMPA	5.47	0.36	6.48	0.37	0.0532	0.38	0.41
Coffee consumption	4.44	0.49	4.59	0.60	0.0009	0.72	0.59
Socioeconomic statuses							
Educational attainment	5.70	0.34	5.76	0.45	0.0006	0.83	0.49
Average total household Income before tax	5.17	0.40	5.38	0.50	0.0017	0.68	0.56
Townsend deprivation index	10.51	0.06	11.24	0.08	−0.0025	0.58	0.10

AMPA, accelerometer-based physical activity.

In the three measures of socioeconomic status, we found a significant causal relationship between the risk of CAD in individuals with diabetes and household income before taxes (*OR*: 0.98; *95% CI*: 0.97–0.99; *p* = 0.006). The IVW method provided the primary results (Figure 3). MR–Egger regression and MR-PRESSO global test did not identify horizontal pleiotropy between IVs and outcomes (*p* = 0.68; *p* = 0.56), and Cochran’s Q test did not detect heterogeneity (*Q* = 5.38, *p* = 0.05) (Table 2).

## Discussion

This study employed a genetic approach to explore the potential bidirectional causal association between lifestyle factors, socioeconomic status, and the risk of CAD in patients with diabetes. Based on our research findings, we identified smoking initiation and insomnia as pathogenic risk factors for CAD in individuals with diabetes, while educational attainment was found to be a protective factor. Furthermore, our study did not find evidence supporting a reverse potential causal relationship, indicating that the presence of CAD in individuals with diabetes is likely to affect smoking initiation, insomnia, or educational attainment. Additionally, we observed a potential bidirectional association between the risk of CAD in individuals with diabetes and the lifetime smoking index as well as household income before taxes.

### Lifestyle factors

Smoking is a firmly established factor that increases the risk of developing cardiovascular disease (36), and our study confirms this association in diabetic patients. Specifically, our results revealed a 30% elevated risk of CAD in individuals with diabetes who initiated smoking, which supports previous traditional noninterventional studies. As an illustration, a meta-analysis encompassing 89 cohort studies (including 1.13 million people with diabetes) found that smokers had a 51% increased risk of CAD compared to nonsmokers (95% *CI*: 1.41–1.62) (37). Furthermore, Chen and colleagues reported a positive association between smoking initiation and CAD among patients with diabetes using MR analysis (*OR* = 1.32, 95% *CI*: 1.11–1.57), providing possible causal evidence to support clinical interventions for smoking cessation in this population (38). Furthermore, our study revealed a potential negative association between the lifetime smoking index and CAD in individuals with diabetes. According to our findings, CAD in individuals with diabetes has a potentially lower lifetime smoking index. These findings aimed to highlight the importance of targeted interventions to reduce smoking behavior to reduce the incidence of CAD in individuals with diabetes.

Sleep disturbance is a prevalent symptom in patients with diabetes (39, 40), and consistent with previous observational studies, our study revealed a 17% elevated risk of CAD among individuals with diabetes with insomnia. A meta-analysis of 13 prospective studies totaling 120,000 participants additionally demonstrated a 45% increased risk of cardiovascular disease incidence and/or mortality during the follow-up period among individuals with insomnia (*OR* = 1.45, 95% *CI*: 1.29–1.62) (41). However, our MR analysis did not provide evidence of a potential causal association between genetically estimated sleep duration and CAD in individuals with diabetes (*p* = 0.2). This could be due to the influence of potential confounding variables or nonbiological factors (mental health, stress and emotional status, etc.), which would suggest that the relationship is not causal. As such, our findings underscore the necessity of further exploration to gain a better understanding of the association between sleep disturbances and CAD in individuals with diabetes, taking into account the potential impact of nonbiological factors that may affect sleep duration and quality.

The potential impact of alcohol consumption on the risk of CAD in individuals with diabetes has been a topic of debate (10). While some studies have suggested a favorable effect of moderate alcohol consumption (42), our study failed to identify any potential causal relationship between alcohol consumption and the risk of CAD in individuals with diabetes (*p* = 0.38). It is possible that confounding factors may have influenced the results, such as metabolic abnormalities, hypertension, or high cholesterol, which can affect the health status of diabetic patients. Although we employed randomization to minimize the impact of confounding variables, their potential effects cannot be completely ruled out in this study. Additional investigations are required to gain a more comprehensive understanding of the association between alcohol consumption and the risk of CAD in individuals with diabetes.

### Socioeconomic statuses

Socioeconomic status is an important factor affecting the risk of CAD in individuals with diabetes (9). In agreement with prior observational studies, our MR study revealed a causative relationship between genetically inferred educational attainment and CAD risk in persons with diabetes. Specifically, diabetic patients with higher education had a significantly lower risk of CAD, with a reduction of 39%. These findings are in agreement with a recent MR study using genetic data from UK Biobank, which demonstrated that longer educational attainment is potentially causally associated with lower odds of cardiometabolic diseases, including DM and CAD (43). Our study highlights the significance of improving education to mitigate health inequalities and ameliorate cardiovascular health outcomes in individuals with diabetes.

Numerous studies have highlighted the higher prevalence of diabetes in low-income populations and the greater burden of cardiovascular risk factors among low-income diabetic patients (44, 45). Our study uncovers a potential bidirectional causal relationship between pretax household income and CAD among individuals with diabetes. Specifically, a genetically predicted higher pretax average household income is associated with a noteworthy 36% decrease in CAD risk. Conversely, the presence of diabetes itself has the potential to adversely impact pretax household income, thereby increasing the risk of CAD. The findings highlight the intricate interplay between socioeconomic factors, health conditions, and CAD risk in individuals with diabetes. These results shed light on the complex dynamics between income, diabetes, and CAD, emphasizing the importance of considering both genetic and environmental factors in understanding disease risk.

These results have important implications for improving public health measures and clinical practice, suggesting that prevention of CAD in individuals with diabetes could focus on lifestyle factors, including smoking and insomnia, and provide recommendations for smoking cessation and insomnia treatment. In clinical practice, health care professionals should comprehensively assess the patients’ smoking history, sleep quality and socioeconomic status and incorporate these factors into an individualized treatment plan. These efforts can help to reduce the risk of CAD in individuals with diabetes, improve patients’ quality of life, and reduce the burden on the health system.

### Strengths and limitations

There are several notable strengths to this study. First, we employed a two-sample MR analysis using genetically determined IVs to investigate the potential causal relationships of five lifestyle factors and three measures of socioeconomic status with CAD in individuals with diabetes. This approach effectively reduced the confounding and reverse causation that are prevalent in observational studies. Second, to enhance the validity and robustness of the study, two sets of instruments were used for smoking (smoking initiation and lifetime smoking index) and sleep behaviors (sleep duration and insomnia) for validation purposes. This strategy improved the accuracy and reliability of the results, reduced measurement errors, and effectively controlled for confounding factors to provide more precise and trustworthy conclusions.

There are certain limitations to be acknowledged when interpreting the results. First, the study consisted exclusively of individuals of European descent, and thus, the generalizability of our results to other populations may be limited. In addition, the selection of exposure factors in this paper is limited by the data sources and does not explore these complex exposure factors as deeply and comprehensively as possible. Second, the exposure factors were self-reported and subject to reporting bias, which may have affected the accuracy of our estimates. Finally, although MR analysis provides evidence of causality, reliability is still affected by factors such as sample size and external validity. It is important to acknowledge that large-sample multicenter randomized controlled trials remain the gold standard for establishing causal relationships.

In conclusion, our findings highlight the importance of addressing smoking initiation and insomnia as modifiable risk factors for CAD in individuals with diabetes while promoting educational attainment as a protective factor. Meanwhile, we found a potential causal effect of CAD in individuals with diabetes on the lifetime smoking index and household income before taxes. These results can have significant implications for clinical practice and public health policies aimed at reducing the burden of CAD in this population.

## Data availability statement

The original contributions presented in the study are included in the article/Supplementary material, further inquiries can be directed to the corresponding author.

## Author contributions

ZM: Writing – original draft, Writing – review & editing. SW: Writing – original draft, Writing – review & editing. HC: Writing – original draft, Writing – review & editing. JZ: Writing – original draft. HL: Writing – original draft. LZ: Writing – original draft. YC: Writing – original draft. RH: Writing – original draft. HZ: Writing – review & editing. XC: Writing – review & editing. YD: Writing – review & editing. DK: Writing – review & editing.

## References

[1] Roth GA Mensah GA Johnson CO Addolorato G Ammirati E Baddour LM. Global burden of cardiovascular diseases and risk factors, 1990–2019: update from the GBD 2019 study. J Am Coll Cardiol. (2020) 76:2982–3021. doi: 10.1016/j.jacc.2020.11.010, PMID: 33309175 PMC7755038

[2] Nicholls M. Coronary artery disease in diabetes. Eur Heart J. (2017) 38:466–7. doi: 10.1093/eurheartj/ehx018 28363211

[3] Budoff MJ Raggi P Beller GA Berman DS Druz RS Malik S. Noninvasive cardiovascular risk assessment of the asymptomatic diabetic patient: the imaging Council of the American College of cardiology. JACC Cardiovasc Imaging. (2016) 9:176–92. doi: 10.1016/j.jcmg.2015.11.011, PMID: 26846937 PMC5371352

[4] Vaduganathan M Mensah GA Turco JV Fuster V Roth GA. The global burden of cardiovascular diseases and risk: a compass for future health. J Am Coll Cardiol. (2022) 80:2361–71. doi: 10.1016/j.jacc.2022.11.005 36368511

[5] Sotos-Prieto M Bhupathiraju SN Mattei J Fung TT Li Y Pan A. Association of Changes in diet quality with Total and cause-specific mortality. N Engl J Med. (2017) 377:143–53. doi: 10.1056/NEJMoa1613502, PMID: 28700845 PMC5589446

[6] Imran TF Kim E Buring JE Lee IM Gaziano JM Djousse L. Nut consumption, risk of cardiovascular mortality, and potential mediating mechanisms: the Women’s health study. J Clin Lipidol. (2021) 15:266–74. doi: 10.1016/j.jacl.2021.01.001, PMID: 33500188 PMC8666004

[7] Leong DP Smyth A Teo KK McKee M Rangarajan S Pais P. Patterns of alcohol consumption and myocardial infarction risk: observations from 52 countries in the INTERHEART case-control study. Circulation. (2014) 130:390–8. doi: 10.1161/CIRCULATIONAHA.113.007627 24928682

[8] Veronesi G Tunstall-Pedoe H Ferrario MM Kee F Kuulasmaa K Chambless LE. Combined effect of educational status and cardiovascular risk factors on the incidence of coronary heart disease and stroke in European cohorts: implications for prevention. Eur J Prev Cardiol. (2017) 24:437–45. doi: 10.1177/2047487316679521, PMID: 27837152

[9] Stringhini S Carmeli C Jokela M Avendaño M Muennig P Guida F. Socioeconomic status and the 25 × 25 risk factors as determinants of premature mortality: a multicohort study and meta-analysis of 1·7 million men and women. Lancet. (2017) 389:1229–37. doi: 10.1016/S0140-6736(16)32380-7, PMID: 28159391 PMC5368415

[10] Chagas P Mazocco L Piccoli JCE Ardenghi TM Badimon L Caramori PRA. Association of alcohol consumption with coronary artery disease severity. Clin Nutr. (2017) 36:1036–9. doi: 10.1016/j.clnu.2016.06.017 27402474

[11] Wang D Li W Cui X Meng Y Zhou M Xiao L. Sleep duration and risk of coronary heart disease: a systematic review and meta-analysis of prospective cohort studies. Int J Cardiol. (2016) 219:231–9. doi: 10.1016/j.ijcard.2016.06.027, PMID: 27336192

[12] Wang Y Huang L Zhou LX. Correlation between exercise, personal income level and health-related quality of life in patients with newly diagnosed stable angina. Mil Med Res. (2019) 6:36. doi: 10.1186/s40779-019-0226-5, PMID: 31760944 PMC6876075

[13] Roerecke M Rehm J. Alcohol consumption, drinking patterns, and ischemic heart disease: a narrative review of meta-analyses and a systematic review and meta-analysis of the impact of heavy drinking occasions on risk for moderate drinkers. BMC Med. (2014) 12:182. doi: 10.1186/s12916-014-0182-6, PMID: 25567363 PMC4203905

[14] Dudbridge F. Polygenic Mendelian randomization. Cold Spring Harb Perspect Med. (2021) 11:a039586. doi: 10.1101/cshperspect.a039586, PMID: 32229610 PMC7849343

[15] Hu Q Hao P Liu Q Dong M Gong Y Zhang C. Mendelian randomization studies on atherosclerotic cardiovascular disease: evidence and limitations. Sci China Life Sci. (2019) 62:758–70. doi: 10.1007/s11427-019-9537-4, PMID: 31104264

[16] Skrivankova VW Richmond RC Woolf BAR Yarmolinsky J Davies NM Swanson SA. Strengthening the reporting of observational studies in epidemiology using Mendelian randomization: the STROBE-MR statement. JAMA. (2021) 326:1614–21. doi: 10.1001/jama.2021.18236, PMID: 34698778

[17] Davies NM Holmes MV Davey SG. Reading Mendelian randomisation studies: a guide, glossary, and checklist for clinicians. BMJ. (2018) 362:k601. doi: 10.1136/bmj.k601, PMID: 30002074 PMC6041728

[18] Emdin CA Khera AV Kathiresan S. Mendelian randomization. JAMA. (2017) 318:1925–6. doi: 10.1001/jama.2017.17219 29164242

[19] Lee JJ Wedow R Okbay A Kong E Maghzian O Zacher M. Gene discovery and polygenic prediction from a genome-wide association study of educational attainment in 1.1 million individuals. Nat Genet. (2018) 50:1112–21. doi: 10.1038/s41588-018-0147-3, PMID: 30038396 PMC6393768

[20] Liu M Jiang Y Wedow R Li Y Brazel DM Chen F. Association studies of up to 1.2 million individuals yield new insights into the genetic etiology of tobacco and alcohol use. Nat Genet. (2019) 51:237–44. doi: 10.1038/s41588-018-0307-5, PMID: 30643251 PMC6358542

[21] Zhong VW Kuang A Danning RD Kraft P van Dam RM Chasman DI. A genome-wide association study of bitter and sweet beverage consumption. Hum Mol Genet. (2019) 28:2449–57. doi: 10.1093/hmg/ddz061, PMID: 31046077 PMC6606847

[22] Klimentidis YC Raichlen DA Bea J Garcia DO Wineinger NE Mandarino LJ. Genome-wide association study of habitual physical activity in over 377,000 UK biobank participants identifies multiple variants including CADM2 and APOE. Int J Obes. (2018) 42:1161–76. doi: 10.1038/s41366-018-0120-3, PMID: 29899525 PMC6195860

[23] Wootton RE Richmond RC Stuijfzand BG Lawn RB Sallis HM Taylor GMJ. Evidence for causal effects of lifetime smoking on risk for depression and schizophrenia: a Mendelian randomisation study. Psychol Med. (2020) 50:2435–43. doi: 10.1017/S0033291719002678, PMID: 31689377 PMC7610182

[24] Jansen PR Watanabe K Stringer S Skene N Bryois J Hammerschlag AR. Genome-wide analysis of insomnia in 1,331,010 individuals identifies new risk loci and functional pathways. Nat Genet. (2019) 51:394–403. doi: 10.1038/s41588-018-0333-3 30804565

[25] Fall T Gustafsson S Orho-Melander M Ingelsson E. Genome-wide association study of coronary artery disease among individuals with diabetes: the UK biobank. Diabetologia. (2018) 61:2174–9. doi: 10.1007/s00125-018-4686-z, PMID: 30003307 PMC6133153

[26] Eastwood SV Mathur R Atkinson M Brophy S Sudlow C Flaig R. Algorithms for the capture and adjudication of prevalent and incident diabetes in UK biobank. PLoS One. (2016) 11:e0162388. doi: 10.1371/journal.pone.0162388, PMID: 27631769 PMC5025160

[27] Clarke L Zheng-Bradley X Smith R Kulesha E Xiao C Toneva I. The 1000 genomes project: data management and community access. Nat Methods. (2012) 9:459–62. doi: 10.1038/nmeth.1974, PMID: 22543379 PMC3340611

[28] Staley JR Blackshaw J Kamat MA Ellis S Surendran P Sun BB. PhenoScanner: a database of human genotype-phenotype associations. Bioinformatics. (2016) 32:3207–9. doi: 10.1093/bioinformatics/btw373, PMID: 27318201 PMC5048068

[29] Burgess S Butterworth A Thompson SG. Mendelian randomization analysis with multiple genetic variants using summarized data. Genet Epidemiol. (2013) 37:658–65. doi: 10.1002/gepi.21758, PMID: 24114802 PMC4377079

[30] Bowden J Del Greco MF Minelli C Davey Smith G Sheehan N Thompson J. A framework for the investigation of pleiotropy in two-sample summary data Mendelian randomization. Stat Med. (2017) 36:1783–802. doi: 10.1002/sim.7221, PMID: 28114746 PMC5434863

[31] Bowden J Davey Smith G Haycock PC Burgess S. Consistent estimation in Mendelian randomization with some invalid instruments using a weighted median estimator. Genet Epidemiol. (2016) 40:304–14. doi: 10.1002/gepi.21965, PMID: 27061298 PMC4849733

[32] Bowden J Davey Smith G Burgess S. Mendelian randomization with invalid instruments: effect estimation and bias detection through Egger regression. Int J Epidemiol. (2015) 44:512–25. doi: 10.1093/ije/dyv080, PMID: 26050253 PMC4469799

[33] Zhao Q Chen Y Wang J Small DS. Powerful three-sample genome-wide design and robust statistical inference in summary-data Mendelian randomization. Int J Epidemiol. (2019) 48:1478–92. doi: 10.1093/ije/dyz142, PMID: 31298269

[34] Verbanck M Chen CY Neale B Do R. Detection of widespread horizontal pleiotropy in causal relationships inferred from Mendelian randomization between complex traits and diseases. Nat Genet. (2018) 50:693–8. doi: 10.1038/s41588-018-0099-7, PMID: 29686387 PMC6083837

[35] Armstrong RA. When to use the Bonferroni correction. Ophthalmic Physiol Opt. (2014) 34:502–8. doi: 10.1111/opo.12131 24697967

[36] Schunkert H Pang S Li L Paré G. Tracing risk of multiple cardiovascular diseases to smoking-related genes. Eur Heart J. (2020) 41:3311–3. doi: 10.1093/eurheartj/ehaa285, PMID: 32357238

[37] Pan A Wang Y Talaei M Hu FB. Relation of smoking with total mortality and cardiovascular events among patients with diabetes mellitus: a meta-analysis and systematic review. Circulation. (2015) 132:1795–804. doi: 10.1161/CIRCULATIONAHA.115.017926, PMID: 26311724 PMC4643392

[38] Chen S Yang F Xu T Wang Y Zhang K Fu G. Smoking and coronary artery disease risk in patients with diabetes: a Mendelian randomization study. Front Immunol. (2023) 14:891947. doi: 10.3389/fimmu.2023.891947, PMID: 36776880 PMC9910331

[39] Ogilvie RP Patel SR. The epidemiology of sleep and diabetes. Curr Diab Rep. (2018) 18:82. doi: 10.1007/s11892-018-1055-8, PMID: 30120578 PMC6437687

[40] Zhang Y Liu C Xu Y Wang Y Zhang Y Jiang T. The relationship between sugar-sweetened beverages, sleep disorders, and diabesity. Front Endocrinol. (2022) 13:1041977. doi: 10.3389/fendo.2022.1041977 PMC9869278 36699031

[41] Sofi F Cesari F Casini A Macchi C Abbate R Gensini GF. Insomnia and risk of cardiovascular disease: a meta-analysis. Eur J Prev Cardiol. (2014) 21:57–64. doi: 10.1177/2047487312460020 22942213

[42] Polsky S Akturk HK. Alcohol consumption, diabetes risk, and cardiovascular disease within diabetes. Curr Diab Rep. (2017) 17:136. doi: 10.1007/s11892-017-0950-8 29103170

[43] Cao M Cui B. Association of Educational Attainment with Adiposity, type 2 diabetes, and coronary artery diseases: a Mendelian randomization study. Front Public Health. (2020) 8:112. doi: 10.3389/fpubh.2020.00112, PMID: 32391302 PMC7189805

[44] Rabi DM Edwards AL Svenson LW Graham MM Knudtson ML Ghali WA. Association of Median Household Income with Burden of coronary artery disease among individuals with diabetes. Circ. Cardiovasc. Qual. Outcomes. (2010) 3:48–53. doi: 10.1161/CIRCOUTCOMES.108.840611 20123671

[45] Li N Katzmarzyk PT Horswell R Zhang Y Li W Zhao W. BMI and coronary heart disease risk among low-income and underinsured diabetic patients. Diabetes Care. (2014) 37:3204–12. doi: 10.2337/dc14-1091, PMID: 25249653 PMC4237979

