# Supplementary material for: Genetically predicted lifestyle factors, socioeconomic status and risk of coronary artery disease in individuals with diabetes: a Mendelian randomization study
Source: Front Public Health. 2023 Dec 22;11:1284958. doi: 10.3389/fpubh.2023.1284958 (PMC10771329; doi:10.3389/fpubh.2023.1284958)
Supplement: Supplementary file 1 [file Data_Sheet_1.PDF]

Genetically predicted lifestyle factors, Socioeconomic status and risk for coronary artery disease in individuals with diabetes: a Mendelian randomization study

Contents:

Supplementary figure 1(a). Leave-one-out analysis in the MR analysis between smoking initiation and the risk of CAD in individuals with diabetes;

Supplementary figure 1(b). Leave-one-out analysis in the MR analysis between lifetime smoking index and the risk of CAD in individuals with diabetes;

Supplementary figure 1(c). Leave-one-out analysis in the MR analysis between alcohol drinking and the risk of CAD in individuals with diabetes;

Supplementary figure 1(d). Leave-one-out analysis in the MR analysis between sleep duration and the risk of CAD in individuals with diabetes;

Supplementary figure 1(e). Leave-one-out analysis in the MR analysis between insomnia and the risk of CAD in individuals with diabetes;

Supplementary figure 1(f). Leave-one-out analysis in the MR analysis between AMPA and the risk of CAD in individuals with diabetes;

Supplementary figure 1(g). Leave-one-out analysis in the MR analysis between coffee consumption and the risk of CAD in individuals with diabetes;

Supplementary figure 1(h). Leave-one-out analysis in the MR analysis between educational attainment and the risk of CAD in individuals with diabetes;

Supplementary figure 1(i). Leave-one-out analysis in the MR analysis between average total household income before tax and the risk of CAD in individuals with diabetes;

Supplementary figure 1(j). Leave-one-out analysis in the MR analysis between Townsend deprivation index and the risk of CAD in individuals with diabetes.

a

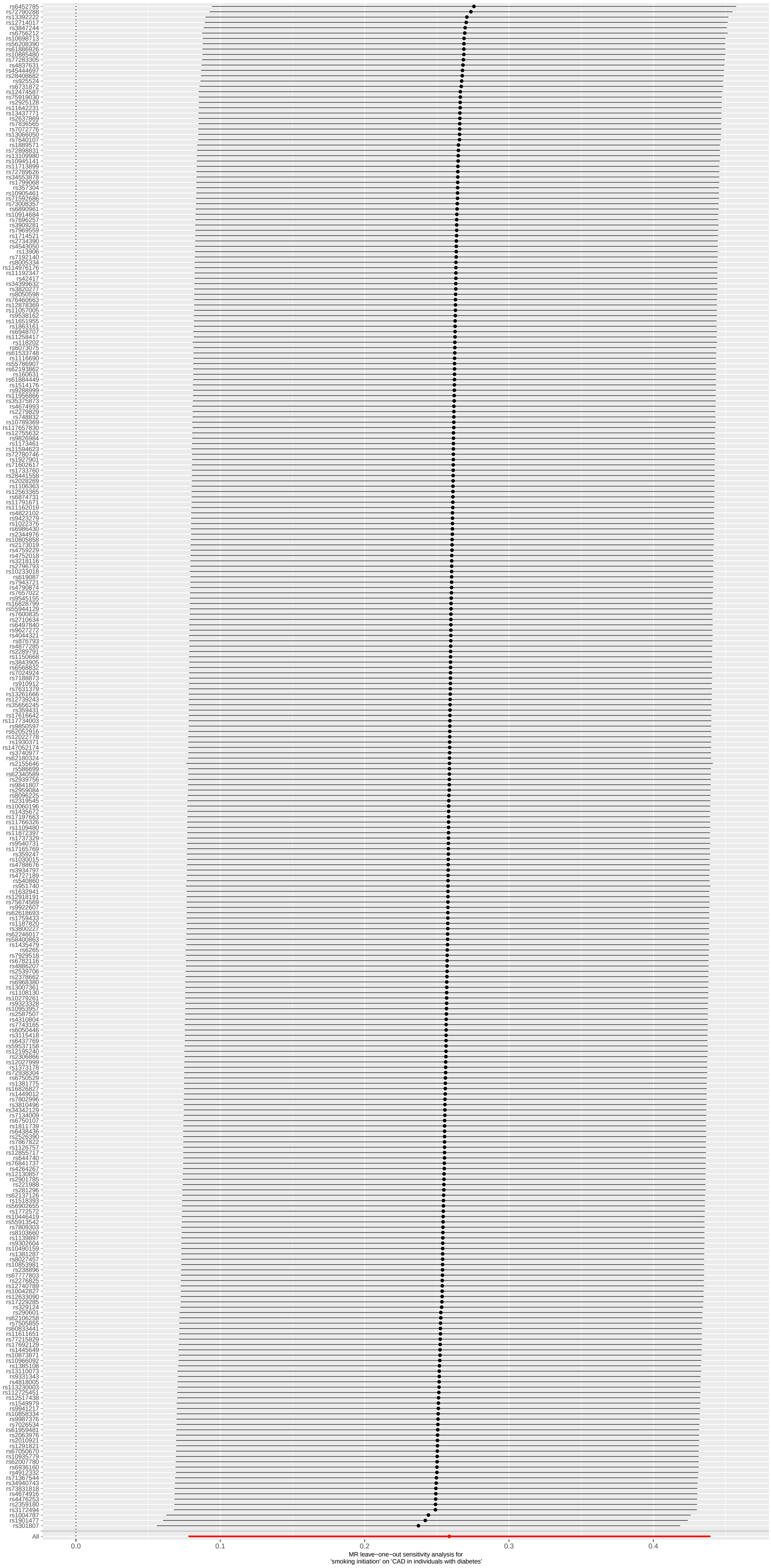
All
0.0
0.1
0.2
0.3
0.4
MR leave−one−out sensitivity analysis for
'smoking initiation' on 'CAD in individuals with diabetes'

b

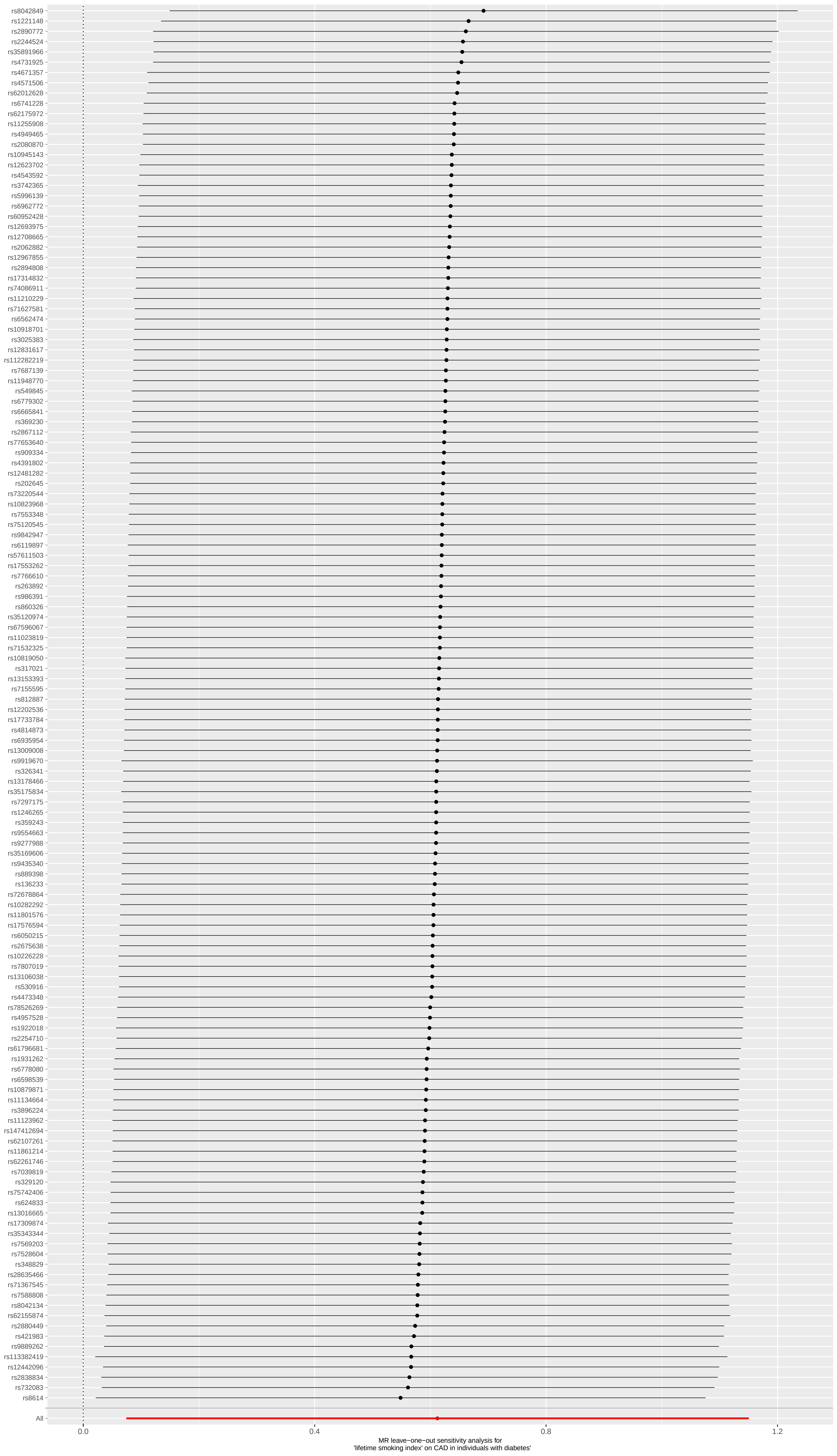
rs8042849
rs1221148
rs2890772
rs2244524
rs35891966
rs4731925
rs4671357
rs4571506
rs62012628
rs6741228
rs62175972
rs11255908
rs4949465
rs2080870
rs10945143
rs12623702
rs4543592
rs3742365
rs5996139
rs6962772
rs60952428
rs12693975
rs12708665
rs2062882
rs12967855
rs2894808
rs17314832
rs74086911
rs11210229
rs71627581
rs6562474
rs10918701
rs3025383
rs12831617
rs112282219
rs7687139
rs11948770
rs549845
rs6779302
rs6665841
rs369230
rs2867112
rs77653640
rs909334
rs4391802
rs12481282
rs202645
rs73220544
rs10823968
rs7553348
rs75120545
rs9842947
rs6119897
rs57611503
rs17553262
rs7766610
rs263892
rs986391
rs860326
rs35120974
rs67596067
rs11023819
rs71532325
rs10819050
rs317021
rs13153393
rs7155595
rs812887
rs12202536
rs17733784
rs4814873
rs6935954
rs13009008
rs9919670
rs326341
rs13178466
rs35175834
rs7297175
rs1246265
rs359243
rs9554663
rs9277988
rs35169606
rs9435340
rs889398
rs136233
rs72678864
rs10282292
rs11801576
rs17576594
rs6050215
rs2675638
rs10226228
rs7807019
rs13106038
rs530916
rs4473348
rs78526269
rs4957528
rs1922018
rs2254710
rs61796681
rs1931262
rs6778080
rs6598539
rs10879871
rs11134664
rs3896224
rs11123962
rs147412694
rs62107261
rs11861214
rs62261746
rs7039819
rs329120
rs75742406
rs624833
rs13016665
rs17309874
rs35343344
rs7569203
rs7528604
rs348829
rs28635466
rs71367545
rs7588808
rs8042134
rs62155874
rs2880449
rs421983
rs9889262
rs113382419
rs12442096
rs2838834
rs732083
rs8614
All
0.0
0.4
0.8
1.2
MR leave−one−out sensitivity analysis for
'lifetime smoking index' on CAD in individuals with diabetes'

c

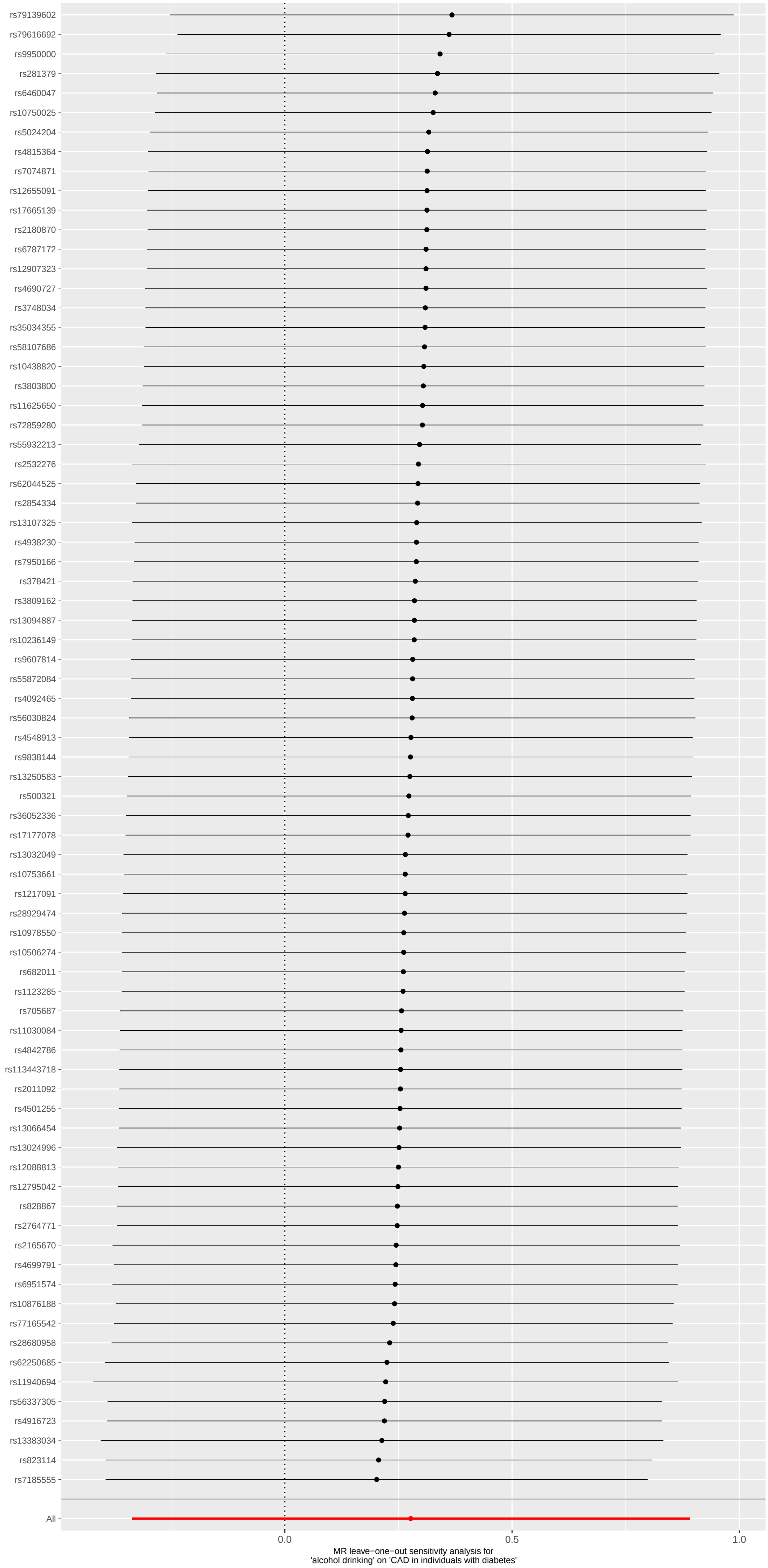
rs79139602
rs79616692
rs9950000
rs281379
rs6460047
rs10750025
rs5024204
rs4815364
rs7074871
rs12655091
rs17665139
rs2180870
rs6787172
rs12907323
rs4690727
rs3748034
rs35034355
rs58107686
rs10438820
rs3803800
rs11625650
rs72859280
rs55932213
rs2532276
rs62044525
rs2854334
rs13107325
rs4938230
rs7950166
rs378421
rs3809162
rs13094887
rs10236149
rs9607814
rs55872084
rs4092465
rs56030824
rs4548913
rs9838144
rs13250583
rs500321
rs36052336
rs17177078
rs13032049
rs10753661
rs1217091
rs28929474
rs10978550
rs10506274
rs682011
rs1123285
rs705687
rs11030084
rs4842786
rs113443718
rs2011092
rs4501255
rs13066454
rs13024996
rs12088813
rs12795042
rs828867
rs2764771
rs2165670
rs4699791
rs6951574
rs10876188
rs77165542
rs28680958
rs62250685
rs11940694
rs56337305
rs4916723
rs13383034
rs823114
rs7185555
All
0.0
0.5
1.0
MR leave−one−out sensitivity analysis for
'alcohol drinking' on 'CAD in individuals with diabetes'

d

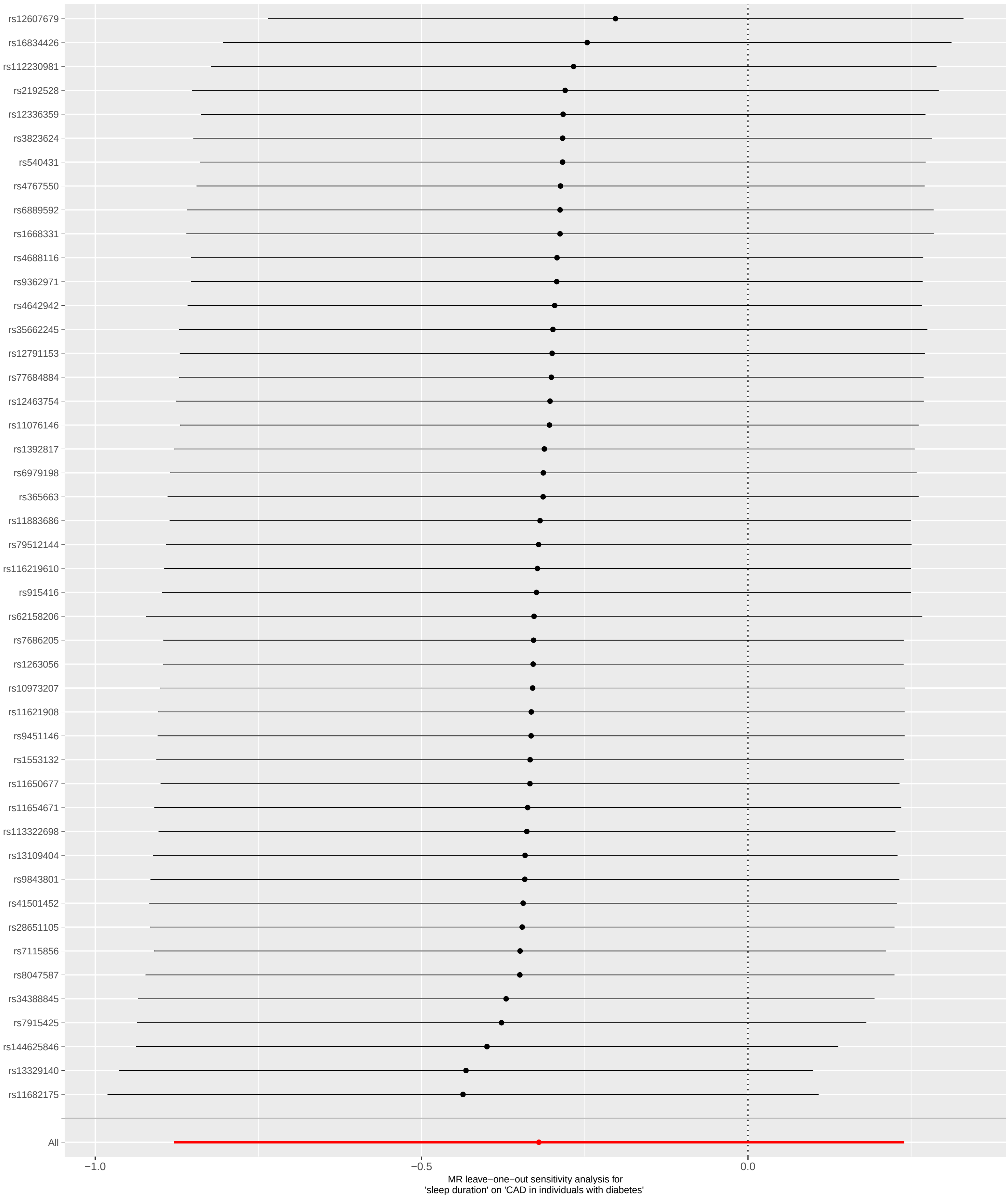
rs12607679
rs16834426
rs112230981
rs2192528
rs12336359
rs3823624
rs540431
rs4767550
rs6889592
rs1668331
rs4688116
rs9362971
rs4642942
rs35662245
rs12791153
rs77684884
rs12463754
rs11076146
rs1392817
rs6979198
rs365663
rs11883686
rs79512144
rs116219610
rs915416
rs62158206
rs7686205
rs1263056
rs10973207
rs11621908
rs9451146
rs1553132
rs11650677
rs11654671
rs113322698
rs13109404
rs9843801
rs41501452
rs28651105
rs7115856
rs8047587
rs34388845
rs7915425
rs144625846
rs13329140
rs11682175
All
−1.0
−0.5
0.0
MR leave−one−out sensitivity analysis for
'sleep duration' on 'CAD in individuals with diabetes'

e

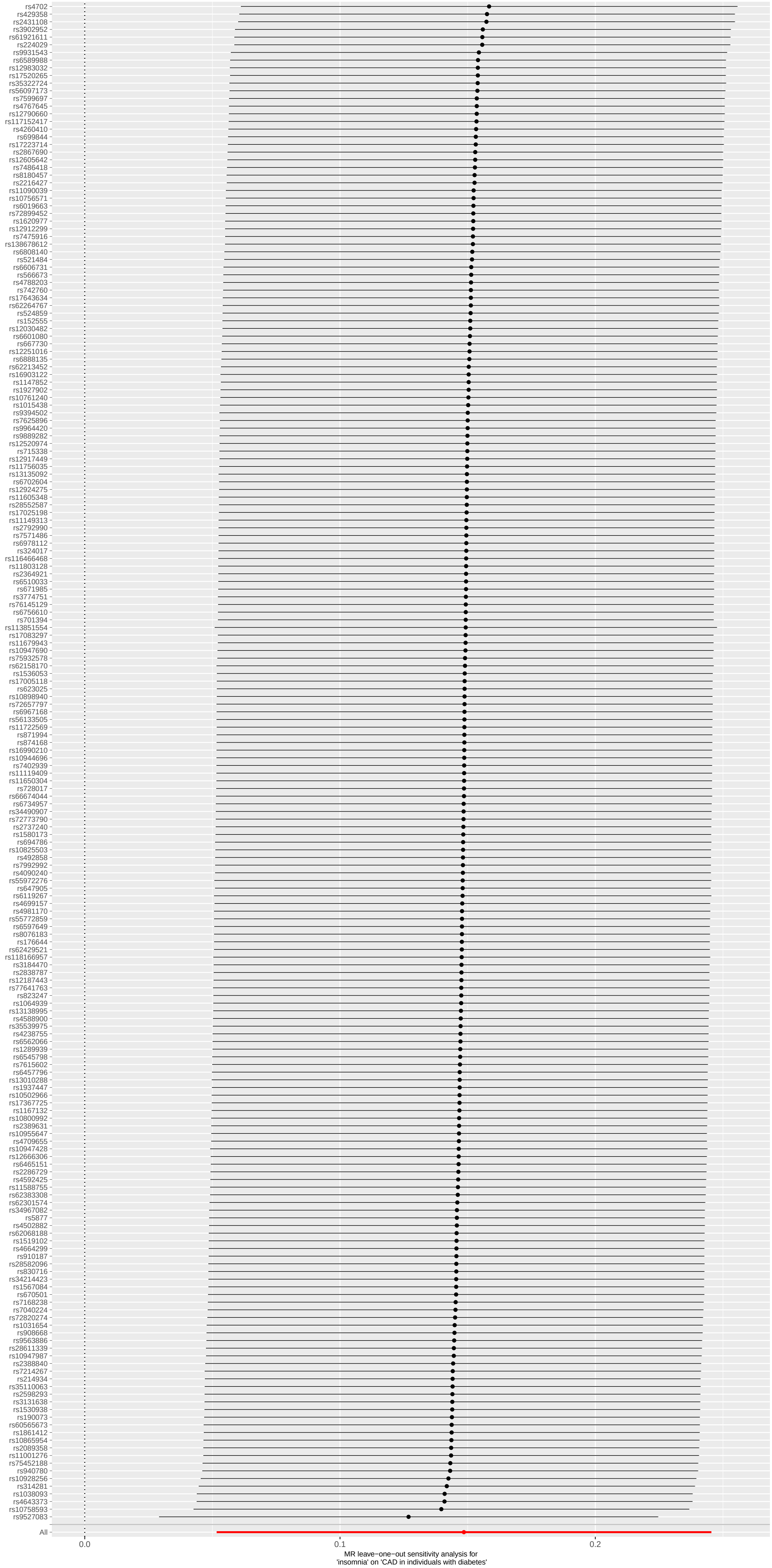
rs4702
rs429358
rs2431108
rs3902952
rs61921611
rs224029
rs9931543
rs6589988
rs12983032
rs17520265
rs35322724
rs56097173
rs7599697
rs4767645
rs12790660
rs117152417
rs4260410
rs699844
rs17223714
rs2867690
rs12605642
rs7486418
rs8180457
rs2216427
rs11090039
rs10756571
rs6019663
rs72899452
rs1620977
rs12912299
rs7475916
rs138678612
rs6808140
rs521484
rs6606731
rs566673
rs4788203
rs742760
rs17643634
rs62264767
rs524859
rs152555
rs12030482
rs6601080
rs667730
rs12251016
rs6888135
rs62213452
rs16903122
rs1147852
rs1927902
rs10761240
rs1015438
rs9394502
rs7625896
rs9964420
rs9889282
rs12520974
rs715338
rs12917449
rs11756035
rs13135092
rs6702604
rs12924275
rs11605348
rs28552587
rs17025198
rs11149313
rs2792990
rs7571486
rs6978112
rs324017
rs116466468
rs11803128
rs2364921
rs6510033
rs671985
rs3774751
rs76145129
rs6756610
rs701394
rs113851554
rs17083297
rs11679943
rs10947690
rs75932578
rs62158170
rs1536053
rs17005118
rs623025
rs10898940
rs72657797
rs6967168
rs56133505
rs11722569
rs871994
rs874168
rs16990210
rs10944696
rs7402939
rs11119409
rs11650304
rs728017
rs66674044
rs6734957
rs34490907
rs72773790
rs2737240
rs1580173
rs694786
rs10825503
rs492858
rs7992992
rs4090240
rs55972276
rs647905
rs6119267
rs4699157
rs4981170
rs55772859
rs6597649
rs8076183
rs176644
rs62429521
rs118166957
rs3184470
rs2838787
rs12187443
rs77641763
rs823247
rs1064939
rs13138995
rs4588900
rs35539975
rs4238755
rs6562066
rs1289939
rs6545798
rs7615602
rs6457796
rs13010288
rs1937447
rs10502966
rs17367725
rs1167132
rs10800992
rs2389631
rs10955647
rs4709655
rs10947428
rs12666306
rs6465151
rs2286729
rs4592425
rs11588755
rs62383308
rs62301574
rs34967082
rs5877
rs4502882
rs62068188
rs1519102
rs4664299
rs910187
rs28582096
rs830716
rs34214423
rs1567084
rs670501
rs7168238
rs7040224
rs72820274
rs1031654
rs908668
rs9563886
rs28611339
rs10947987
rs2388840
rs7214267
rs214934
rs35110063
rs2598293
rs3131638
rs1530938
rs190073
rs60565673
rs1861412
rs10865954
rs2089358
rs11001276
rs75452188
rs940780
rs10928256
rs314281
rs1038093
rs4643373
rs10758593
rs9527083
All
0.0
0.1
0.2
MR leave−one−out sensitivity analysis for
'insomnia' on 'CAD in individuals with diabetes'

f

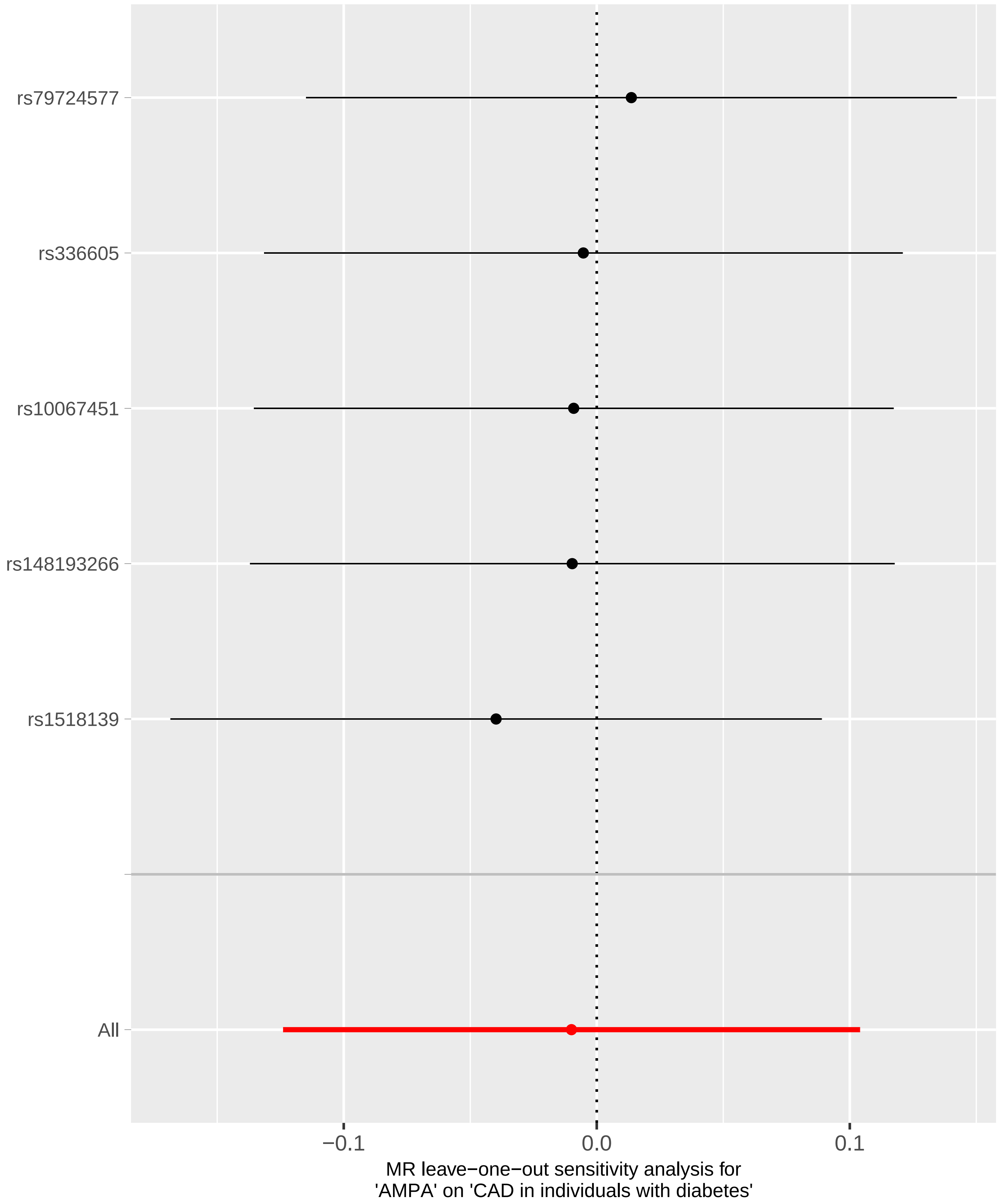

rs79724577
rs336605
rs10067451
rs148193266
rs1518139
All
−0.1
0.0
0.1
MR leave−one−out sensitivity analysis for
'AMPA' on 'CAD in individuals with diabetes'

g

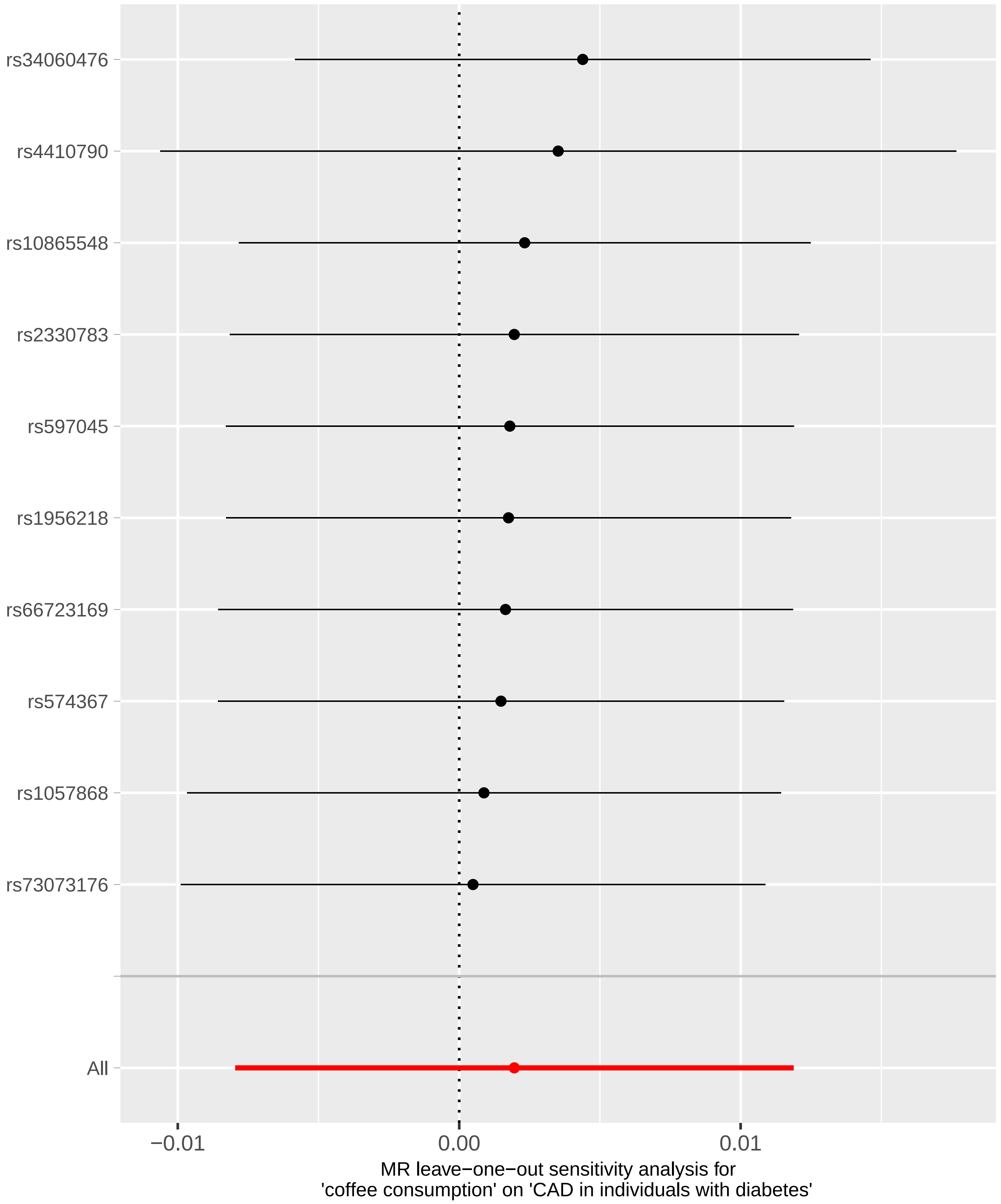

rs34060476
rs4410790
rs10865548
rs2330783
rs597045
rs1956218
rs66723169
rs574367
rs1057868
rs73073176
All
−0.01
0.00
0.01
MR leave−one−out sensitivity analysis for
'coffee consumption' on 'CAD in individuals with diabetes'

h

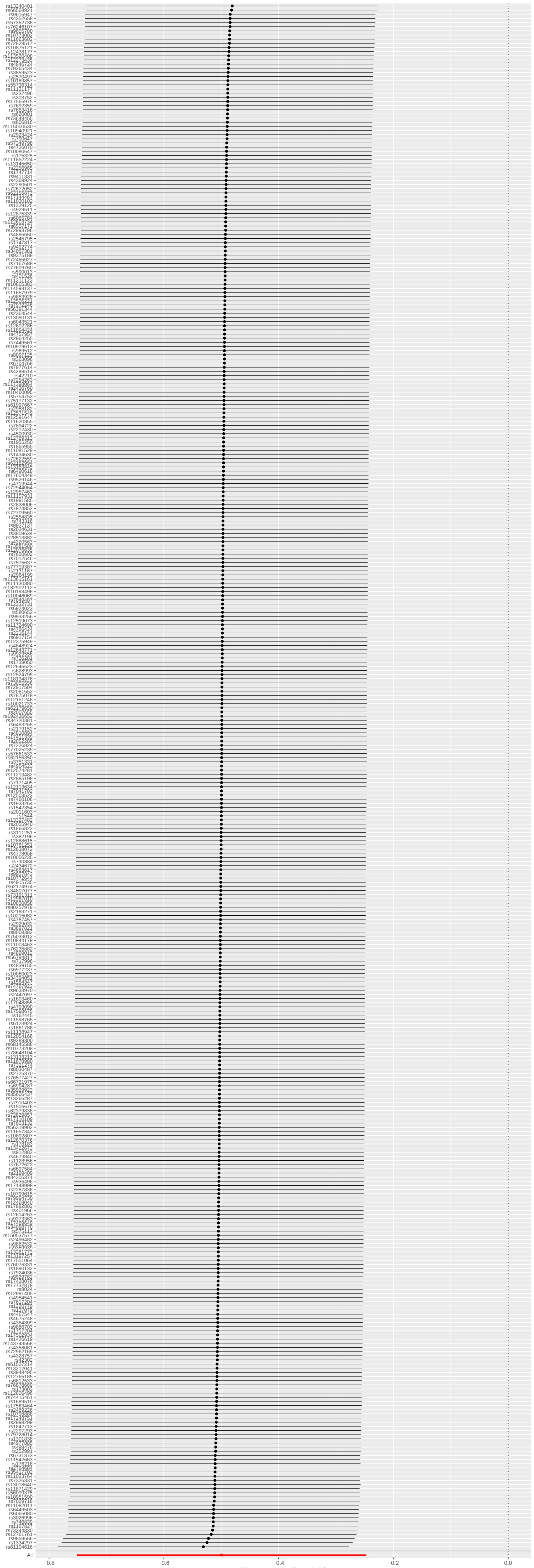

MR leave−one−out sensitivity analysis for
'educational attainment' on 'CAD in individuals with diabetes'

i

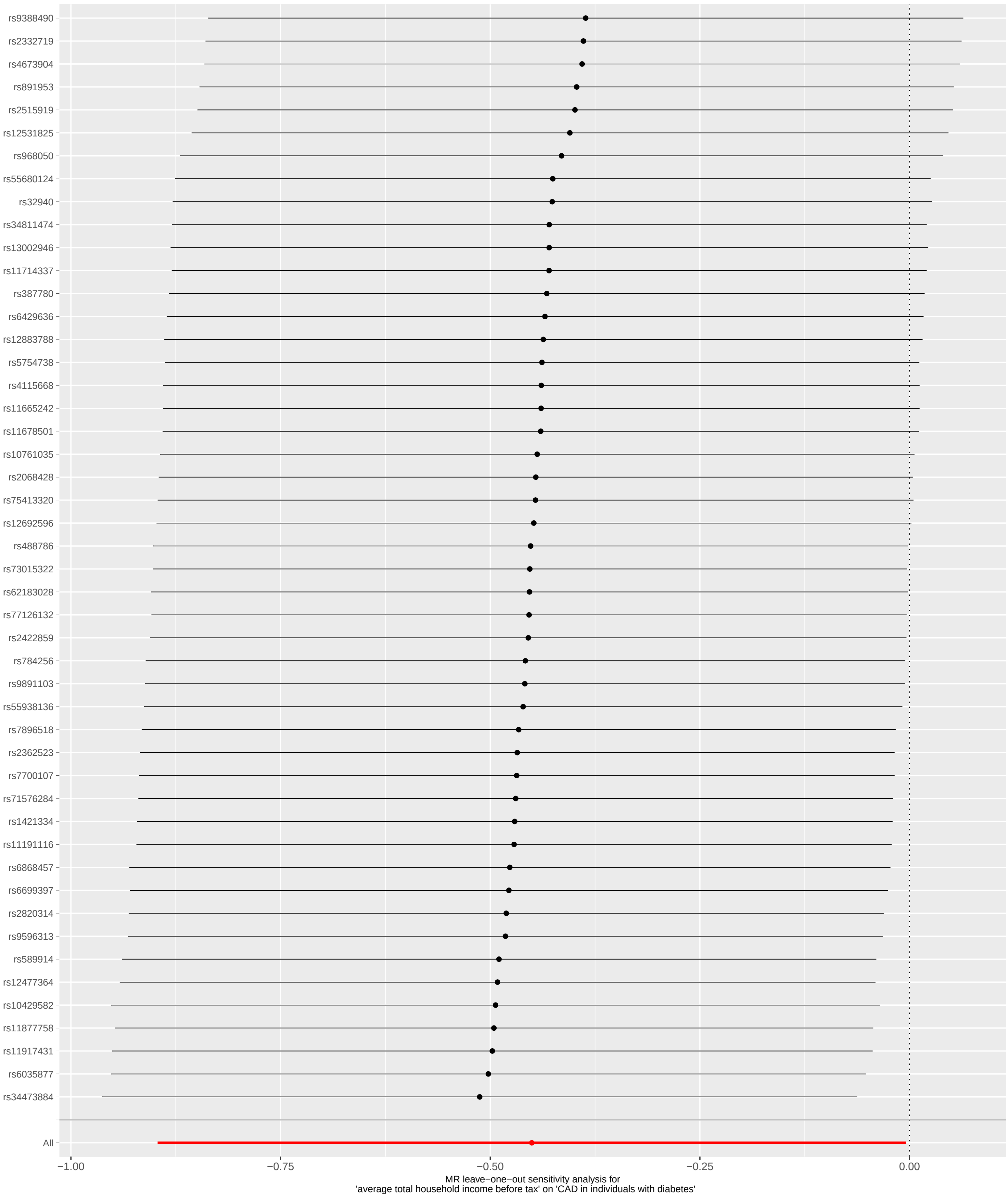
rs9388490
rs2332719
rs4673904
rs891953
rs2515919
rs12531825
rs968050
rs55680124
rs32940
rs34811474
rs13002946
rs11714337
rs387780
rs6429636
rs12883788
rs5754738
rs4115668
rs11665242
rs11678501
rs10761035
rs2068428
rs75413320
rs12692596
rs488786
rs73015322
rs62183028
rs77126132
rs2422859
rs784256
rs9891103
rs55938136
rs7896518
rs2362523
rs7700107
rs71576284
rs1421334
rs11191116
rs6868457
rs6699397
rs2820314
rs9596313
rs589914
rs12477364
rs10429582
rs11877758
rs11917431
rs6035877
rs34473884
All
−1.00
−0.75
−0.50
−0.25
0.00
MR leave−one−out sensitivity analysis for
'average total household income before tax' on 'CAD in individuals with diabetes'

j

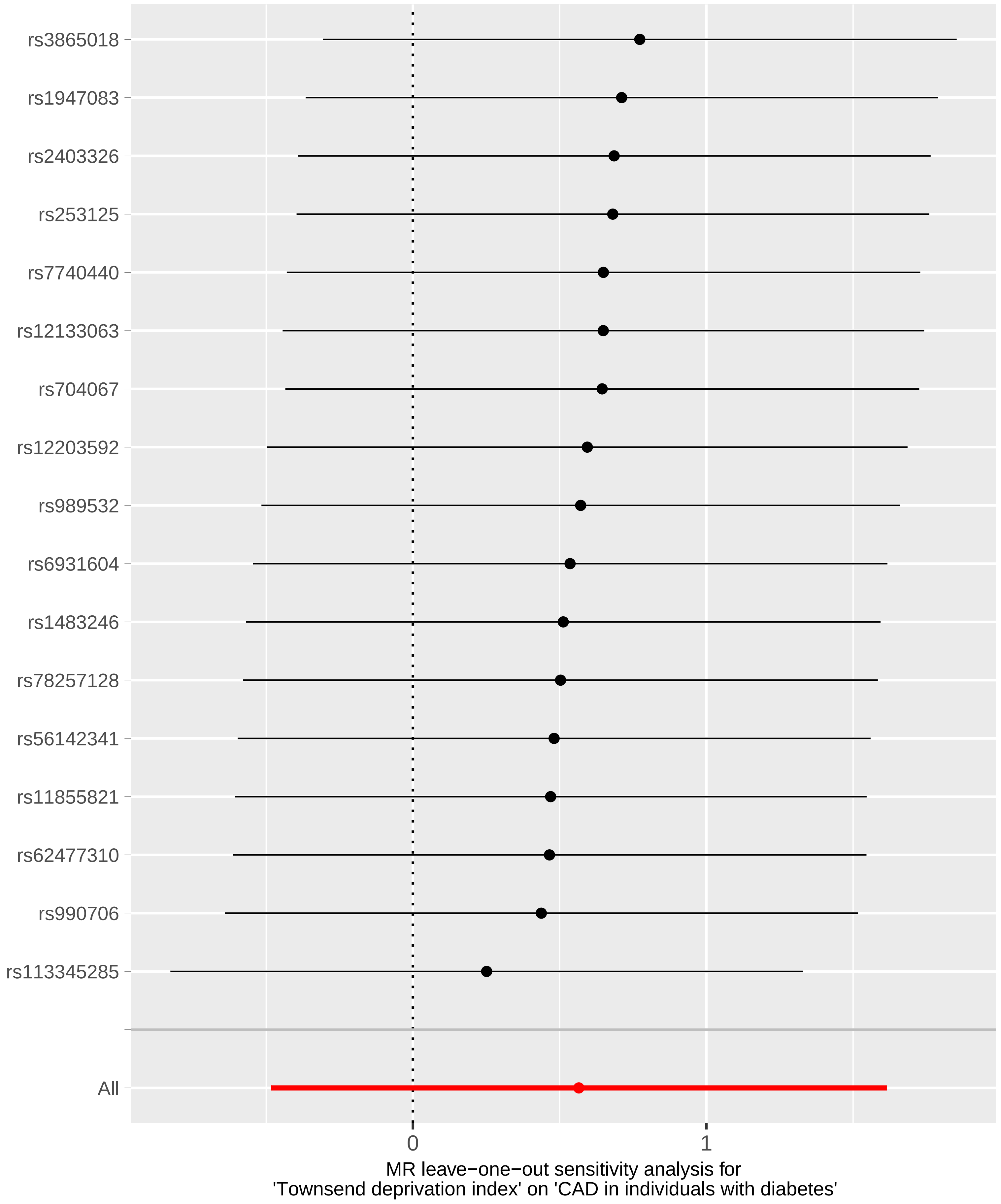

rs3865018
rs1947083
rs2403326
rs253125
rs7740440
rs12133063
rs704067
rs12203592
rs989532
rs6931604
rs1483246
rs78257128
rs56142341
rs11855821
rs62477310
rs990706
rs113345285
All
0
1
MR leave−one−out sensitivity analysis for
'Townsend deprivation index' on 'CAD in individuals with diabetes'
