# Supplementary material for: Genetically predicted lifestyle factors, socioeconomic status and risk of coronary artery disease in individuals with diabetes: a Mendelian randomization study
Source: Front Public Health. 2023 Dec 22;11:1284958. doi: 10.3389/fpubh.2023.1284958 (PMC10771329; doi:10.3389/fpubh.2023.1284958)
Supplement: Supplementary file 2 [file Table_1.docx]

**Genetically predicted lifestyle factors, socioeconomic status and risk**

**for coronary artery disease in individuals with diabetes: a Mendelian**

**randomization study**

**Contents:**

Supplementary table 1. Detailed description of exposure phenotypes;

Supplementary table 2. SNPs associated with smoking initiation at the genome-wide level of significance (*P* < 5x10^-8^) and clumped for independence at kb = 10,000 and *r*^2^ = 0.01;

Supplementary table 3. SNPs associated with lifetime smoking index at the genome-wide level of significance (*P* < 5x10^-8^) and clumped for independence at kb = 10,000 and *r*^2^ = 0.01;

Supplementary table 4. SNPs associated with alcohol drinking at the genome-wide level of significance (*P* < 5x10^-8^) and clumped for independence at kb = 10,000 and *r*^2^ = 0.01;

Supplementary table 5. SNPs associated with sleep duration at the genome-wide level of significance (*P* < 5x10^-8^) and clumped for independence at kb = 10,000 and *r*^2^ = 0.01;

Supplementary table 6. SNPs associated with insomnia at the genome-wide level of significance (*P* < 5x10^-8^) and clumped for independence at kb = 10,000 and *r*^2^ = 0.01;

Supplementary table 7. SNPs associated with AMPA at the genome-wide level of significance (*P* < 5x10^-8^) and clumped for independence at kb = 10,000 and *r*^2^ = 0.01;

Supplementary table 8. SNPs associated with coffee consumption at the genome-wide level of significance (*P* < 5x10^-8^) and clumped for independence at kb = 10,000 and *r*^2^ = 0.01;

Supplementary table 9. SNPs associated with educational attainment at the genome-wide level of significance (*P* < 5x10^-8^) and clumped for independence at kb = 10,000 and *r*^2^ = 0.001;

Supplementary table 10. SNPs associated with average total household income before tax at the genome-wide level of significance (*P* < 5x10^-8^) and clumped for independence at kb = 10,000 and *r*^2^ = 0.01;

Supplementary table 11. SNPs associated with Townsend deprivation index at the genome-wide level of significance (*P* < 5x10^-8^) and clumped for independence at kb = 10,000 and *r*^2^ = 0.01;

Supplementary table 12. SNPs associated with CAD in individuals with diabetes at the genome-wide level of significance (*P* < 5x10^-6^) and clumped for independence at kb = 10,000 and *r*^2^ = 0.01;

Supplementary table 13. The complementary MR analyses results of the causal effects of exposures on CAD in diabetic patients.

Supplementary table 14. The complementary MR analyses results of the causal effects of CAD in diabetic patients on outcomes.

**Supplementary table 1.** Detailed description of exposure phenotypes

| **phenotypes** | **Data source (PMID or Consortium)** | **Participants** | **Adjustments** | **Definition** |
| --- | --- | --- | --- | --- |
| **Lifestyle factors** |  |  |  |  |
| Smoking initiation | 30643251 | 1,232,091 European-descent individuals | Age, sex, and the first 10 genetic principal components | This phenotype was measured as binary variable with ever being a regular smoker in their life (current or former) coded as “2” and never being a regular smoker in their life coded as “1”. Participants were asked “Have you smoked over 100 cigarettes over the course of your life?”, “Have you ever smoked every day for at least a month?” or “Have you ever smoked regularly?”. |
| Lifetime smoking index | 31689377 | 462,690 European-descent individuals | Genotyping chip and sex | The lifetime smoking index was constructed by modelling the values of two constants: half-life and lag time. Together these constants capture the non-linear risk of smoking on health. Half-life captures the exponentially decreasing effect of smoking at a given time on health outcomes. Lag time accounts for the observation that smokers are more at risk of certain diseases immediately after stopping smoking than current smokers. |
| Alcohol drinking | 30643251 | 941,280 European-descent individuals | Age, sex, and the first 10 genetic principal components | This phenotype was measured as the average number of drinks a participant reported drinking each week, aggregated across all types of alcohol. Participants were asked “In the past week, how many alcoholic beverages did you have?” or “Thinking about the past year, on average how many drinks did you have each week?”. |
| Sleep duration | 30804565 | 384,317 European-descent individuals | Age, sex, genotype array, and 10 genetic principal components | Sleep duration was assessed by asking: “About how many hours sleep do you get in every 24 hours? (Include naps)". The answer could only contain integer values (round hours). Sleep duration was analyzed as a continuous outcome. Sleep duration was available in 384,317 unrelated individuals of European descent after quality control. The mean sleep duration was 7.10 (SD=1.30) hours per 24 hours. |
| Insomnia | 30804565 | 1,331,010 European-descent individuals | UK biobank:  Age, sex, genotype array, and 10 genetic principal components  23andMe:  Age, sex and the top 5 principal components | UK biobank: Participants were asked “Do you have trouble falling asleep at night or do you wake up in the middle of the night?” and were able to choose one of the following four answers: “never/rarely”, “sometimes”, “usually”, or “prefer not to answer”. Insomnia cases were defined as participants who answered this question with “usually”, while participants answering “never/rarely” or “sometimes” were defined as controls.  23andMe: Insomnia cases affirmed at least one of the following questions: "Have you ever been diagnosed with, or treated for: Insomnia?"; “Have you ever been diagnosed with, or treated for, any of the following conditions: Insomnia but not Narcolepsy, Sleep apnea or restless leg syndrome”; "Has a doctor ever told you that you have any of these conditions: Insomnia (difficulty getting to sleep or staying asleep)?"; "Have you ever been diagnosed by a doctor with any of the following neurological conditions: Sleep disturbance"; "Do you routinely have trouble getting to sleep at night?"; "What sleep disorders have you been diagnosed with? Please select all that apply: Insomnia, trouble falling or staying asleep"; "Have you ever taken these medications? Prescription sleep aids"; "In the last 2 years, have you taken any of these medications? Prescription sleep aids" |
| Average accelerometer-based physical activity | 29899525 | 91,084 European-descent individuals | Age, sex, center, PC 1-10, season, genotyping chip. | Accelerometer-based PA data (Axivity AX000 wrist-worn accelerometer) was collected in which participants were asked to wear the wrist-worn accelerometer for at least 18 hours at all times. |
| Coffee consumption | 31046077 | 375,833 European-descent individuals | Age, sex, body mass index, total energy, proportion of typical food intake, and 20 genetic principal components | This phenotype be defined as the intake of any type of coffee (i.e., regular/decaf, instant/ground). Touchscreen questionnaire at assessment center visit and Web-based 24h recall questionnaire. |
| **Socioeconomic status** |  |  |  |  |
| Educational attainment | 30038396 | 1,131,881 European-descent individuals | Sex, birth year, their interaction, and 10 principal components of the genetic relatedness matrix | Education attainment was measured as number of years of schooling completed. |
| Average total household income before tax | MRC‐IEU | 397,751 European-descent individuals | Age, sex, 10 genetic principal components | Participants were asked " What is the average total income before tax received by your HOUSEHOLD?" If you are unsure of your annual household income, here are the weekly and monthly equivalents. Per year: less than £18,000, £18,000 to £29,999, £30,000 to £51,999, £52,000 to £100,000, greater than £100,000. Monthly: below £1,500, £1,500 to £2,499, £2,500 to £4,333, £4,334 to £8,333, above £8,333 Weekly: below £346, 346 to £576, 577 to £999, 1000 to £1923, above £1923. |
| Townsend deprivation index at recruitment | MRC‐IEU | 462,464 European-descent individuals | Age, sex, 10 genetic principal components | Townsend deprivation index calculated immediately prior to participant joining UK Biobank. Based on the preceding national census output areas. Each participant is assigned a score corresponding to the output area in which their postcode is located. |

**Supplementary table 2.** SNPs associated with smoking initiation at the genome-wide level of significance (*P* < 5x10^-8^) and clumped for independence at kb = 10,000 and *r^2^* = 0.01

| **SNP** | **EA** | **OA** | **EAF** | **BETA** | **SE** | ***P*-value** | **R^2^ (%)** | **F** |
| --- | --- | --- | --- | --- | --- | --- | --- | --- |
| rs12130857 | A | G | 0.325 | -0.018 | 0.003 | 3.65E-11 | 0.0029 | 36.00 |
| rs301807 | G | A | 0.57 | 0.018 | 0.003 | 2.50E-12 | 0.0029 | 36.00 |
| rs3820277 | T | G | 0.526 | -0.019 | 0.003 | 1.57E-13 | 0.0033 | 40.11 |
| rs1889571 | G | T | 0.131 | 0.022 | 0.004 | 4.19E-09 | 0.0025 | 30.25 |
| rs10914684 | A | G | 0.324 | -0.016 | 0.003 | 6.32E-09 | 0.0023 | 28.44 |
| rs2637869 | A | G | 0.297 | 0.018 | 0.003 | 6.54E-11 | 0.0029 | 36.00 |
| rs12755632 | G | A | 0.316 | -0.015 | 0.003 | 1.93E-08 | 0.0020 | 25.00 |
| rs951740 | A | G | 0.625 | 0.03 | 0.003 | 3.82E-29 | 0.0081 | 100.00 |
| rs925524 | G | A | 0.71 | 0.016 | 0.003 | 2.94E-08 | 0.0023 | 28.44 |
| rs12022778 | C | A | 0.202 | 0.027 | 0.003 | 3.18E-17 | 0.0066 | 81.00 |
| rs4912332 | T | C | 0.491 | 0.014 | 0.003 | 2.94E-08 | 0.0018 | 21.78 |
| rs1937443 | G | C | 0.563 | 0.02 | 0.003 | 1.79E-15 | 0.0036 | 44.44 |
| rs12740789 | A | G | 0.178 | -0.028 | 0.003 | 1.18E-17 | 0.0071 | 87.11 |
| rs10789369 | G | A | 0.615 | -0.023 | 0.003 | 3.39E-19 | 0.0048 | 58.78 |
| rs1514176 | A | G | 0.58 | -0.019 | 0.003 | 7.67E-14 | 0.0033 | 40.11 |
| rs10873871 | G | A | 0.207 | 0.017 | 0.003 | 2.82E-08 | 0.0026 | 32.11 |
| rs11162019 | T | C | 0.363 | -0.015 | 0.003 | 5.06E-09 | 0.0020 | 25.00 |
| rs1008078 | T | C | 0.402 | 0.023 | 0.003 | 1.63E-18 | 0.0048 | 58.78 |
| rs12027999 | C | T | 0.12 | -0.024 | 0.004 | 5.33E-10 | 0.0029 | 36.00 |
| rs45444697 | G | C | 0.212 | 0.02 | 0.003 | 2.72E-10 | 0.0036 | 44.44 |
| rs2901785 | A | G | 0.446 | -0.017 | 0.003 | 1.47E-11 | 0.0026 | 32.11 |
| rs147052174 | T | G | 0.0171 | 0.062 | 0.01 | 2.30E-10 | 0.0031 | 38.44 |
| rs35656245 | A | G | 0.276 | 0.016 | 0.003 | 2.23E-08 | 0.0023 | 28.44 |
| rs12739243 | C | T | 0.221 | -0.021 | 0.003 | 4.45E-12 | 0.0040 | 49.00 |
| rs12563365 | A | G | 0.556 | 0.017 | 0.003 | 1.05E-10 | 0.0026 | 32.11 |
| rs876793 | C | T | 0.3493 | -0.018 | 0.003 | 5.69E-11 | 0.0029 | 36.00 |
| rs114976176 | C | A | 0.3516 | -0.016 | 0.003 | 6.04E-09 | 0.0023 | 28.44 |
| rs62106258 | C | T | 0.0473 | -0.045 | 0.006 | 3.33E-14 | 0.0046 | 56.25 |
| rs6731872 | G | T | 0.826 | 0.032 | 0.003 | 5.35E-21 | 0.0092 | 113.78 |
| rs1022376 | C | T | 0.5158 | -0.015 | 0.003 | 1.66E-08 | 0.0020 | 25.00 |
| rs61533748 | C | T | 0.384 | 0.017 | 0.003 | 2.82E-11 | 0.0026 | 32.11 |
| rs72790288 | A | G | 0.0282 | -0.046 | 0.008 | 3.28E-09 | 0.0027 | 33.06 |
| rs2710634 | C | T | 0.521 | -0.018 | 0.003 | 3.36E-12 | 0.0029 | 36.00 |
| rs62137126 | G | A | 0.1211 | -0.024 | 0.004 | 1.31E-09 | 0.0029 | 36.00 |
| rs1004787 | A | G | 0.552 | 0.028 | 0.003 | 1.11E-28 | 0.0071 | 87.11 |
| rs7598402 | G | C | 0.4921 | -0.015 | 0.003 | 7.38E-09 | 0.0020 | 25.00 |
| rs10490159 | T | C | 0.394 | 0.017 | 0.003 | 3.86E-11 | 0.0026 | 32.11 |
| rs1518393 | C | A | 0.619 | 0.017 | 0.003 | 1.30E-10 | 0.0026 | 32.11 |
| rs17616642 | G | A | 0.2469 | -0.017 | 0.003 | 2.10E-08 | 0.0026 | 32.11 |
| rs2539706 | A | G | 0.5299 | 0.016 | 0.003 | 1.95E-10 | 0.0023 | 28.44 |
| rs1863161 | A | G | 0.5609 | 0.015 | 0.003 | 2.34E-09 | 0.0020 | 25.00 |
| rs359247 | T | A | 0.6387 | 0.022 | 0.003 | 9.89E-17 | 0.0044 | 53.78 |
| rs62180324 | A | G | 0.212 | -0.02 | 0.003 | 3.91E-10 | 0.0036 | 44.44 |
| rs6750107 | A | G | 0.3869 | 0.015 | 0.003 | 2.60E-08 | 0.0020 | 25.00 |
| rs12714017 | C | T | 0.511 | 0.015 | 0.003 | 3.65E-09 | 0.0020 | 25.00 |
| rs56208390 | G | A | 0.123 | 0.022 | 0.004 | 2.68E-08 | 0.0025 | 30.25 |
| rs11692435 | A | G | 0.0848 | 0.025 | 0.005 | 4.47E-08 | 0.0020 | 25.00 |
| rs13392222 | C | A | 0.139 | -0.023 | 0.004 | 1.93E-10 | 0.0027 | 33.06 |
| rs1901477 | G | A | 0.511 | 0.03 | 0.003 | 2.07E-31 | 0.0081 | 100.00 |
| rs3811038 | C | T | 0.279 | 0.019 | 0.003 | 1.58E-11 | 0.0033 | 40.11 |
| rs34399632 | G | A | 0.232 | 0.019 | 0.003 | 1.46E-10 | 0.0033 | 40.11 |
| rs6756212 | T | C | 0.535 | -0.034 | 0.003 | 3.49E-40 | 0.0104 | 128.44 |
| rs16826827 | C | T | 0.124 | -0.022 | 0.004 | 9.17E-09 | 0.0025 | 30.25 |
| rs1445649 | C | T | 0.538 | 0.021 | 0.003 | 8.48E-16 | 0.0040 | 49.00 |
| rs12474587 | T | G | 0.429 | 0.024 | 0.003 | 4.83E-21 | 0.0052 | 64.00 |
| rs357304 | C | T | 0.727 | 0.017 | 0.003 | 5.40E-09 | 0.0026 | 32.11 |
| rs13007361 | A | G | 0.208 | 0.018 | 0.003 | 2.29E-08 | 0.0029 | 36.00 |
| rs7600835 | A | G | 0.342 | -0.015 | 0.003 | 1.80E-08 | 0.0020 | 25.00 |
| rs6750529 | T | C | 0.744 | 0.02 | 0.003 | 9.26E-12 | 0.0036 | 44.44 |
| rs17229285 | T | C | 0.505 | -0.015 | 0.003 | 1.27E-09 | 0.0020 | 25.00 |
| rs3115418 | C | T | 0.454 | -0.014 | 0.003 | 2.79E-08 | 0.0018 | 21.78 |
| rs62193862 | A | G | 0.0999 | 0.024 | 0.004 | 1.99E-08 | 0.0029 | 36.00 |
| rs4674916 | A | C | 0.3277 | -0.018 | 0.003 | 3.06E-11 | 0.0029 | 36.00 |
| rs4674993 | G | A | 0.2 | -0.024 | 0.003 | 4.85E-14 | 0.0052 | 64.00 |
| rs11713899 | C | A | 0.171 | 0.019 | 0.003 | 3.15E-08 | 0.0033 | 40.11 |
| rs748832 | G | A | 0.371 | 0.017 | 0.003 | 6.60E-11 | 0.0026 | 32.11 |
| rs10446419 | G | A | 0.207 | -0.02 | 0.003 | 5.05E-10 | 0.0036 | 44.44 |
| rs3172494 | T | G | 0.115 | -0.029 | 0.004 | 3.40E-13 | 0.0043 | 52.56 |
| rs2526390 | T | C | 0.334 | 0.02 | 0.003 | 3.62E-14 | 0.0036 | 44.44 |
| rs2276825 | C | T | 0.245 | 0.019 | 0.003 | 1.89E-10 | 0.0033 | 40.11 |
| rs2306866 | T | A | 0.614 | -0.017 | 0.003 | 1.89E-10 | 0.0026 | 32.11 |
| rs73831818 | G | A | 0.057 | 0.032 | 0.005 | 5.46E-09 | 0.0033 | 40.96 |
| rs7640107 | T | C | 0.4308 | -0.014 | 0.003 | 3.46E-08 | 0.0018 | 21.78 |
| rs2734390 | G | A | 0.372 | 0.015 | 0.003 | 2.09E-08 | 0.0020 | 25.00 |
| rs221988 | C | A | 0.384 | -0.015 | 0.003 | 1.43E-08 | 0.0020 | 25.00 |
| rs11128203 | A | T | 0.53 | 0.02 | 0.003 | 1.29E-15 | 0.0036 | 44.44 |
| rs62246017 | A | G | 0.3226 | -0.016 | 0.003 | 3.03E-09 | 0.0023 | 28.44 |
| rs4543050 | T | A | 0.816 | 0.022 | 0.003 | 1.45E-11 | 0.0044 | 53.78 |
| rs6782116 | T | C | 0.415 | -0.015 | 0.003 | 1.46E-08 | 0.0020 | 25.00 |
| rs13066050 | T | C | 0.208 | 0.019 | 0.003 | 1.93E-09 | 0.0033 | 40.11 |
| rs12633090 | C | G | 0.182 | -0.023 | 0.003 | 3.16E-12 | 0.0048 | 58.78 |
| rs1549979 | T | C | 0.615 | -0.025 | 0.003 | 8.80E-21 | 0.0056 | 69.44 |
| rs6437769 | T | C | 0.581 | 0.014 | 0.003 | 3.74E-08 | 0.0018 | 21.78 |
| rs9288999 | A | G | 0.735 | 0.017 | 0.003 | 1.50E-09 | 0.0026 | 32.11 |
| rs6438436 | T | C | 0.816 | 0.025 | 0.003 | 5.33E-14 | 0.0056 | 69.44 |
| rs9826984 | A | G | 0.542 | -0.014 | 0.003 | 3.87E-08 | 0.0018 | 21.78 |
| rs2279829 | T | C | 0.216 | -0.017 | 0.003 | 2.05E-08 | 0.0026 | 32.11 |
| rs2319545 | A | C | 0.1491 | 0.023 | 0.004 | 8.30E-11 | 0.0027 | 33.06 |
| rs10935779 | T | C | 0.415 | -0.014 | 0.003 | 2.95E-08 | 0.0018 | 21.78 |
| rs1714521 | C | A | 0.411 | -0.016 | 0.003 | 3.07E-10 | 0.0023 | 28.44 |
| rs1449012 | T | C | 0.463 | -0.015 | 0.003 | 1.77E-09 | 0.0020 | 25.00 |
| rs9850597 | A | G | 0.816 | -0.019 | 0.003 | 1.65E-08 | 0.0033 | 40.11 |
| rs1187820 | T | C | 0.439 | -0.014 | 0.003 | 2.69E-08 | 0.0018 | 21.78 |
| rs16828799 | T | G | 0.156 | 0.02 | 0.004 | 1.83E-08 | 0.0020 | 25.00 |
| rs9841807 | T | C | 0.273 | 0.016 | 0.003 | 1.35E-08 | 0.0023 | 28.44 |
| rs7631379 | C | T | 0.206 | 0.021 | 0.003 | 3.94E-11 | 0.0040 | 49.00 |
| rs4140932 | A | T | 0.431 | -0.014 | 0.003 | 4.89E-08 | 0.0018 | 21.78 |
| rs59537158 | T | C | 0.214 | 0.022 | 0.003 | 4.62E-13 | 0.0044 | 53.78 |
| rs55944129 | C | T | 0.267 | -0.018 | 0.003 | 1.06E-09 | 0.0029 | 36.00 |
| rs58400863 | A | G | 0.347 | -0.02 | 0.003 | 4.89E-14 | 0.0036 | 44.44 |
| rs7657022 | G | A | 0.489 | 0.018 | 0.003 | 7.34E-13 | 0.0029 | 36.00 |
| rs112725451 | T | C | 0.169 | 0.026 | 0.003 | 1.65E-14 | 0.0061 | 75.11 |
| rs1160685 | G | C | 0.45 | 0.015 | 0.003 | 2.31E-09 | 0.0020 | 25.00 |
| rs1435479 | T | G | 0.2875 | 0.016 | 0.003 | 5.68E-09 | 0.0023 | 28.44 |
| rs3934797 | A | G | 0.182 | -0.021 | 0.003 | 1.12E-10 | 0.0040 | 49.00 |
| rs71602617 | T | C | 0.216 | -0.018 | 0.003 | 2.10E-08 | 0.0029 | 36.00 |
| rs7696257 | A | G | 0.366 | 0.015 | 0.003 | 6.78E-09 | 0.0020 | 25.00 |
| rs13109980 | A | G | 0.326 | -0.022 | 0.003 | 3.37E-16 | 0.0044 | 53.78 |
| rs1116690 | G | A | 0.742 | 0.016 | 0.003 | 2.16E-08 | 0.0023 | 28.44 |
| rs13110073 | C | T | 0.395 | -0.025 | 0.003 | 3.24E-21 | 0.0056 | 69.44 |
| rs62340589 | C | G | 0.201 | 0.017 | 0.003 | 4.31E-08 | 0.0026 | 32.11 |
| rs12517438 | G | T | 0.538 | 0.015 | 0.003 | 1.89E-09 | 0.0020 | 25.00 |
| rs35375873 | C | G | 0.11 | -0.027 | 0.004 | 3.29E-11 | 0.0037 | 45.56 |
| rs986714 | T | A | 0.445 | -0.016 | 0.003 | 4.13E-10 | 0.0023 | 28.44 |
| rs71592686 | C | T | 0.274 | 0.021 | 0.003 | 3.85E-13 | 0.0040 | 49.00 |
| rs2028269 | A | G | 0.399 | 0.016 | 0.003 | 5.19E-10 | 0.0023 | 28.44 |
| rs6874731 | G | T | 0.484 | 0.015 | 0.003 | 1.83E-09 | 0.0020 | 25.00 |
| rs6452785 | T | C | 0.474 | -0.027 | 0.003 | 4.69E-26 | 0.0066 | 81.00 |
| rs10805858 | T | A | 0.3353 | 0.018 | 0.003 | 1.88E-11 | 0.0029 | 36.00 |
| rs42417 | T | C | 0.691 | 0.017 | 0.003 | 8.27E-10 | 0.0026 | 32.11 |
| rs72780746 | C | T | 0.173 | -0.026 | 0.003 | 2.05E-14 | 0.0061 | 75.11 |
| rs10060196 | A | C | 0.5806 | 0.018 | 0.003 | 1.29E-12 | 0.0029 | 36.00 |
| rs72789626 | A | T | 0.136 | -0.026 | 0.004 | 5.13E-12 | 0.0034 | 42.25 |
| rs17165769 | G | A | 0.3949 | 0.016 | 0.003 | 9.56E-10 | 0.0023 | 28.44 |
| rs329124 | G | A | 0.428 | -0.016 | 0.003 | 1.96E-10 | 0.0023 | 28.44 |
| rs1385108 | T | C | 0.239 | 0.019 | 0.003 | 3.84E-10 | 0.0033 | 40.11 |
| rs1173461 | T | C | 0.327 | 0.017 | 0.003 | 9.51E-10 | 0.0026 | 32.11 |
| rs11956866 | G | T | 0.567 | -0.015 | 0.003 | 7.82E-09 | 0.0020 | 25.00 |
| rs3909281 | G | T | 0.536 | 0.021 | 0.003 | 1.62E-16 | 0.0040 | 49.00 |
| rs3843905 | T | C | 0.403 | -0.015 | 0.003 | 5.41E-09 | 0.0020 | 25.00 |
| rs6890961 | T | C | 0.624 | -0.019 | 0.003 | 2.13E-13 | 0.0033 | 40.11 |
| rs4044321 | G | A | 0.644 | -0.023 | 0.003 | 1.75E-17 | 0.0048 | 58.78 |
| rs2173019 | A | T | 0.177 | 0.028 | 0.003 | 2.98E-17 | 0.0071 | 87.11 |
| rs10042827 | C | T | 0.681 | 0.017 | 0.003 | 9.41E-10 | 0.0026 | 32.11 |
| rs359431 | T | C | 0.56 | -0.014 | 0.003 | 3.16E-08 | 0.0018 | 21.78 |
| rs1150668 | G | T | 0.419 | -0.019 | 0.003 | 8.54E-13 | 0.0033 | 40.11 |
| rs1632941 | C | T | 0.46 | -0.016 | 0.003 | 6.67E-10 | 0.0023 | 28.44 |
| rs3218116 | T | C | 0.256 | -0.02 | 0.003 | 1.05E-11 | 0.0036 | 44.44 |
| rs160631 | G | T | 0.731 | -0.017 | 0.003 | 1.87E-09 | 0.0026 | 32.11 |
| rs7743165 | G | T | 0.495 | 0.019 | 0.003 | 4.15E-14 | 0.0033 | 40.11 |
| rs10945141 | A | G | 0.263 | 0.018 | 0.003 | 3.59E-10 | 0.0029 | 36.00 |
| rs17554906 | C | G | 0.444 | 0.014 | 0.003 | 3.14E-08 | 0.0018 | 21.78 |
| rs619087 | G | A | 0.422 | 0.014 | 0.003 | 3.10E-08 | 0.0018 | 21.78 |
| rs6568832 | A | G | 0.7539 | 0.019 | 0.003 | 1.74E-10 | 0.0033 | 40.11 |
| rs12195240 | A | G | 0.285 | 0.025 | 0.003 | 1.08E-18 | 0.0056 | 69.44 |
| rs6936160 | T | C | 0.698 | 0.02 | 0.003 | 4.20E-13 | 0.0036 | 44.44 |
| rs3800227 | G | A | 0.742 | 0.017 | 0.003 | 3.64E-09 | 0.0026 | 32.11 |
| rs118202 | T | G | 0.812 | -0.037 | 0.003 | 1.90E-29 | 0.0123 | 152.11 |
| rs73008357 | C | A | 0.121 | -0.022 | 0.004 | 2.44E-08 | 0.0025 | 30.25 |
| rs9331343 | C | T | 0.568 | -0.014 | 0.003 | 3.90E-08 | 0.0018 | 21.78 |
| rs10698713 | A | G | 0.0544 | -0.034 | 0.006 | 2.38E-09 | 0.0026 | 32.11 |
| rs1737329 | G | C | 0.742 | 0.017 | 0.003 | 5.08E-09 | 0.0026 | 32.11 |
| rs6948707 | G | T | 0.419 | 0.024 | 0.003 | 4.24E-21 | 0.0052 | 64.00 |
| rs13237637 | C | G | 0.485 | -0.024 | 0.003 | 1.54E-20 | 0.0052 | 64.00 |
| rs7809303 | A | G | 0.325 | -0.021 | 0.003 | 3.48E-15 | 0.0040 | 49.00 |
| rs7802996 | T | C | 0.166 | -0.021 | 0.003 | 1.06E-09 | 0.0040 | 49.00 |
| rs1030015 | T | G | 0.5196 | 0.014 | 0.003 | 2.15E-08 | 0.0018 | 21.78 |
| rs4727189 | C | T | 0.344 | 0.015 | 0.003 | 3.00E-08 | 0.0020 | 25.00 |
| rs76841737 | G | C | 0.103 | -0.023 | 0.004 | 3.26E-08 | 0.0027 | 33.06 |
| rs11768481 | A | C | 0.34 | -0.019 | 0.003 | 5.23E-12 | 0.0033 | 40.11 |
| rs1799068 | T | G | 0.379 | 0.017 | 0.003 | 2.59E-10 | 0.0026 | 32.11 |
| rs13437771 | G | A | 0.155 | -0.027 | 0.004 | 1.39E-14 | 0.0037 | 45.56 |
| rs11766326 | C | T | 0.506 | -0.018 | 0.003 | 1.79E-11 | 0.0029 | 36.00 |
| rs6968380 | A | G | 0.681 | -0.023 | 0.003 | 1.05E-17 | 0.0048 | 58.78 |
| rs10233018 | G | A | 0.516 | 0.025 | 0.003 | 4.77E-22 | 0.0056 | 69.44 |
| rs10953957 | A | G | 0.386 | 0.014 | 0.003 | 3.66E-08 | 0.0018 | 21.78 |
| rs77283305 | A | G | 0.3058 | -0.015 | 0.003 | 3.91E-08 | 0.0020 | 25.00 |
| rs10279261 | A | G | 0.618 | -0.019 | 0.003 | 6.05E-13 | 0.0033 | 40.11 |
| rs4326350 | G | C | 0.493 | -0.018 | 0.003 | 5.16E-12 | 0.0029 | 36.00 |
| rs11783093 | T | C | 0.158 | -0.047 | 0.003 | 2.07E-41 | 0.0199 | 245.44 |
| rs7836565 | T | C | 0.718 | -0.016 | 0.003 | 4.36E-08 | 0.0023 | 28.44 |
| rs13261666 | T | G | 0.517 | -0.02 | 0.003 | 4.36E-15 | 0.0036 | 44.44 |
| rs3850736 | G | C | 0.474 | 0.019 | 0.003 | 6.43E-14 | 0.0033 | 40.11 |
| rs2063976 | T | C | 0.665 | -0.02 | 0.003 | 7.45E-14 | 0.0036 | 44.44 |
| rs6986430 | C | T | 0.2224 | -0.024 | 0.003 | 1.99E-15 | 0.0052 | 64.00 |
| rs9987376 | G | T | 0.5743 | -0.02 | 0.003 | 2.01E-15 | 0.0036 | 44.44 |
| rs290601 | T | C | 0.274 | 0.016 | 0.003 | 1.14E-08 | 0.0023 | 28.44 |
| rs3847244 | T | C | 0.47 | 0.019 | 0.003 | 2.60E-13 | 0.0033 | 40.11 |
| rs11791671 | T | C | 0.0673 | 0.028 | 0.005 | 4.24E-08 | 0.0025 | 31.36 |
| rs7024924 | C | T | 0.174 | 0.019 | 0.003 | 1.90E-08 | 0.0033 | 40.11 |
| rs1931431 | C | G | 0.478 | 0.018 | 0.003 | 8.56E-13 | 0.0029 | 36.00 |
| rs7867822 | G | A | 0.673 | -0.015 | 0.003 | 2.76E-08 | 0.0020 | 25.00 |
| rs10966092 | C | T | 0.267 | -0.02 | 0.003 | 1.12E-12 | 0.0036 | 44.44 |
| rs10969352 | A | T | 0.5 | 0.014 | 0.003 | 1.82E-08 | 0.0018 | 21.78 |
| rs4877285 | A | G | 0.6682 | -0.018 | 0.003 | 2.10E-11 | 0.0029 | 36.00 |
| rs1930371 | T | C | 0.241 | -0.017 | 0.003 | 7.09E-09 | 0.0026 | 32.11 |
| rs2378662 | A | G | 0.541 | 0.015 | 0.003 | 2.67E-09 | 0.0020 | 25.00 |
| rs1927901 | C | T | 0.553 | -0.014 | 0.003 | 3.10E-08 | 0.0018 | 21.78 |
| rs4837631 | T | C | 0.446 | -0.015 | 0.003 | 2.03E-09 | 0.0020 | 25.00 |
| rs1759433 | A | G | 0.48 | 0.015 | 0.003 | 1.69E-09 | 0.0020 | 25.00 |
| rs34553878 | G | A | 0.111 | 0.025 | 0.004 | 1.17E-09 | 0.0032 | 39.06 |
| rs7026534 | G | T | 0.7038 | -0.017 | 0.003 | 2.68E-09 | 0.0026 | 32.11 |
| rs10858334 | G | C | 0.14 | 0.023 | 0.004 | 1.18E-09 | 0.0027 | 33.06 |
| rs10905461 | C | T | 0.748 | -0.016 | 0.003 | 2.36E-08 | 0.0023 | 28.44 |
| rs7920501 | A | T | 0.465 | -0.016 | 0.003 | 1.25E-09 | 0.0023 | 28.44 |
| rs1291821 | G | A | 0.534 | 0.014 | 0.003 | 1.39E-08 | 0.0018 | 21.78 |
| rs11258417 | T | C | 0.391 | -0.015 | 0.003 | 2.71E-08 | 0.0020 | 25.00 |
| rs7072776 | G | A | 0.712 | -0.022 | 0.003 | 5.66E-15 | 0.0044 | 53.78 |
| rs2796793 | A | G | 0.452 | 0.014 | 0.003 | 1.55E-08 | 0.0018 | 21.78 |
| rs1733760 | C | T | 0.51 | 0.015 | 0.003 | 6.70E-09 | 0.0020 | 25.00 |
| rs7921378 | C | G | 0.482 | -0.023 | 0.003 | 6.10E-20 | 0.0048 | 58.78 |
| rs11594623 | C | T | 0.2342 | 0.027 | 0.003 | 7.45E-20 | 0.0066 | 81.00 |
| rs28408682 | G | A | 0.6 | 0.017 | 0.003 | 1.41E-10 | 0.0026 | 32.11 |
| rs12244388 | A | G | 0.35 | 0.026 | 0.003 | 4.31E-22 | 0.0061 | 75.11 |
| rs11192347 | A | G | 0.104 | -0.026 | 0.004 | 6.15E-10 | 0.0034 | 42.25 |
| rs10885480 | C | T | 0.284 | -0.019 | 0.003 | 3.83E-11 | 0.0033 | 40.11 |
| rs4752018 | A | C | 0.231 | 0.019 | 0.003 | 4.42E-10 | 0.0033 | 40.11 |
| rs9423279 | G | C | 0.645 | -0.019 | 0.003 | 3.06E-12 | 0.0033 | 40.11 |
| rs6265 | T | C | 0.188 | -0.029 | 0.003 | 2.81E-19 | 0.0076 | 93.44 |
| rs62618693 | T | C | 0.0428 | -0.035 | 0.006 | 2.09E-08 | 0.0028 | 34.03 |
| rs2939756 | A | G | 0.48 | -0.016 | 0.003 | 7.45E-10 | 0.0023 | 28.44 |
| rs1381775 | C | T | 0.712 | -0.016 | 0.003 | 2.79E-08 | 0.0023 | 28.44 |
| rs2959084 | A | G | 0.7047 | 0.017 | 0.003 | 9.82E-10 | 0.0026 | 32.11 |
| rs3740977 | C | T | 0.167 | 0.019 | 0.003 | 1.17E-08 | 0.0033 | 40.11 |
| rs61886926 | T | C | 0.384 | -0.018 | 0.003 | 7.30E-12 | 0.0029 | 36.00 |
| rs61884449 | T | C | 0.1492 | 0.02 | 0.004 | 2.32E-08 | 0.0020 | 25.00 |
| rs644740 | T | C | 0.457 | -0.014 | 0.003 | 3.67E-08 | 0.0018 | 21.78 |
| rs7943721 | A | G | 0.829 | -0.021 | 0.003 | 3.58E-10 | 0.0040 | 49.00 |
| rs7929518 | G | A | 0.773 | 0.019 | 0.003 | 2.55E-10 | 0.0033 | 40.11 |
| rs586699 | A | G | 0.543 | -0.015 | 0.003 | 7.29E-09 | 0.0020 | 25.00 |
| rs76460663 | G | C | 0.0411 | -0.042 | 0.006 | 4.15E-11 | 0.0040 | 49.00 |
| rs2155646 | C | T | 0.4 | 0.038 | 0.003 | 9.44E-48 | 0.0130 | 160.44 |
| rs1713676 | G | A | 0.5225 | -0.017 | 0.003 | 5.38E-11 | 0.0026 | 32.11 |
| rs238896 | A | G | 0.49 | -0.017 | 0.003 | 3.65E-11 | 0.0026 | 32.11 |
| rs540860 | G | A | 0.543 | 0.018 | 0.003 | 5.75E-12 | 0.0029 | 36.00 |
| rs1106363 | T | C | 0.3446 | 0.017 | 0.003 | 9.20E-11 | 0.0026 | 32.11 |
| rs2010921 | A | G | 0.311 | 0.017 | 0.003 | 2.47E-10 | 0.0026 | 32.11 |
| rs11057005 | G | A | 0.441 | -0.016 | 0.003 | 9.12E-10 | 0.0023 | 28.44 |
| rs13906 | T | C | 0.109 | -0.025 | 0.004 | 1.98E-09 | 0.0032 | 39.06 |
| rs4759229 | G | A | 0.656 | 0.016 | 0.003 | 6.53E-09 | 0.0023 | 28.44 |
| rs7969559 | G | A | 0.713 | -0.017 | 0.003 | 1.53E-09 | 0.0026 | 32.11 |
| rs7134009 | C | T | 0.287 | -0.016 | 0.003 | 4.30E-08 | 0.0023 | 28.44 |
| rs77215829 | C | A | 0.131 | -0.024 | 0.004 | 2.02E-10 | 0.0029 | 36.00 |
| rs1109480 | A | G | 0.384 | -0.017 | 0.003 | 1.84E-10 | 0.0026 | 32.11 |
| rs11611651 | A | G | 0.0868 | 0.027 | 0.005 | 2.05E-09 | 0.0024 | 29.16 |
| rs17197663 | A | G | 0.125 | -0.022 | 0.004 | 2.06E-08 | 0.0025 | 30.25 |
| rs4264267 | T | C | 0.527 | 0.015 | 0.003 | 6.82E-09 | 0.0020 | 25.00 |
| rs61959481 | A | G | 0.21 | -0.02 | 0.003 | 7.95E-11 | 0.0036 | 44.44 |
| rs9538162 | C | T | 0.4159 | 0.017 | 0.003 | 1.76E-11 | 0.0026 | 32.11 |
| rs55786907 | G | A | 0.1625 | 0.019 | 0.003 | 1.84E-08 | 0.0033 | 40.11 |
| rs4886207 | C | T | 0.637 | -0.016 | 0.003 | 8.78E-10 | 0.0023 | 28.44 |
| rs9540731 | T | C | 0.509 | -0.018 | 0.003 | 3.42E-12 | 0.0029 | 36.00 |
| rs9545155 | C | T | 0.478 | -0.016 | 0.003 | 3.04E-10 | 0.0023 | 28.44 |
| rs1772572 | A | C | 0.3241 | -0.017 | 0.003 | 5.62E-10 | 0.0026 | 32.11 |
| rs75674569 | A | G | 0.0997 | -0.025 | 0.004 | 2.58E-09 | 0.0032 | 39.06 |
| rs7333559 | A | G | 0.783 | -0.023 | 0.003 | 5.94E-14 | 0.0048 | 58.78 |
| rs1108130 | A | T | 0.212 | 0.024 | 0.003 | 1.57E-14 | 0.0052 | 64.00 |
| rs12855717 | T | C | 0.538 | 0.016 | 0.003 | 1.22E-09 | 0.0023 | 28.44 |
| rs12878369 | A | C | 0.4148 | 0.017 | 0.003 | 1.60E-11 | 0.0026 | 32.11 |
| rs9323328 | G | A | 0.537 | -0.014 | 0.003 | 2.55E-08 | 0.0018 | 21.78 |
| rs1811739 | A | G | 0.248 | 0.018 | 0.003 | 5.97E-10 | 0.0029 | 36.00 |
| rs8005334 | G | T | 0.36 | 0.017 | 0.003 | 3.44E-10 | 0.0026 | 32.11 |
| rs34940743 | G | A | 0.346 | 0.016 | 0.003 | 2.80E-09 | 0.0023 | 28.44 |
| rs2925128 | T | C | 0.3852 | 0.017 | 0.003 | 3.67E-10 | 0.0026 | 32.11 |
| rs1381287 | T | C | 0.467 | 0.018 | 0.003 | 1.81E-12 | 0.0029 | 36.00 |
| rs55913542 | T | G | 0.175 | 0.019 | 0.003 | 3.25E-08 | 0.0033 | 40.11 |
| rs1435672 | C | T | 0.56 | 0.014 | 0.003 | 3.82E-08 | 0.0018 | 21.78 |
| rs281296 | A | G | 0.357 | 0.025 | 0.003 | 1.59E-20 | 0.0056 | 69.44 |
| rs56902655 | G | T | 0.136 | -0.022 | 0.004 | 4.09E-09 | 0.0025 | 30.25 |
| rs2289791 | T | G | 0.247 | -0.018 | 0.003 | 2.01E-09 | 0.0029 | 36.00 |
| rs60833441 | G | A | 0.461 | -0.014 | 0.003 | 2.28E-08 | 0.0018 | 21.78 |
| rs62007780 | T | G | 0.416 | -0.016 | 0.003 | 7.48E-10 | 0.0023 | 28.44 |
| rs4310804 | G | C | 0.247 | -0.018 | 0.003 | 7.55E-10 | 0.0029 | 36.00 |
| rs8027457 | C | T | 0.511 | 0.015 | 0.003 | 1.88E-09 | 0.0020 | 25.00 |
| rs1139897 | A | G | 0.23 | -0.024 | 0.003 | 1.77E-15 | 0.0052 | 64.00 |
| rs11076962 | C | T | 0.279 | 0.018 | 0.003 | 1.20E-10 | 0.0029 | 36.00 |
| rs7192140 | C | T | 0.498 | -0.017 | 0.003 | 3.40E-11 | 0.0026 | 32.11 |
| rs9922607 | T | C | 0.2 | -0.022 | 0.003 | 3.42E-12 | 0.0044 | 53.78 |
| rs9941217 | G | C | 0.3522 | -0.019 | 0.003 | 3.50E-12 | 0.0033 | 40.11 |
| rs7188873 | G | A | 0.613 | 0.02 | 0.003 | 8.46E-15 | 0.0036 | 44.44 |
| rs6497840 | A | G | 0.707 | 0.023 | 0.003 | 2.01E-15 | 0.0048 | 58.78 |
| rs4785187 | A | G | 0.223 | 0.02 | 0.003 | 6.55E-11 | 0.0036 | 44.44 |
| rs8050598 | T | C | 0.2541 | 0.019 | 0.003 | 1.76E-10 | 0.0033 | 40.11 |
| rs12918191 | G | A | 0.243 | -0.02 | 0.003 | 3.14E-11 | 0.0036 | 44.44 |
| rs9302604 | G | A | 0.435 | 0.019 | 0.003 | 3.29E-13 | 0.0033 | 40.11 |
| rs62052916 | T | A | 0.0701 | -0.032 | 0.005 | 1.62E-10 | 0.0033 | 40.96 |
| rs4788676 | C | T | 0.2285 | -0.018 | 0.003 | 4.92E-09 | 0.0029 | 36.00 |
| rs117657830 | G | A | 0.0417 | -0.038 | 0.006 | 3.18E-09 | 0.0033 | 40.11 |
| rs1050847 | T | C | 0.559 | -0.015 | 0.003 | 7.37E-09 | 0.0020 | 25.00 |
| rs11642231 | A | G | 0.369 | -0.016 | 0.003 | 3.44E-09 | 0.0023 | 28.44 |
| rs4790874 | T | C | 0.532 | 0.017 | 0.003 | 8.43E-12 | 0.0026 | 32.11 |
| rs28441558 | C | T | 0.0563 | -0.036 | 0.006 | 1.24E-10 | 0.0029 | 36.00 |
| rs11651955 | A | G | 0.499 | -0.014 | 0.003 | 3.74E-08 | 0.0018 | 21.78 |
| rs67777803 | T | G | 0.172 | -0.025 | 0.003 | 3.18E-13 | 0.0056 | 69.44 |
| rs2344976 | C | T | 0.612 | -0.015 | 0.003 | 7.98E-09 | 0.0020 | 25.00 |
| rs17692129 | T | C | 0.331 | 0.02 | 0.003 | 4.57E-13 | 0.0036 | 44.44 |
| rs75919030 | C | T | 0.267 | -0.021 | 0.003 | 3.35E-13 | 0.0040 | 49.00 |
| rs2587507 | C | T | 0.502 | -0.015 | 0.003 | 8.69E-09 | 0.0020 | 25.00 |
| rs34342129 | C | T | 0.509 | -0.014 | 0.003 | 2.13E-08 | 0.0018 | 21.78 |
| rs4476253 | A | G | 0.24 | -0.018 | 0.003 | 5.78E-10 | 0.0029 | 36.00 |
| rs7505855 | T | C | 0.586 | -0.017 | 0.003 | 5.31E-11 | 0.0026 | 32.11 |
| rs8096225 | C | A | 0.703 | 0.016 | 0.003 | 2.63E-08 | 0.0023 | 28.44 |
| rs67050670 | G | A | 0.229 | -0.02 | 0.003 | 2.34E-11 | 0.0036 | 44.44 |
| rs2359180 | G | A | 0.369 | -0.014 | 0.003 | 4.98E-08 | 0.0018 | 21.78 |
| rs72898831 | G | A | 0.155 | -0.024 | 0.004 | 4.14E-12 | 0.0029 | 36.00 |
| rs1373178 | G | T | 0.588 | -0.02 | 0.003 | 4.16E-15 | 0.0036 | 44.44 |
| rs62098013 | A | G | 0.3653 | 0.018 | 0.003 | 2.24E-11 | 0.0029 | 36.00 |
| rs72938304 | A | G | 0.113 | -0.027 | 0.004 | 1.36E-11 | 0.0037 | 45.56 |
| rs11872397 | A | G | 0.253 | -0.017 | 0.003 | 5.20E-09 | 0.0026 | 32.11 |
| rs71367544 | T | C | 0.203 | 0.021 | 0.003 | 8.54E-11 | 0.0040 | 49.00 |
| rs76608582 | A | C | 0.0489 | -0.035 | 0.006 | 4.88E-09 | 0.0028 | 34.03 |
| rs10853981 | A | G | 0.3304 | 0.015 | 0.003 | 4.88E-08 | 0.0020 | 25.00 |
| rs113230003 | A | G | 0.255 | -0.019 | 0.003 | 1.05E-10 | 0.0033 | 40.11 |
| rs8103660 | C | T | 0.3544 | 0.016 | 0.003 | 3.03E-09 | 0.0023 | 28.44 |
| rs117734003 | C | G | 0.0673 | 0.03 | 0.005 | 2.57E-09 | 0.0029 | 36.00 |
| rs1126757 | T | C | 0.473 | 0.014 | 0.003 | 2.92E-08 | 0.0018 | 21.78 |
| rs6050446 | G | A | 0.971 | 0.054 | 0.008 | 8.80E-13 | 0.0037 | 45.56 |
| rs6073075 | A | T | 0.824 | -0.019 | 0.003 | 2.44E-08 | 0.0033 | 40.11 |
| rs910912 | C | T | 0.739 | -0.017 | 0.003 | 7.82E-09 | 0.0026 | 32.11 |
| rs6011779 | T | C | 0.806 | -0.019 | 0.003 | 2.83E-09 | 0.0033 | 40.11 |
| rs3810496 | C | T | 0.6194 | 0.016 | 0.003 | 1.54E-09 | 0.0023 | 28.44 |
| rs4818005 | A | G | 0.581 | -0.02 | 0.003 | 1.09E-14 | 0.0036 | 44.44 |
| rs4822102 | T | C | 0.618 | -0.017 | 0.003 | 2.78E-10 | 0.0026 | 32.11 |
| rs9627272 | C | G | 0.407 | -0.015 | 0.003 | 2.42E-09 | 0.0020 | 25.00 |

SNP, single nucleotide polymorphism; EA, effect allele; OA, other allele; EAF, effect allele frequency. F-statistic quantified the strength of each selected SNP using the formula R^2^ × (N − 2) / (1 − R^2^). R^2^ = [2 × BETA^2^ × EAF × (1 − EAF)] / [2 × BETA^2^ × EAF × (1 – EAF) + 2 × SE^2^ × N × EAF × (1 − EAF)].

**Supplementary table 3.** SNPs associated with lifetime smoking index at the genome-wide level of significance (*P* < 5 x 10^-8^) and clumped for independence at kb = 10,000 and *r^2^* = 0.01

| **SNP** | **EA** | **OA** | **EAF** | **BETA** | **SE** | ***P*-value** | **R^2^ %** | **F** |
| --- | --- | --- | --- | --- | --- | --- | --- | --- |
| rs8042849 | C | T | 0.342 | 0.019 | 0.0015 | 1.80E-39 | 0.0373 | 172.80 |
| rs113382419 | C | A | 0.889 | -0.028 | 0.0022 | 3.00E-37 | 0.0351 | 162.66 |
| rs6011779 | C | T | 0.191 | 0.019 | 0.0018 | 2.30E-27 | 0.0254 | 117.47 |
| rs9919670 | G | A | 0.612 | -0.015 | 0.0014 | 7.60E-27 | 0.0249 | 115.06 |
| rs2890772 | G | T | 0.413 | -0.014 | 0.0014 | 2.10E-22 | 0.0205 | 94.82 |
| rs35175834 | G | A | 0.788 | -0.016 | 0.0017 | 4.60E-22 | 0.0202 | 93.27 |
| rs12244388 | G | A | 0.661 | -0.013 | 0.0015 | 1.40E-19 | 0.0177 | 81.90 |
| rs11783093 | C | T | 0.839 | 0.016 | 0.0019 | 1.20E-16 | 0.0148 | 68.62 |
| rs11210229 | A | G | 0.384 | 0.012 | 0.0014 | 2.00E-16 | 0.0146 | 67.65 |
| rs62155874 | A | G | 0.873 | -0.017 | 0.0021 | 5.20E-16 | 0.0142 | 65.73 |
| rs10226228 | A | G | 0.630 | -0.011 | 0.0014 | 2.00E-15 | 0.0136 | 63.04 |
| rs6119897 | G | A | 0.762 | -0.013 | 0.0016 | 3.60E-15 | 0.0134 | 61.93 |
| rs2867112 | T | G | 0.835 | 0.015 | 0.0019 | 4.80E-15 | 0.0133 | 61.34 |
| rs986391 | G | A | 0.367 | 0.011 | 0.0014 | 9.40E-15 | 0.0130 | 60.01 |
| rs3742365 | T | C | 0.595 | -0.011 | 0.0014 | 2.50E-14 | 0.0126 | 58.10 |
| rs2401924 | G | C | 0.502 | 0.011 | 0.0014 | 2.70E-14 | 0.0125 | 57.92 |
| rs7807019 | A | G | 0.540 | -0.010 | 0.0014 | 6.70E-14 | 0.0121 | 56.16 |
| rs549845 | G | A | 0.301 | 0.011 | 0.0015 | 8.30E-14 | 0.0120 | 55.72 |
| rs10922907 | A | T | 0.451 | 0.010 | 0.0014 | 3.00E-13 | 0.0115 | 53.20 |
| rs7569203 | A | C | 0.689 | -0.011 | 0.0015 | 7.40E-13 | 0.0111 | 51.44 |
| rs17309874 | G | A | 0.740 | -0.011 | 0.0016 | 9.70E-13 | 0.0110 | 50.91 |
| rs6778080 | T | C | 0.267 | 0.011 | 0.0016 | 1.30E-12 | 0.0109 | 50.36 |
| rs8042134 | T | G | 0.541 | -0.010 | 0.0014 | 1.30E-12 | 0.0109 | 50.34 |
| rs17576594 | G | A | 0.724 | 0.011 | 0.0016 | 1.70E-12 | 0.0108 | 49.81 |
| rs7766610 | C | A | 0.183 | 0.013 | 0.0018 | 2.20E-12 | 0.0106 | 49.27 |
| rs1922018 | C | T | 0.364 | 0.010 | 0.0014 | 3.00E-12 | 0.0105 | 48.70 |
| rs7553348 | G | A | 0.438 | 0.010 | 0.0014 | 5.20E-12 | 0.0103 | 47.60 |
| rs7528604 | G | A | 0.566 | 0.010 | 0.0014 | 5.70E-12 | 0.0103 | 47.44 |
| rs329120 | C | T | 0.581 | 0.010 | 0.0014 | 6.30E-12 | 0.0102 | 47.24 |
| rs12623702 | A | G | 0.613 | -0.010 | 0.0014 | 7.70E-12 | 0.0101 | 46.84 |
| rs6935954 | A | G | 0.421 | 0.010 | 0.0014 | 8.20E-12 | 0.0101 | 46.71 |
| rs4671357 | T | C | 0.519 | -0.009 | 0.0014 | 1.10E-11 | 0.0100 | 46.15 |
| rs3896224 | A | G | 0.585 | 0.010 | 0.0014 | 1.10E-11 | 0.0100 | 46.10 |
| rs326341 | G | A | 0.525 | 0.009 | 0.0014 | 1.20E-11 | 0.0099 | 45.91 |
| rs4391802 | A | G | 0.707 | 0.010 | 0.0015 | 1.40E-11 | 0.0099 | 45.61 |
| rs72678864 | G | A | 0.829 | 0.012 | 0.0018 | 1.60E-11 | 0.0098 | 45.35 |
| rs112282219 | G | A | 0.959 | -0.023 | 0.0035 | 3.80E-11 | 0.0094 | 43.71 |
| rs10879871 | T | G | 0.343 | -0.010 | 0.0015 | 5.00E-11 | 0.0093 | 43.17 |
| rs889398 | C | T | 0.588 | 0.009 | 0.0014 | 6.30E-11 | 0.0092 | 42.73 |
| rs4473348 | A | T | 0.250 | -0.010 | 0.0016 | 6.40E-11 | 0.0092 | 42.68 |
| rs1221148 | C | G | 0.587 | 0.009 | 0.0014 | 7.30E-11 | 0.0092 | 42.42 |
| rs317021 | T | A | 0.814 | -0.012 | 0.0018 | 1.10E-10 | 0.0090 | 41.72 |
| rs8614 | C | A | 0.817 | -0.011 | 0.0018 | 1.80E-10 | 0.0088 | 40.69 |
| rs11255908 | T | G | 0.743 | -0.010 | 0.0016 | 2.30E-10 | 0.0087 | 40.22 |
| rs13153393 | A | G | 0.884 | -0.014 | 0.0022 | 2.50E-10 | 0.0086 | 40.00 |
| rs7333559 | G | A | 0.212 | 0.011 | 0.0017 | 3.20E-10 | 0.0086 | 39.58 |
| rs76608582 | C | A | 0.953 | 0.022 | 0.0034 | 3.20E-10 | 0.0085 | 39.54 |
| rs421983 | T | C | 0.519 | 0.009 | 0.0014 | 3.30E-10 | 0.0085 | 39.49 |
| rs4543592 | T | C | 0.520 | -0.009 | 0.0014 | 4.50E-10 | 0.0084 | 38.89 |
| rs11948770 | T | C | 0.768 | -0.010 | 0.0016 | 4.90E-10 | 0.0084 | 38.71 |
| rs7039819 | G | A | 0.427 | 0.009 | 0.0014 | 5.10E-10 | 0.0084 | 38.65 |
| rs10282292 | C | T | 0.362 | 0.009 | 0.0014 | 5.90E-10 | 0.0083 | 38.37 |
| rs2838834 | C | T | 0.699 | -0.009 | 0.0015 | 6.30E-10 | 0.0083 | 38.22 |
| rs624833 | T | G | 0.695 | 0.009 | 0.0015 | 6.60E-10 | 0.0082 | 38.14 |
| rs3811038 | T | C | 0.724 | -0.010 | 0.0016 | 8.90E-10 | 0.0081 | 37.56 |
| rs359243 | T | C | 0.393 | -0.009 | 0.0014 | 9.50E-10 | 0.0081 | 37.42 |
| rs11768481 | C | A | 0.666 | 0.009 | 0.0015 | 9.90E-10 | 0.0081 | 37.35 |
| rs6779302 | G | T | 0.633 | -0.009 | 0.0014 | 1.20E-09 | 0.0080 | 36.98 |
| rs35169606 | T | G | 0.612 | 0.009 | 0.0014 | 1.20E-09 | 0.0080 | 36.95 |
| rs67596067 | G | A | 0.649 | -0.009 | 0.0015 | 1.20E-09 | 0.0080 | 37.04 |
| rs2675638 | G | A | 0.581 | 0.008 | 0.0014 | 1.30E-09 | 0.0080 | 36.84 |
| rs75742406 | G | A | 0.739 | 0.010 | 0.0016 | 1.30E-09 | 0.0080 | 36.84 |
| rs71367545 | G | A | 0.791 | -0.010 | 0.0017 | 1.40E-09 | 0.0079 | 36.69 |
| rs71627581 | G | A | 0.889 | 0.013 | 0.0022 | 1.60E-09 | 0.0079 | 36.35 |
| rs13016665 | C | A | 0.577 | -0.008 | 0.0014 | 1.80E-09 | 0.0078 | 36.14 |
| rs369230 | G | T | 0.308 | -0.009 | 0.0015 | 1.80E-09 | 0.0078 | 36.18 |
| rs3769949 | T | A | 0.528 | -0.008 | 0.0014 | 2.50E-09 | 0.0077 | 35.51 |
| rs7155595 | A | C | 0.674 | -0.009 | 0.0015 | 2.50E-09 | 0.0077 | 35.53 |
| rs860326 | C | T | 0.428 | 0.008 | 0.0014 | 2.70E-09 | 0.0076 | 35.35 |
| rs12202536 | A | G | 0.513 | -0.008 | 0.0014 | 2.80E-09 | 0.0076 | 35.29 |
| rs4814873 | C | T | 0.767 | 0.010 | 0.0016 | 2.90E-09 | 0.0076 | 35.23 |
| rs147412694 | G | A | 0.850 | -0.012 | 0.0019 | 2.90E-09 | 0.0076 | 35.27 |
| rs9842947 | C | T | 0.326 | -0.009 | 0.0015 | 3.10E-09 | 0.0076 | 35.09 |
| rs2894808 | T | A | 0.922 | -0.015 | 0.0026 | 3.50E-09 | 0.0075 | 34.86 |
| rs12708665 | A | G | 0.285 | -0.009 | 0.0015 | 3.50E-09 | 0.0075 | 34.87 |
| rs202645 | A | G | 0.203 | -0.010 | 0.0017 | 3.90E-09 | 0.0075 | 34.69 |
| rs62098013 | G | A | 0.640 | -0.009 | 0.0015 | 4.10E-09 | 0.0075 | 34.58 |
| rs4957528 | A | C | 0.208 | -0.010 | 0.0017 | 4.20E-09 | 0.0075 | 34.55 |
| rs1246265 | T | C | 0.305 | -0.009 | 0.0015 | 4.20E-09 | 0.0075 | 34.51 |
| rs6598539 | T | C | 0.489 | -0.008 | 0.0014 | 4.50E-09 | 0.0074 | 34.41 |
| rs13009008 | A | G | 0.328 | 0.009 | 0.0015 | 4.60E-09 | 0.0074 | 34.33 |
| rs17553262 | A | C | 0.885 | -0.013 | 0.0022 | 5.30E-09 | 0.0074 | 34.07 |
| rs7297175 | T | C | 0.431 | -0.008 | 0.0014 | 6.60E-09 | 0.0073 | 33.65 |
| rs6962772 | A | G | 0.846 | 0.011 | 0.0019 | 7.80E-09 | 0.0072 | 33.33 |
| rs12481282 | G | C | 0.722 | -0.009 | 0.0015 | 7.80E-09 | 0.0072 | 33.32 |
| rs35343344 | C | A | 0.733 | 0.009 | 0.0016 | 8.80E-09 | 0.0072 | 33.10 |
| rs6562474 | C | G | 0.651 | 0.008 | 0.0015 | 1.00E-08 | 0.0071 | 32.81 |
| rs2062882 | G | A | 0.587 | -0.008 | 0.0014 | 1.10E-08 | 0.0070 | 32.62 |
| rs9435340 | T | A | 0.344 | 0.008 | 0.0015 | 1.20E-08 | 0.0070 | 32.50 |
| rs1050847 | C | T | 0.426 | 0.008 | 0.0014 | 1.40E-08 | 0.0070 | 32.18 |
| rs73220544 | A | C | 0.842 | -0.011 | 0.0019 | 1.50E-08 | 0.0069 | 32.00 |
| rs4571506 | C | T | 0.540 | 0.008 | 0.0014 | 1.50E-08 | 0.0069 | 32.03 |
| rs732083 | G | A | 0.333 | 0.008 | 0.0015 | 1.50E-08 | 0.0069 | 32.12 |
| rs6741228 | T | C | 0.433 | 0.008 | 0.0014 | 1.60E-08 | 0.0069 | 31.94 |
| rs4949465 | T | C | 0.870 | -0.012 | 0.0021 | 1.70E-08 | 0.0069 | 31.80 |
| rs62175972 | T | C | 0.966 | 0.022 | 0.0039 | 1.70E-08 | 0.0069 | 31.79 |
| rs136233 | A | G | 0.809 | -0.010 | 0.0018 | 1.80E-08 | 0.0068 | 31.67 |
| rs12831617 | C | T | 0.764 | -0.009 | 0.0016 | 1.90E-08 | 0.0068 | 31.63 |
| rs11861214 | G | T | 0.784 | 0.009 | 0.0017 | 2.00E-08 | 0.0068 | 31.48 |
| rs10918701 | G | A | 0.372 | 0.008 | 0.0014 | 2.10E-08 | 0.0068 | 31.40 |
| rs10823968 | A | T | 0.633 | 0.008 | 0.0015 | 2.10E-08 | 0.0068 | 31.37 |
| rs74086911 | G | A | 0.925 | 0.015 | 0.0026 | 2.10E-08 | 0.0068 | 31.39 |
| rs4731925 | C | T | 0.316 | -0.008 | 0.0015 | 2.60E-08 | 0.0067 | 30.96 |
| rs60952428 | T | C | 0.909 | 0.013 | 0.0024 | 3.00E-08 | 0.0066 | 30.73 |
| rs12967855 | A | G | 0.331 | 0.008 | 0.0015 | 3.10E-08 | 0.0066 | 30.64 |
| rs2254710 | C | A | 0.236 | 0.009 | 0.0016 | 3.50E-08 | 0.0066 | 30.43 |
| rs57611503 | G | A | 0.485 | 0.008 | 0.0014 | 4.00E-08 | 0.0065 | 30.15 |
| rs61796681 | A | T | 0.912 | -0.013 | 0.0024 | 4.20E-08 | 0.0065 | 30.05 |
| rs2080870 | A | T | 0.258 | 0.009 | 0.0016 | 4.90E-08 | 0.0064 | 29.76 |
| rs6692614 | C | G | 0.421 | -0.008 | 0.0014 | 2.80E-08 | 0.0067 | 30.85 |
| rs6665841 | C | T | 0.228 | 0.011 | 0.0017 | 1.40E-10 | 0.0089 | 41.12 |
| rs1931262 | T | C | 0.509 | -0.008 | 0.0014 | 3.50E-08 | 0.0066 | 30.41 |
| rs11801576 | T | C | 0.755 | -0.010 | 0.0016 | 2.30E-10 | 0.0087 | 40.18 |
| rs62107261 | T | C | 0.952 | 0.021 | 0.0032 | 8.20E-11 | 0.0091 | 42.21 |
| rs75120545 | C | T | 0.970 | 0.027 | 0.0043 | 5.20E-10 | 0.0083 | 38.61 |
| rs11123962 | T | G | 0.554 | -0.009 | 0.0014 | 1.60E-10 | 0.0088 | 40.90 |
| rs7588808 | T | C | 0.626 | -0.010 | 0.0014 | 3.40E-11 | 0.0095 | 43.91 |
| rs12693975 | G | A | 0.186 | -0.010 | 0.0018 | 4.10E-09 | 0.0075 | 34.57 |
| rs812887 | A | G | 0.414 | 0.008 | 0.0014 | 5.00E-09 | 0.0074 | 34.21 |
| rs78526269 | T | C | 0.781 | 0.010 | 0.0017 | 1.50E-09 | 0.0079 | 36.58 |
| rs62261746 | C | G | 0.697 | 0.008 | 0.0015 | 4.20E-08 | 0.0065 | 30.05 |
| rs17314832 | C | T | 0.642 | 0.009 | 0.0014 | 3.70E-09 | 0.0075 | 34.77 |
| rs7687139 | G | C | 0.592 | 0.008 | 0.0014 | 4.70E-08 | 0.0065 | 29.85 |
| rs13106038 | G | A | 0.676 | 0.009 | 0.0015 | 2.20E-09 | 0.0077 | 35.83 |
| rs35120974 | A | G | 0.583 | -0.008 | 0.0014 | 2.00E-09 | 0.0078 | 35.93 |
| rs263892 | G | A | 0.611 | 0.008 | 0.0014 | 1.30E-08 | 0.0070 | 32.34 |
| rs13178466 | C | T | 0.406 | 0.008 | 0.0014 | 4.80E-08 | 0.0064 | 29.80 |
| rs11134664 | G | A | 0.313 | -0.009 | 0.0015 | 5.00E-10 | 0.0084 | 38.67 |
| rs9277988 | T | C | 0.803 | -0.010 | 0.0017 | 2.00E-08 | 0.0068 | 31.53 |
| rs10945143 | T | C | 0.744 | -0.009 | 0.0016 | 2.70E-08 | 0.0067 | 30.88 |
| rs10228494 | C | G | 0.427 | -0.008 | 0.0014 | 3.20E-09 | 0.0076 | 35.04 |
| rs2880449 | C | T | 0.498 | -0.008 | 0.0014 | 2.80E-08 | 0.0067 | 30.83 |
| rs71532325 | C | T | 0.683 | 0.008 | 0.0015 | 2.30E-08 | 0.0068 | 31.24 |
| rs10759934 | A | T | 0.514 | 0.008 | 0.0014 | 6.10E-09 | 0.0073 | 33.79 |
| rs10819050 | G | T | 0.588 | -0.011 | 0.0014 | 2.20E-15 | 0.0136 | 62.90 |
| rs3025383 | T | C | 0.817 | 0.013 | 0.0018 | 2.20E-12 | 0.0107 | 49.33 |
| rs2244524 | C | T | 0.554 | 0.009 | 0.0014 | 1.60E-10 | 0.0088 | 40.88 |
| rs11023819 | A | G | 0.577 | 0.008 | 0.0014 | 1.50E-09 | 0.0079 | 36.50 |
| rs11023894 | A | T | 0.510 | 0.008 | 0.0014 | 4.70E-08 | 0.0064 | 29.83 |
| rs35891966 | G | A | 0.929 | 0.016 | 0.0027 | 6.90E-09 | 0.0073 | 33.56 |
| rs530916 | A | G | 0.440 | -0.008 | 0.0014 | 2.00E-08 | 0.0068 | 31.46 |
| rs9554663 | T | C | 0.744 | -0.009 | 0.0016 | 5.60E-09 | 0.0073 | 33.96 |
| rs77653640 | G | A | 0.806 | -0.010 | 0.0018 | 3.30E-08 | 0.0066 | 30.51 |
| rs12442096 | C | T | 0.615 | 0.008 | 0.0014 | 2.50E-09 | 0.0077 | 35.55 |
| rs62012628 | C | T | 0.713 | 0.009 | 0.0015 | 5.20E-09 | 0.0074 | 34.10 |
| rs28635466 | G | A | 0.696 | 0.008 | 0.0015 | 2.00E-08 | 0.0068 | 31.47 |
| rs9889262 | T | A | 0.638 | -0.008 | 0.0014 | 4.00E-08 | 0.0065 | 30.17 |
| rs17733784 | T | C | 0.624 | 0.008 | 0.0014 | 4.60E-09 | 0.0074 | 34.36 |
| rs6050215 | C | T | 0.031 | -0.026 | 0.0040 | 5.70E-11 | 0.0093 | 42.93 |
| rs348829 | G | A | 0.342 | -0.008 | 0.0015 | 1.10E-08 | 0.0071 | 32.70 |
| rs909334 | C | A | 0.752 | 0.010 | 0.0016 | 1.80E-09 | 0.0078 | 36.19 |
| rs5996139 | A | G | 0.363 | 0.008 | 0.0015 | 4.40E-08 | 0.0065 | 29.98 |

**Supplementary table 4.** SNPs associated with alcohol drinking at the genome-wide level of significance (*P* < 5 x 10^-8^) and clumped for independence at kb = 10,000 and *r^2^* = 0.01

| **SNP** | **EA** | **OA** | **EAF** | **BETA** | **SE** | ***P*-value** | **R^2^ %** | **F** |
| --- | --- | --- | --- | --- | --- | --- | --- | --- |
| rs705687 | G | A | 0.785 | -0.011 | 0.002 | 8.20E-10 | 0.0032 | 30.25 |
| rs58107686 | A | C | 0.328 | -0.01 | 0.002 | 7.80E-10 | 0.0027 | 25.00 |
| rs12088813 | C | A | 0.267 | -0.009 | 0.002 | 1.60E-08 | 0.0022 | 20.25 |
| rs5024204 | T | A | 0.278 | 0.01 | 0.002 | 2.60E-09 | 0.0027 | 25.00 |
| rs10753661 | A | G | 0.684 | -0.009 | 0.002 | 3.80E-08 | 0.0022 | 20.25 |
| rs28680958 | A | G | 0.217 | -0.011 | 0.002 | 5.10E-10 | 0.0032 | 30.25 |
| rs823114 | A | G | 0.553 | 0.009 | 0.001 | 2.30E-09 | 0.0086 | 81.00 |
| rs77165542 | T | C | 0.0349 | -0.026 | 0.004 | 5.60E-11 | 0.0045 | 42.25 |
| rs1260326 | C | T | 0.601 | 0.021 | 0.001 | 8.10E-45 | 0.0468 | 441.00 |
| rs13383034 | T | C | 0.329 | 0.015 | 0.002 | 6.30E-22 | 0.0060 | 56.25 |
| rs13032049 | G | A | 0.283 | 0.01 | 0.002 | 3.00E-10 | 0.0027 | 25.00 |
| rs828867 | A | G | 0.545 | 0.009 | 0.001 | 2.20E-09 | 0.0086 | 81.00 |
| rs11692435 | A | G | 0.0852 | 0.017 | 0.003 | 2.50E-11 | 0.0034 | 32.11 |
| rs13024996 | A | C | 0.364 | -0.011 | 0.002 | 5.70E-13 | 0.0032 | 30.25 |
| rs72859280 | T | G | 0.0362 | 0.023 | 0.004 | 4.40E-09 | 0.0035 | 33.06 |
| rs56337305 | C | T | 0.383 | -0.01 | 0.001 | 1.60E-10 | 0.0106 | 100.00 |
| rs13094887 | T | A | 0.301 | -0.01 | 0.002 | 8.60E-11 | 0.0027 | 25.00 |
| rs62250685 | G | A | 0.614 | -0.014 | 0.002 | 1.10E-21 | 0.0052 | 49.00 |
| rs13066454 | T | C | 0.398 | -0.009 | 0.001 | 4.10E-09 | 0.0086 | 81.00 |
| rs9838144 | C | G | 0.209 | -0.01 | 0.002 | 2.70E-08 | 0.0027 | 25.00 |
| rs2011092 | C | T | 0.33864 | -0.009 | 0.002 | 7.40E-09 | 0.0022 | 20.25 |
| rs6787172 | G | T | 0.554 | -0.008 | 0.001 | 4.30E-08 | 0.0068 | 64.00 |
| rs3748034 | T | G | 0.143 | -0.012 | 0.002 | 1.70E-08 | 0.0038 | 36.00 |
| rs11940694 | G | A | 0.597 | 0.026 | 0.001 | 3.00E-68 | 0.0718 | 676.00 |
| rs4501255 | G | C | 0.235 | 0.011 | 0.002 | 4.80E-10 | 0.0032 | 30.25 |
| rs1229984 | C | T | 0.963 | 0.151 | 0.004 | 1.00E-200 | 0.1512 | 1425.06 |
| rs36052336 | G | A | 0.0615 | -0.018 | 0.003 | 1.20E-09 | 0.0038 | 36.00 |
| rs2165670 | A | G | 0.10634 | 0.023 | 0.002 | 1.70E-22 | 0.0140 | 132.25 |
| rs79139602 | T | A | 0.02106 | 0.06 | 0.005 | 1.80E-32 | 0.0153 | 144.00 |
| rs4699791 | A | G | 0.09573 | 0.019 | 0.002 | 6.60E-14 | 0.0096 | 90.25 |
| rs13107325 | T | C | 0.0722 | -0.028 | 0.003 | 1.50E-22 | 0.0093 | 87.11 |
| rs4690727 | G | C | 0.718 | 0.011 | 0.002 | 2.40E-11 | 0.0032 | 30.25 |
| rs12651313 | G | C | 0.443 | -0.009 | 0.001 | 3.80E-09 | 0.0086 | 81.00 |
| rs4916723 | C | A | 0.416 | -0.01 | 0.001 | 1.70E-11 | 0.0106 | 100.00 |
| rs12655091 | A | G | 0.53 | -0.008 | 0.001 | 1.30E-08 | 0.0068 | 64.00 |
| rs55872084 | T | G | 0.235 | 0.01 | 0.002 | 6.30E-09 | 0.0027 | 25.00 |
| rs6460047 | C | T | 0.208 | 0.012 | 0.002 | 9.70E-11 | 0.0038 | 36.00 |
| rs10236149 | G | A | 0.123 | -0.013 | 0.002 | 1.20E-09 | 0.0045 | 42.25 |
| rs35034355 | A | G | 0.521 | -0.008 | 0.001 | 2.90E-08 | 0.0068 | 64.00 |
| rs6951574 | C | T | 0.458 | 0.013 | 0.001 | 1.60E-19 | 0.0180 | 169.00 |
| rs13250583 | T | C | 0.213 | -0.01 | 0.002 | 4.70E-08 | 0.0027 | 25.00 |
| rs1217091 | C | T | 0.812 | 0.012 | 0.002 | 7.10E-11 | 0.0038 | 36.00 |
| rs28601761 | G | C | 0.42 | 0.009 | 0.001 | 7.20E-10 | 0.0086 | 81.00 |
| rs55932213 | G | A | 0.73639 | 0.009 | 0.002 | 9.60E-09 | 0.0022 | 20.25 |
| rs10978550 | C | T | 0.206 | -0.012 | 0.002 | 7.20E-11 | 0.0038 | 36.00 |
| rs7074871 | A | G | 0.255 | -0.009 | 0.002 | 1.90E-08 | 0.0022 | 20.25 |
| rs17665139 | T | C | 0.149 | -0.012 | 0.002 | 1.60E-08 | 0.0038 | 36.00 |
| rs7950166 | T | C | 0.637 | -0.01 | 0.002 | 9.90E-11 | 0.0027 | 25.00 |
| rs11030084 | T | C | 0.184 | -0.011 | 0.002 | 1.70E-08 | 0.0032 | 30.25 |
| rs56030824 | A | G | 0.322 | -0.012 | 0.002 | 1.20E-13 | 0.0038 | 36.00 |
| rs10750025 | T | C | 0.686 | 0.01 | 0.002 | 4.90E-11 | 0.0027 | 25.00 |
| rs1713676 | G | A | 0.52245 | -0.008 | 0.001 | 4.30E-08 | 0.0068 | 64.00 |
| rs4938230 | A | C | 0.842 | 0.013 | 0.002 | 1.50E-10 | 0.0045 | 42.25 |
| rs682011 | C | T | 0.559 | 0.008 | 0.001 | 2.20E-08 | 0.0068 | 64.00 |
| rs12795042 | C | A | 0.623 | -0.008 | 0.002 | 3.30E-08 | 0.0017 | 16.00 |
| rs10876188 | T | C | 0.457 | -0.008 | 0.001 | 4.80E-08 | 0.0068 | 64.00 |
| rs3809162 | G | A | 0.397 | 0.009 | 0.001 | 1.20E-09 | 0.0086 | 81.00 |
| rs10506274 | T | G | 0.484 | -0.009 | 0.001 | 5.80E-10 | 0.0086 | 81.00 |
| rs4842786 | A | G | 0.584 | -0.009 | 0.001 | 2.70E-09 | 0.0086 | 81.00 |
| rs500321 | T | A | 0.736 | -0.01 | 0.002 | 4.90E-09 | 0.0027 | 25.00 |
| rs1123285 | G | C | 0.335 | -0.009 | 0.002 | 8.10E-09 | 0.0022 | 20.25 |
| rs2180870 | C | T | 0.135 | -0.012 | 0.002 | 1.10E-08 | 0.0038 | 36.00 |
| rs28929474 | T | C | 0.0183 | -0.037 | 0.005 | 1.30E-11 | 0.0058 | 54.76 |
| rs11625650 | A | G | 0.233 | -0.01 | 0.002 | 2.90E-08 | 0.0027 | 25.00 |
| rs2472297 | T | C | 0.249 | 0.011 | 0.002 | 3.10E-10 | 0.0032 | 30.25 |
| rs12907323 | G | A | 0.411 | 0.008 | 0.001 | 9.90E-09 | 0.0068 | 64.00 |
| rs2764771 | A | G | 0.307 | 0.01 | 0.002 | 4.00E-10 | 0.0027 | 25.00 |
| rs17177078 | T | C | 0.0626 | -0.022 | 0.003 | 1.30E-13 | 0.0057 | 53.78 |
| rs378421 | A | G | 0.404 | -0.011 | 0.001 | 4.80E-14 | 0.0129 | 121.00 |
| rs113443718 | A | G | 0.305 | -0.01 | 0.002 | 1.20E-10 | 0.0027 | 25.00 |
| rs62044525 | G | C | 0.184 | -0.012 | 0.002 | 1.00E-10 | 0.0038 | 36.00 |
| rs7185555 | C | G | 0.153 | -0.011 | 0.002 | 4.20E-08 | 0.0032 | 30.25 |
| rs79616692 | C | G | 0.108 | 0.016 | 0.002 | 4.10E-12 | 0.0068 | 64.00 |
| rs1104608 | C | G | 0.425 | -0.011 | 0.001 | 1.10E-13 | 0.0129 | 121.00 |
| rs4548913 | A | G | 0.632 | -0.008 | 0.002 | 3.10E-08 | 0.0017 | 16.00 |
| rs3803800 | G | A | 0.786 | 0.011 | 0.002 | 1.50E-10 | 0.0032 | 30.25 |
| rs2854334 | G | A | 0.615 | 0.009 | 0.001 | 7.50E-10 | 0.0086 | 81.00 |
| rs2532276 | A | C | 0.215 | -0.022 | 0.003 | 1.60E-17 | 0.0057 | 53.78 |
| rs10438820 | T | C | 0.702 | 0.009 | 0.002 | 1.80E-08 | 0.0022 | 20.25 |
| rs9950000 | T | C | 0.395 | -0.009 | 0.001 | 9.40E-10 | 0.0086 | 81.00 |
| rs4092465 | G | A | 0.635 | -0.008 | 0.002 | 4.40E-08 | 0.0017 | 16.00 |
| rs281379 | A | G | 0.508 | 0.014 | 0.001 | 4.90E-21 | 0.0208 | 196.00 |
| rs4815364 | A | G | 0.616 | 0.009 | 0.001 | 1.00E-08 | 0.0086 | 81.00 |
| rs9607814 | A | C | 0.2 | -0.01 | 0.002 | 4.30E-08 | 0.0027 | 25.00 |

**Supplementary table 5.** SNPs associated with sleep duration at the genome-wide level of significance (*P* < 5 x 10^-8^) and clumped for independence at kb = 10,000 and *r^2^* = 0.01

| **SNP** | **EA** | **OA** | **EAF** | **BETA** | **SE** | ***P*-value** | **R^2^ %** | **F** |
| --- | --- | --- | --- | --- | --- | --- | --- | --- |
| rs4642942 | C | G | 0.415 | 0.01 | 0.0025 | 3.00E-08 | 0.0042 | 16.10 |
| rs915416 | C | G | 0.291 | 0.02 | 0.0027 | 8.00E-12 | 0.0143 | 55.11 |
| rs540431 | G | A | 0.450 | -0.01 | 0.0025 | 5.00E-09 | 0.0043 | 16.46 |
| rs1392817 | A | G | 0.477 | 0.01 | 0.0025 | 2.00E-08 | 0.0042 | 16.09 |
| rs79512144 | A | G | 0.065 | -0.03 | 0.0050 | 4.00E-08 | 0.0095 | 36.41 |
| rs11682175 | C | T | 0.473 | 0.02 | 0.0025 | 1.00E-13 | 0.0172 | 66.26 |
| rs116219610 | T | C | 0.193 | 0.02 | 0.0031 | 5.00E-14 | 0.0108 | 41.36 |
| rs62158206 | C | T | 0.219 | 0.04 | 0.0030 | 3.00E-43 | 0.0472 | 181.63 |
| rs35662245 | A | T | 0.339 | 0.02 | 0.0026 | 6.00E-09 | 0.0156 | 60.00 |
| rs11883686 | T | A | 0.332 | -0.01 | 0.0026 | 1.00E-08 | 0.0038 | 14.78 |
| rs12463754 | G | C | 0.271 | 0.02 | 0.0028 | 4.00E-10 | 0.0136 | 52.09 |
| rs112230981 | G | A | 0.049 | -0.03 | 0.0057 | 4.90E-08 | 0.0072 | 27.72 |
| rs4688116 | G | T | 0.403 | -0.01 | 0.0025 | 4.00E-08 | 0.0041 | 15.90 |
| rs16834426 | A | G | 0.269 | 0.02 | 0.0028 | 2.00E-08 | 0.0136 | 52.40 |
| rs9843801 | C | T | 0.341 | 0.02 | 0.0026 | 6.00E-10 | 0.0156 | 59.91 |
| rs2192528 | A | G | 0.477 | 0.02 | 0.0025 | 6.00E-10 | 0.0173 | 66.53 |
| rs7686205 | G | A | 0.379 | 0.01 | 0.0025 | 1.00E-08 | 0.0041 | 15.76 |
| rs41501452 | G | A | 0.316 | -0.02 | 0.0026 | 4.00E-09 | 0.0150 | 57.70 |
| rs17285646 | A | T | 0.476 | 0.01 | 0.0025 | 2.00E-08 | 0.0043 | 16.63 |
| rs13109404 | G | T | 0.070 | -0.03 | 0.0048 | 1.00E-11 | 0.0102 | 39.14 |
| rs365663 | G | A | 0.455 | -0.02 | 0.0025 | 1.00E-11 | 0.0172 | 65.99 |
| rs6889592 | A | G | 0.332 | 0.02 | 0.0026 | 1.00E-11 | 0.0154 | 59.35 |
| rs113322698 | A | G | 0.342 | 0.01 | 0.0026 | 2.00E-08 | 0.0039 | 15.09 |
| rs34388845 | G | A | 0.214 | -0.02 | 0.0030 | 5.00E-10 | 0.0117 | 44.95 |
| rs9451146 | T | C | 0.228 | -0.02 | 0.0029 | 9.00E-09 | 0.0122 | 47.07 |
| rs9362971 | C | T | 0.346 | -0.01 | 0.0026 | 1.00E-08 | 0.0039 | 15.18 |
| rs3823624 | C | T | 0.181 | 0.02 | 0.0032 | 1.00E-09 | 0.0102 | 39.36 |
| rs6979198 | G | T | 0.211 | 0.02 | 0.0030 | 1.00E-08 | 0.0116 | 44.44 |
| rs1668331 | T | G | 0.374 | -0.02 | 0.0025 | 5.00E-10 | 0.0161 | 62.00 |
| rs12336359 | C | G | 0.404 | 0.01 | 0.0025 | 9.00E-09 | 0.0041 | 15.71 |
| rs10973207 | T | G | 0.155 | 0.02 | 0.0034 | 2.00E-09 | 0.0091 | 34.79 |
| rs144625846 | G | A | 0.135 | -0.02 | 0.0036 | 1.00E-08 | 0.0081 | 31.30 |
| rs7915425 | T | C | 0.176 | 0.02 | 0.0032 | 3.00E-10 | 0.0100 | 38.55 |
| rs7115856 | C | A | 0.461 | 0.01 | 0.0025 | 1.00E-09 | 0.0043 | 16.63 |
| rs12791153 | T | A | 0.077 | 0.03 | 0.0046 | 3.00E-08 | 0.0110 | 42.28 |
| rs1553132 | G | A | 0.259 | 0.02 | 0.0028 | 7.00E-09 | 0.0134 | 51.46 |
| rs1263056 | G | A | 0.482 | -0.01 | 0.0025 | 4.00E-08 | 0.0043 | 16.38 |
| rs4767550 | G | A | 0.413 | 0.01 | 0.0025 | 3.00E-09 | 0.0041 | 15.91 |
| rs11621908 | T | C | 0.080 | -0.03 | 0.0045 | 5.00E-09 | 0.0113 | 43.51 |
| rs13329140 | A | G | 0.236 | -0.02 | 0.0029 | 8.00E-11 | 0.0125 | 47.96 |
| rs77684884 | G | A | 0.188 | 0.02 | 0.0032 | 6.00E-10 | 0.0102 | 39.14 |
| rs28651105 | G | A | 0.217 | 0.02 | 0.0030 | 4.90E-08 | 0.0118 | 45.38 |
| rs8047587 | T | G | 0.440 | -0.02 | 0.0025 | 1.00E-12 | 0.0171 | 65.67 |
| rs11076146 | G | T | 0.440 | 0.01 | 0.0025 | 3.00E-09 | 0.0043 | 16.51 |
| rs11654671 | T | A | 0.235 | 0.02 | 0.0029 | 4.00E-08 | 0.0121 | 46.66 |
| rs11650677 | A | G | 0.339 | 0.01 | 0.0026 | 9.00E-09 | 0.0039 | 15.02 |
| rs12607679 | C | T | 0.260 | -0.02 | 0.0028 | 1.00E-10 | 0.0131 | 50.26 |

**Supplementary table 6.** SNPs associated with insomnia at the genome-wide level of significance (*P* < 5 x 10^-8^) and clumped for independence at kb = 10,000 and *r^2^* = 0.01

| **SNP** | **EA** | **OA** | **EAF** | **BETA** | **SE** | ***P*-value** | **R^2^ %** | **F** |
| --- | --- | --- | --- | --- | --- | --- | --- | --- |
| rs2089358 | C | T | 0.3 | 0.041 | 0.007 | 2.75E-10 | 0.0026 | 34.31 |
| rs11588755 | G | A | 0.48 | 0.035 | 0.006 | 5.14E-09 | 0.0026 | 34.03 |
| rs1937447 | G | C | 0.24 | 0.039 | 0.007 | 2.08E-08 | 0.0023 | 31.04 |
| rs1620977 | A | G | 0.27 | 0.052 | 0.007 | 2.27E-14 | 0.0041 | 55.18 |
| rs699844 | A | G | 0.92 | 0.06 | 0.011 | 4.11E-08 | 0.0022 | 29.75 |
| rs12030482 | A | T | 0.22 | 0.041 | 0.007 | 8.16E-09 | 0.0026 | 34.31 |
| rs6702604 | G | A | 0.42 | 0.037 | 0.006 | 1.30E-09 | 0.0029 | 38.03 |
| rs1289939 | C | T | 0.77 | 0.041 | 0.007 | 6.00E-09 | 0.0026 | 34.31 |
| rs5877 | T | C | 0.67 | 0.036 | 0.006 | 1.23E-08 | 0.0027 | 36.00 |
| rs11803128 | G | A | 0.35 | 0.041 | 0.006 | 6.85E-11 | 0.0035 | 46.69 |
| rs10800992 | T | C | 0.44 | 0.042 | 0.006 | 3.84E-12 | 0.0037 | 49.00 |
| rs623025 | C | T | 0.74 | 0.038 | 0.007 | 3.16E-08 | 0.0022 | 29.47 |
| rs11119409 | C | T | 0.41 | 0.035 | 0.006 | 1.19E-08 | 0.0026 | 34.03 |
| rs823247 | C | T | 0.52 | 0.037 | 0.006 | 5.25E-10 | 0.0029 | 38.03 |
| rs6734957 | G | T | 0.76 | 0.042 | 0.007 | 1.82E-09 | 0.0027 | 36.00 |
| rs56097173 | T | C | 0.68 | 0.04 | 0.006 | 2.69E-10 | 0.0033 | 44.44 |
| rs13010288 | G | T | 0.87 | 0.06 | 0.009 | 9.26E-12 | 0.0033 | 44.44 |
| rs1861412 | A | G | 0.43 | 0.038 | 0.006 | 1.67E-10 | 0.0030 | 40.11 |
| rs6545798 | T | A | 0.59 | 0.041 | 0.006 | 1.19E-11 | 0.0035 | 46.69 |
| rs1519102 | G | C | 0.31 | 0.037 | 0.006 | 1.90E-08 | 0.0029 | 38.03 |
| rs113851554 | T | G | 0.05 | 0.206 | 0.014 | 1.56E-51 | 0.0163 | 216.51 |
| rs75452188 | A | G | 0.88 | 0.052 | 0.009 | 1.58E-08 | 0.0025 | 33.38 |
| rs12991815 | C | G | 0.42 | 0.04 | 0.006 | 3.02E-11 | 0.0033 | 44.44 |
| rs11679943 | A | G | 0.35 | 0.037 | 0.006 | 3.16E-09 | 0.0029 | 38.03 |
| rs72820274 | A | G | 0.42 | 0.034 | 0.006 | 1.28E-08 | 0.0024 | 32.11 |
| rs62158170 | A | G | 0.79 | 0.066 | 0.007 | 1.20E-19 | 0.0067 | 88.90 |
| rs10928256 | T | C | 0.42 | 0.034 | 0.006 | 1.61E-08 | 0.0024 | 32.11 |
| rs6756610 | C | G | 0.63 | 0.037 | 0.006 | 1.14E-09 | 0.0029 | 38.03 |
| rs116466468 | T | C | 0.76 | 0.044 | 0.007 | 2.11E-10 | 0.0030 | 39.51 |
| rs4664299 | C | T | 0.77 | 0.041 | 0.007 | 4.95E-09 | 0.0026 | 34.31 |
| rs7571486 | G | A | 0.75 | 0.039 | 0.007 | 1.40E-08 | 0.0023 | 31.04 |
| rs55772859 | A | C | 0.31 | 0.042 | 0.006 | 4.82E-11 | 0.0037 | 49.00 |
| rs62213452 | T | G | 0.28 | 0.037 | 0.007 | 2.39E-08 | 0.0021 | 27.94 |
| rs34967082 | A | G | 0.41 | 0.035 | 0.006 | 4.34E-09 | 0.0026 | 34.03 |
| rs1530938 | A | G | 0.44 | 0.036 | 0.006 | 8.82E-10 | 0.0027 | 36.00 |
| rs7599697 | C | T | 0.64 | 0.037 | 0.006 | 5.00E-09 | 0.0029 | 38.03 |
| rs6808140 | T | C | 0.51 | 0.039 | 0.006 | 5.35E-11 | 0.0032 | 42.25 |
| rs7615602 | G | C | 0.73 | 0.04 | 0.007 | 2.59E-09 | 0.0025 | 32.65 |
| rs4858708 | T | A | 0.47 | 0.034 | 0.006 | 1.23E-08 | 0.0024 | 32.11 |
| rs35110063 | A | G | 0.43 | 0.039 | 0.006 | 8.82E-11 | 0.0032 | 42.25 |
| rs7625896 | A | G | 0.65 | 0.036 | 0.006 | 5.28E-09 | 0.0027 | 36.00 |
| rs10865954 | T | C | 0.33 | 0.042 | 0.006 | 1.92E-11 | 0.0037 | 49.00 |
| rs3774751 | G | T | 0.54 | 0.041 | 0.006 | 7.32E-12 | 0.0035 | 46.69 |
| rs1567084 | A | G | 0.5 | 0.033 | 0.006 | 2.14E-08 | 0.0023 | 30.25 |
| rs17025198 | A | G | 0.2 | 0.041 | 0.007 | 2.19E-08 | 0.0026 | 34.31 |
| rs1580173 | A | G | 0.56 | 0.033 | 0.006 | 2.28E-08 | 0.0023 | 30.25 |
| rs62264767 | A | C | 0.85 | 0.065 | 0.008 | 1.63E-14 | 0.0050 | 66.02 |
| rs492858 | C | T | 0.92 | 0.066 | 0.011 | 3.46E-09 | 0.0027 | 36.00 |
| rs2364921 | C | T | 0.53 | 0.034 | 0.006 | 2.13E-08 | 0.0024 | 32.11 |
| rs694786 | C | T | 0.54 | 0.044 | 0.006 | 1.97E-13 | 0.0040 | 53.78 |
| rs4260410 | T | C | 0.33 | 0.034 | 0.006 | 4.87E-08 | 0.0024 | 32.11 |
| rs2216427 | C | G | 0.65 | 0.035 | 0.006 | 1.60E-08 | 0.0026 | 34.03 |
| rs62301574 | G | C | 0.2 | 0.042 | 0.007 | 1.37E-08 | 0.0027 | 36.00 |
| rs16990210 | C | T | 0.15 | 0.046 | 0.008 | 1.97E-08 | 0.0025 | 33.06 |
| rs17005118 | A | G | 0.26 | 0.042 | 0.007 | 6.13E-10 | 0.0027 | 36.00 |
| rs72657797 | C | T | 0.82 | 0.056 | 0.008 | 1.52E-12 | 0.0037 | 49.00 |
| rs13135092 | G | A | 0.08 | 0.089 | 0.011 | 2.53E-16 | 0.0049 | 65.46 |
| rs4699157 | C | T | 0.04 | 0.081 | 0.015 | 3.98E-08 | 0.0022 | 29.16 |
| rs11722569 | T | C | 0.66 | 0.034 | 0.006 | 2.91E-08 | 0.0024 | 32.11 |
| rs13138995 | A | G | 0.39 | 0.034 | 0.006 | 1.97E-08 | 0.0024 | 32.11 |
| rs17223714 | A | G | 0.79 | 0.046 | 0.007 | 2.44E-10 | 0.0032 | 43.18 |
| rs12520974 | C | T | 0.52 | 0.036 | 0.006 | 1.69E-09 | 0.0027 | 36.00 |
| rs701394 | G | A | 0.36 | 0.036 | 0.006 | 6.83E-09 | 0.0027 | 36.00 |
| rs16903122 | T | C | 0.25 | 0.055 | 0.007 | 9.04E-16 | 0.0046 | 61.73 |
| rs35539975 | A | G | 0.78 | 0.042 | 0.007 | 4.49E-09 | 0.0027 | 36.00 |
| rs17083297 | C | A | 0.82 | 0.044 | 0.008 | 1.60E-08 | 0.0023 | 30.25 |
| rs12187443 | T | C | 0.67 | 0.04 | 0.006 | 1.64E-10 | 0.0033 | 44.44 |
| rs2431108 | C | T | 0.33 | 0.053 | 0.006 | 7.83E-17 | 0.0059 | 78.03 |
| rs152555 | G | A | 0.15 | 0.052 | 0.008 | 4.83E-10 | 0.0032 | 42.25 |
| rs17367725 | C | T | 0.65 | 0.036 | 0.006 | 9.29E-09 | 0.0027 | 36.00 |
| rs8180457 | C | T | 0.84 | 0.056 | 0.008 | 1.12E-11 | 0.0037 | 49.00 |
| rs55972276 | A | C | 0.14 | 0.073 | 0.009 | 4.19E-17 | 0.0049 | 65.79 |
| rs6888135 | A | C | 0.5 | 0.038 | 0.006 | 1.21E-10 | 0.0030 | 40.11 |
| rs4502882 | C | T | 0.34 | 0.039 | 0.006 | 7.96E-10 | 0.0032 | 42.25 |
| rs62383308 | G | A | 0.92 | 0.06 | 0.011 | 3.98E-08 | 0.0022 | 29.75 |
| rs6601080 | A | G | 0.68 | 0.035 | 0.006 | 2.21E-08 | 0.0026 | 34.03 |
| rs11756035 | C | G | 0.13 | 0.051 | 0.009 | 1.29E-08 | 0.0024 | 32.11 |
| rs138678612 | G | A | 0.02 | 0.117 | 0.02 | 1.41E-08 | 0.0026 | 34.22 |
| rs3131638 | G | A | 0.77 | 0.044 | 0.007 | 7.88E-10 | 0.0030 | 39.51 |
| rs10947428 | C | T | 0.21 | 0.068 | 0.007 | 9.06E-21 | 0.0071 | 94.37 |
| rs6457796 | C | T | 0.27 | 0.039 | 0.007 | 1.12E-08 | 0.0023 | 31.04 |
| rs10947690 | G | A | 0.26 | 0.047 | 0.007 | 4.04E-12 | 0.0034 | 45.08 |
| rs9394502 | C | T | 0.67 | 0.054 | 0.006 | 7.76E-18 | 0.0061 | 81.00 |
| rs10947987 | C | T | 0.56 | 0.033 | 0.006 | 4.08E-08 | 0.0023 | 30.25 |
| rs10944696 | G | A | 0.7 | 0.038 | 0.007 | 7.99E-09 | 0.0022 | 29.47 |
| rs2388840 | G | A | 0.42 | 0.037 | 0.006 | 1.37E-09 | 0.0029 | 38.03 |
| rs9373590 | A | T | 0.51 | 0.04 | 0.006 | 2.18E-11 | 0.0033 | 44.44 |
| rs314281 | C | T | 0.55 | 0.043 | 0.006 | 6.03E-13 | 0.0039 | 51.36 |
| rs728017 | G | A | 0.61 | 0.035 | 0.006 | 9.51E-09 | 0.0026 | 34.03 |
| rs62429521 | A | C | 0.15 | 0.051 | 0.008 | 1.78E-09 | 0.0031 | 40.64 |
| rs1147852 | A | G | 0.31 | 0.039 | 0.006 | 9.94E-10 | 0.0032 | 42.25 |
| rs4709655 | C | T | 0.88 | 0.054 | 0.009 | 3.09E-09 | 0.0027 | 36.00 |
| rs117152417 | G | A | 0.99 | 0.147 | 0.026 | 2.82E-08 | 0.0024 | 31.97 |
| rs6978112 | T | C | 0.41 | 0.034 | 0.006 | 2.11E-08 | 0.0024 | 32.11 |
| rs940780 | T | C | 0.36 | 0.038 | 0.006 | 8.50E-10 | 0.0030 | 40.11 |
| rs190073 | G | A | 0.59 | 0.034 | 0.006 | 2.86E-08 | 0.0024 | 32.11 |
| rs2030672 | C | G | 0.56 | 0.034 | 0.006 | 1.10E-08 | 0.0024 | 32.11 |
| rs521484 | G | A | 0.23 | 0.04 | 0.007 | 1.53E-08 | 0.0025 | 32.65 |
| rs6465151 | T | C | 0.11 | 0.056 | 0.009 | 1.90E-09 | 0.0029 | 38.72 |
| rs75932578 | C | T | 0.78 | 0.04 | 0.007 | 4.15E-08 | 0.0025 | 32.65 |
| rs670501 | T | C | 0.21 | 0.053 | 0.007 | 7.40E-13 | 0.0043 | 57.33 |
| rs8180817 | G | C | 0.57 | 0.049 | 0.006 | 1.83E-16 | 0.0050 | 66.69 |
| rs12666306 | A | G | 0.5 | 0.042 | 0.006 | 2.24E-12 | 0.0037 | 49.00 |
| rs17520265 | G | A | 0.97 | 0.091 | 0.016 | 2.87E-08 | 0.0024 | 32.35 |
| rs6967168 | G | T | 0.25 | 0.044 | 0.007 | 1.39E-10 | 0.0030 | 39.51 |
| rs2598293 | T | C | 0.48 | 0.035 | 0.006 | 2.48E-09 | 0.0026 | 34.03 |
| rs1731951 | T | A | 0.56 | 0.035 | 0.006 | 1.36E-08 | 0.0026 | 34.03 |
| rs28611339 | T | G | 0.13 | 0.058 | 0.009 | 8.46E-11 | 0.0031 | 41.53 |
| rs874168 | T | C | 0.53 | 0.034 | 0.006 | 7.95E-09 | 0.0024 | 32.11 |
| rs871994 | A | C | 0.44 | 0.035 | 0.006 | 5.50E-09 | 0.0026 | 34.03 |
| rs671985 | G | A | 0.55 | 0.038 | 0.006 | 2.79E-10 | 0.0030 | 40.11 |
| rs4588900 | A | G | 0.52 | 0.033 | 0.006 | 1.57E-08 | 0.0023 | 30.25 |
| rs17643634 | C | T | 0.84 | 0.06 | 0.008 | 1.34E-13 | 0.0042 | 56.25 |
| rs28552587 | A | G | 0.56 | 0.033 | 0.006 | 3.30E-08 | 0.0023 | 30.25 |
| rs10955647 | T | G | 0.53 | 0.033 | 0.006 | 1.84E-08 | 0.0023 | 30.25 |
| rs2737240 | A | G | 0.71 | 0.036 | 0.007 | 3.37E-08 | 0.0020 | 26.45 |
| rs10758593 | G | A | 0.6 | 0.036 | 0.006 | 4.90E-09 | 0.0027 | 36.00 |
| rs118166957 | T | C | 0.16 | 0.068 | 0.008 | 1.95E-16 | 0.0054 | 72.25 |
| rs10756571 | T | C | 0.69 | 0.036 | 0.006 | 1.80E-08 | 0.0027 | 36.00 |
| rs4090240 | C | T | 0.72 | 0.039 | 0.007 | 8.46E-09 | 0.0023 | 31.04 |
| rs7044885 | G | C | 0.56 | 0.041 | 0.006 | 5.67E-12 | 0.0035 | 46.69 |
| rs10761240 | G | A | 0.6 | 0.043 | 0.006 | 2.12E-12 | 0.0039 | 51.36 |
| rs1927902 | T | C | 0.25 | 0.053 | 0.007 | 1.15E-14 | 0.0043 | 57.33 |
| rs2792990 | C | G | 0.86 | 0.054 | 0.008 | 1.15E-10 | 0.0034 | 45.56 |
| rs6597649 | T | C | 0.4 | 0.033 | 0.006 | 3.05E-08 | 0.0023 | 30.25 |
| rs7040224 | A | G | 0.32 | 0.037 | 0.006 | 4.24E-09 | 0.0029 | 38.03 |
| rs72773790 | T | C | 0.67 | 0.037 | 0.006 | 3.71E-09 | 0.0029 | 38.03 |
| rs77641763 | T | C | 0.12 | 0.071 | 0.009 | 6.53E-15 | 0.0047 | 62.23 |
| rs12251016 | T | A | 0.34 | 0.039 | 0.006 | 3.89E-10 | 0.0032 | 42.25 |
| rs10825503 | T | G | 0.49 | 0.033 | 0.006 | 1.43E-08 | 0.0023 | 30.25 |
| rs224029 | C | T | 0.6 | 0.039 | 0.006 | 2.51E-10 | 0.0032 | 42.25 |
| rs11001276 | T | A | 0.26 | 0.038 | 0.007 | 2.52E-08 | 0.0022 | 29.47 |
| rs7475916 | G | C | 0.65 | 0.037 | 0.006 | 6.70E-09 | 0.0029 | 38.03 |
| rs214934 | T | A | 0.69 | 0.038 | 0.006 | 3.16E-09 | 0.0030 | 40.11 |
| rs72899452 | T | C | 0.06 | 0.074 | 0.012 | 1.00E-09 | 0.0029 | 38.03 |
| rs11605348 | G | A | 0.65 | 0.045 | 0.006 | 7.01E-13 | 0.0042 | 56.25 |
| rs12790660 | C | T | 0.32 | 0.04 | 0.006 | 4.49E-10 | 0.0033 | 44.44 |
| rs4592425 | T | G | 0.7 | 0.04 | 0.006 | 4.31E-10 | 0.0033 | 44.44 |
| rs524859 | G | A | 0.64 | 0.044 | 0.006 | 1.48E-12 | 0.0040 | 53.78 |
| rs566673 | G | T | 0.46 | 0.039 | 0.006 | 1.18E-10 | 0.0032 | 42.25 |
| rs56133505 | A | G | 0.54 | 0.041 | 0.006 | 5.59E-12 | 0.0035 | 46.69 |
| rs10898940 | A | C | 0.52 | 0.034 | 0.006 | 8.09E-09 | 0.0024 | 32.11 |
| rs667730 | T | C | 0.58 | 0.033 | 0.006 | 2.26E-08 | 0.0023 | 30.25 |
| rs2221119 | C | G | 0.44 | 0.036 | 0.006 | 2.00E-09 | 0.0027 | 36.00 |
| rs6589988 | G | A | 0.32 | 0.038 | 0.006 | 4.70E-09 | 0.0030 | 40.11 |
| rs1064939 | A | T | 0.98 | 0.13 | 0.02 | 2.16E-10 | 0.0032 | 42.25 |
| rs647905 | T | C | 0.54 | 0.033 | 0.006 | 2.87E-08 | 0.0023 | 30.25 |
| rs2286729 | A | G | 0.09 | 0.07 | 0.011 | 5.37E-11 | 0.0030 | 40.50 |
| rs1167132 | T | C | 0.39 | 0.035 | 0.006 | 8.73E-09 | 0.0026 | 34.03 |
| rs324017 | A | C | 0.29 | 0.039 | 0.007 | 1.61E-09 | 0.0023 | 31.04 |
| rs61921611 | C | T | 0.31 | 0.044 | 0.006 | 7.84E-12 | 0.0040 | 53.78 |
| rs7486418 | T | G | 0.66 | 0.041 | 0.006 | 6.84E-11 | 0.0035 | 46.69 |
| rs6606731 | A | T | 0.19 | 0.043 | 0.008 | 1.51E-08 | 0.0022 | 28.89 |
| rs4767645 | G | T | 0.54 | 0.037 | 0.006 | 6.47E-10 | 0.0029 | 38.03 |
| rs28582096 | G | A | 0.8 | 0.054 | 0.007 | 1.74E-13 | 0.0045 | 59.51 |
| rs9527083 | G | A | 0.33 | 0.076 | 0.006 | 1.61E-32 | 0.0121 | 160.44 |
| rs1031654 | C | A | 0.2 | 0.051 | 0.007 | 3.88E-12 | 0.0040 | 53.08 |
| rs7992992 | A | G | 0.13 | 0.051 | 0.009 | 1.15E-08 | 0.0024 | 32.11 |
| rs6562066 | T | C | 0.37 | 0.039 | 0.006 | 1.38E-10 | 0.0032 | 42.25 |
| rs9563886 | C | T | 0.39 | 0.034 | 0.006 | 3.08E-08 | 0.0024 | 32.11 |
| rs9540729 | A | T | 0.48 | 0.036 | 0.006 | 1.40E-09 | 0.0027 | 36.00 |
| rs11149313 | A | G | 0.73 | 0.04 | 0.007 | 2.38E-09 | 0.0025 | 32.65 |
| rs2389631 | C | A | 0.33 | 0.04 | 0.006 | 2.03E-10 | 0.0033 | 44.44 |
| rs1536053 | C | T | 0.68 | 0.038 | 0.006 | 6.04E-09 | 0.0030 | 40.11 |
| rs4981170 | G | A | 0.81 | 0.054 | 0.008 | 7.33E-13 | 0.0034 | 45.56 |
| rs12912299 | C | T | 0.51 | 0.043 | 0.006 | 4.42E-13 | 0.0039 | 51.36 |
| rs715338 | A | G | 0.58 | 0.041 | 0.006 | 7.85E-12 | 0.0035 | 46.69 |
| rs7168238 | C | G | 0.07 | 0.064 | 0.011 | 1.80E-08 | 0.0025 | 33.85 |
| rs1038093 | T | C | 0.63 | 0.039 | 0.006 | 2.47E-10 | 0.0032 | 42.25 |
| rs12917449 | C | A | 0.19 | 0.042 | 0.008 | 2.97E-08 | 0.0021 | 27.56 |
| rs176644 | T | G | 0.4 | 0.035 | 0.006 | 9.49E-09 | 0.0026 | 34.03 |
| rs4702 | G | A | 0.44 | 0.048 | 0.006 | 6.78E-16 | 0.0048 | 64.00 |
| rs7402939 | C | T | 0.62 | 0.036 | 0.006 | 5.19E-09 | 0.0027 | 36.00 |
| rs3184470 | G | A | 0.65 | 0.038 | 0.006 | 9.73E-10 | 0.0030 | 40.11 |
| rs12924275 | T | C | 0.27 | 0.038 | 0.007 | 1.93E-08 | 0.0022 | 29.47 |
| rs830716 | C | G | 0.71 | 0.045 | 0.007 | 8.68E-12 | 0.0031 | 41.33 |
| rs66674044 | T | A | 0.14 | 0.06 | 0.009 | 2.18E-12 | 0.0033 | 44.44 |
| rs4788203 | G | A | 0.57 | 0.035 | 0.006 | 6.32E-09 | 0.0026 | 34.03 |
| rs1015438 | A | G | 0.19 | 0.058 | 0.008 | 2.51E-14 | 0.0039 | 52.56 |
| rs34214423 | A | C | 0.81 | 0.045 | 0.008 | 3.18E-09 | 0.0024 | 31.64 |
| rs4238755 | C | A | 0.74 | 0.043 | 0.007 | 2.30E-10 | 0.0028 | 37.73 |
| rs9931543 | T | C | 0.74 | 0.048 | 0.007 | 1.11E-12 | 0.0035 | 47.02 |
| rs3902952 | T | C | 0.19 | 0.048 | 0.008 | 2.55E-10 | 0.0027 | 36.00 |
| rs35322724 | A | C | 0.58 | 0.049 | 0.006 | 3.75E-16 | 0.0050 | 66.69 |
| rs62068188 | T | C | 0.83 | 0.049 | 0.008 | 1.18E-09 | 0.0028 | 37.52 |
| rs34490907 | C | G | 0.89 | 0.054 | 0.009 | 1.76E-08 | 0.0027 | 36.00 |
| rs7214267 | G | A | 0.42 | 0.044 | 0.006 | 5.09E-13 | 0.0040 | 53.78 |
| rs11650304 | C | G | 0.93 | 0.067 | 0.012 | 1.23E-08 | 0.0023 | 31.17 |
| rs4643373 | T | C | 0.7 | 0.041 | 0.007 | 1.58E-10 | 0.0026 | 34.31 |
| rs9889282 | C | A | 0.39 | 0.042 | 0.006 | 4.70E-12 | 0.0037 | 49.00 |
| rs8076183 | C | T | 0.55 | 0.038 | 0.006 | 2.75E-10 | 0.0030 | 40.11 |
| rs12454003 | G | C | 0.52 | 0.035 | 0.006 | 4.94E-09 | 0.0026 | 34.03 |
| rs12605642 | T | G | 0.49 | 0.035 | 0.006 | 2.13E-09 | 0.0026 | 34.03 |
| rs10502966 | G | A | 0.42 | 0.039 | 0.006 | 8.54E-11 | 0.0032 | 42.25 |
| rs60565673 | G | T | 0.38 | 0.043 | 0.006 | 1.59E-12 | 0.0039 | 51.36 |
| rs9964420 | A | C | 0.3 | 0.035 | 0.007 | 4.54E-08 | 0.0019 | 25.00 |
| rs12983032 | G | A | 0.66 | 0.043 | 0.006 | 1.07E-11 | 0.0039 | 51.36 |
| rs6510033 | G | A | 0.27 | 0.037 | 0.007 | 4.66E-08 | 0.0021 | 27.94 |
| rs429358 | T | C | 0.85 | 0.046 | 0.008 | 2.13E-08 | 0.0025 | 33.06 |
| rs908668 | T | C | 0.21 | 0.05 | 0.007 | 1.41E-11 | 0.0038 | 51.02 |
| rs6119267 | G | C | 0.31 | 0.06 | 0.006 | 2.32E-20 | 0.0075 | 100.00 |
| rs2867690 | T | C | 0.18 | 0.042 | 0.008 | 3.70E-08 | 0.0021 | 27.56 |
| rs910187 | G | A | 0.63 | 0.035 | 0.006 | 1.63E-08 | 0.0026 | 34.03 |
| rs6019663 | T | C | 0.29 | 0.04 | 0.007 | 6.47E-10 | 0.0025 | 32.65 |
| rs742760 | A | T | 0.82 | 0.043 | 0.008 | 2.48E-08 | 0.0022 | 28.89 |
| rs76145129 | G | T | 0.88 | 0.05 | 0.009 | 2.73E-08 | 0.0023 | 30.86 |
| rs2838787 | G | A | 0.61 | 0.036 | 0.006 | 7.65E-09 | 0.0027 | 36.00 |
| rs11090039 | A | G | 0.29 | 0.039 | 0.007 | 1.82E-09 | 0.0023 | 31.04 |

**Supplementary table 7.** SNPs associated with average accelerometer based physical activity at the genome-wide level of significance (*P* < 5 x 10^-8^) and clumped for independence at kb = 10,000 and *r^2^* = 0.01

| **SNP** | **EA** | **OA** | **EAF** | **BETA** | **SE** | ***P*-value** | **R^2^ %** | **F** |
| --- | --- | --- | --- | --- | --- | --- | --- | --- |
| rs336605 | G | T | 0.276 | 0.222 | 0.041 | 4.50E-08 | 0.0322 | 29.32 |
| rs10067451 | G | A | 0.887 | 0.326 | 0.058 | 2.00E-08 | 0.0347 | 31.59 |
| rs148193266 | A | C | 0.957 | -0.51 | 0.092 | 3.10E-08 | 0.0337 | 30.73 |
| rs79724577 | A | C | 0.818 | -0.276 | 0.047 | 4.60E-09 | 0.0378 | 34.48 |
| rs1518139 | G | T | 0.662 | -0.226 | 0.039 | 4.50E-09 | 0.0369 | 33.58 |

**Supplementary table 8.** SNPs associated with coffee consumption at the genome-wide level of significance (*P* < 5 x 10^-8^) and clumped for independence at kb = 10,000 and *r^2^* = 0.01

| **SNP** | **EA** | **OA** | **EAF** | **BETA** | **SE** | ***P*-value** | **R^2^ %** | **F** |
| --- | --- | --- | --- | --- | --- | --- | --- | --- |
| rs2472297 | T | C | 0.27 | 4.54 | 0.17 | 5.19E-155 | 0.1894 | 713.20 |
| rs4410790 | C | T | 0.63 | 3.94 | 0.15 | 5.59E-141 | 0.1832 | 689.93 |
| rs1057868 | T | C | 0.29 | 1.97 | 0.16 | 5.26E-33 | 0.0403 | 151.60 |
| rs73073176 | C | T | 0.87 | 2.31 | 0.22 | 5.56E-25 | 0.0293 | 110.25 |
| rs1260326 | C | T | 0.61 | 1.36 | 0.15 | 2.62E-19 | 0.0219 | 82.20 |
| rs34060476 | G | A | 0.13 | 1.89 | 0.22 | 5.06E-18 | 0.0196 | 73.80 |
| rs66723169 | A | C | 0.23 | 1.47 | 0.18 | 9.88E-17 | 0.0177 | 66.69 |
| rs10865548 | G | A | 0.83 | 1.54 | 0.19 | 4.46E-15 | 0.0175 | 65.69 |
| rs2330783 | G | T | 0.99 | 4.53 | 0.63 | 1.57E-12 | 0.0138 | 51.70 |
| rs597045 | A | T | 0.69 | 1.07 | 0.16 | 6.62E-11 | 0.0119 | 44.72 |
| rs574367 | T | G | 0.21 | 1.05 | 0.18 | 8.06E-09 | 0.0091 | 34.03 |
| rs1956218 | G | A | 0.56 | 0.82 | 0.15 | 3.62E-08 | 0.0080 | 29.88 |

**Supplementary table 9.** SNPs associated with educational attainment at the genome-wide level of significance (*P* < 5 x 10^-8^) and clumped for independence at kb = 10,000 and *r^2^* = 0.001

| **SNP** | **EA** | **OA** | **EAF** | **BETA** | **SE** | ***P*-value** | **R^2^ %** | **F** |
| --- | --- | --- | --- | --- | --- | --- | --- | --- |
| rs9859556 | T | G | 0.313 | 0.029 | 0.0015 | 4.61E-82 | 0.0330 | 373.78 |
| rs7029718 | A | G | 0.411 | 0.0244 | 0.0014 | 7.82E-65 | 0.0268 | 303.75 |
| rs1334297 | A | G | 0.738 | 0.0257 | 0.0016 | 2.53E-58 | 0.0228 | 258.00 |
| rs9375188 | T | C | 0.485 | 0.0212 | 0.0014 | 8.78E-52 | 0.0203 | 229.31 |
| rs13018640 | T | C | 0.602 | -0.0215 | 0.0014 | 1.05E-50 | 0.0208 | 235.84 |
| rs34305371 | A | G | 0.099 | 0.0314 | 0.0024 | 1.10E-39 | 0.0151 | 171.17 |
| rs6449503 | A | G | 0.504 | 0.0185 | 0.0014 | 1.84E-39 | 0.0154 | 174.62 |
| rs1689510 | C | G | 0.332 | 0.0194 | 0.0015 | 1.35E-38 | 0.0148 | 167.27 |
| rs11082011 | T | C | 0.672 | 0.0194 | 0.0015 | 2.06E-38 | 0.0148 | 167.27 |
| rs1008078 | T | C | 0.402 | -0.0182 | 0.0014 | 4.50E-37 | 0.0149 | 169.00 |
| rs61104616 | A | G | 0.525 | -0.0172 | 0.0014 | 1.56E-34 | 0.0133 | 150.94 |
| rs12076635 | C | G | 0.781 | 0.0207 | 0.0017 | 3.33E-34 | 0.0131 | 148.27 |
| rs66568921 | T | G | 0.644 | -0.0181 | 0.0015 | 7.60E-34 | 0.0129 | 145.60 |
| rs56319902 | T | C | 0.216 | -0.0206 | 0.0017 | 5.81E-33 | 0.0130 | 146.84 |
| rs73344830 | A | G | 0.418 | 0.017 | 0.0014 | 1.00E-32 | 0.0130 | 147.45 |
| rs11678980 | A | G | 0.453 | -0.0166 | 0.0014 | 1.60E-31 | 0.0124 | 140.59 |
| rs11588857 | A | G | 0.213 | 0.0199 | 0.0017 | 4.45E-31 | 0.0121 | 137.03 |
| rs10773002 | A | T | 0.252 | 0.0185 | 0.0016 | 2.50E-30 | 0.0118 | 133.69 |
| rs176218 | T | G | 0.196 | 0.02 | 0.0018 | 1.10E-29 | 0.0109 | 123.46 |
| rs7849487 | T | G | 0.658 | -0.0167 | 0.0015 | 1.25E-29 | 0.0109 | 123.95 |
| rs10189857 | A | G | 0.565 | 0.0158 | 0.0014 | 4.69E-29 | 0.0113 | 127.37 |
| rs4787457 | A | G | 0.634 | 0.0162 | 0.0015 | 1.23E-28 | 0.0103 | 116.64 |
| rs35417702 | T | C | 0.524 | -0.0152 | 0.0014 | 2.40E-27 | 0.0104 | 117.88 |
| rs55736314 | C | G | 0.599 | -0.0154 | 0.0014 | 4.59E-27 | 0.0107 | 121.00 |
| rs10215082 | A | G | 0.466 | -0.0149 | 0.0014 | 7.03E-26 | 0.0100 | 113.27 |
| rs1320139 | C | G | 0.427 | -0.0149 | 0.0014 | 7.62E-26 | 0.0100 | 113.27 |
| rs3897821 | A | G | 0.665 | 0.0154 | 0.0015 | 3.58E-25 | 0.0093 | 105.40 |
| rs6557171 | T | C | 0.323 | -0.0155 | 0.0015 | 5.48E-25 | 0.0094 | 106.78 |
| rs13240401 | T | C | 0.781 | 0.0176 | 0.0017 | 2.36E-24 | 0.0095 | 107.18 |
| rs363096 | T | C | 0.425 | -0.0143 | 0.0014 | 5.77E-24 | 0.0092 | 104.33 |
| rs10931821 | A | T | 0.485 | -0.0141 | 0.0014 | 8.65E-24 | 0.0090 | 101.43 |
| rs76076331 | T | C | 0.135 | 0.0206 | 0.0021 | 1.71E-23 | 0.0085 | 96.23 |
| rs17411339 | A | G | 0.561 | 0.014 | 0.0014 | 5.10E-23 | 0.0088 | 100.00 |
| rs7683416 | T | C | 0.459 | 0.0139 | 0.0014 | 6.05E-23 | 0.0087 | 98.58 |
| rs113520408 | A | G | 0.276 | 0.0155 | 0.0016 | 1.03E-22 | 0.0083 | 93.85 |
| rs12375949 | T | C | 0.431 | -0.0138 | 0.0014 | 1.61E-22 | 0.0086 | 97.16 |
| rs11157931 | A | C | 0.392 | -0.0139 | 0.0014 | 3.86E-22 | 0.0087 | 98.58 |
| rs9411331 | A | C | 0.684 | 0.0145 | 0.0015 | 7.63E-22 | 0.0083 | 93.44 |
| rs660001 | A | G | 0.211 | -0.0164 | 0.0017 | 1.66E-21 | 0.0082 | 93.07 |
| rs7924036 | T | G | 0.512 | 0.0133 | 0.0014 | 2.55E-21 | 0.0080 | 90.25 |
| rs12468040 | T | G | 0.382 | 0.0137 | 0.0014 | 3.12E-21 | 0.0085 | 95.76 |
| rs746839 | C | G | 0.628 | 0.0135 | 0.0015 | 1.19E-20 | 0.0072 | 81.00 |
| rs9616947 | T | C | 0.382 | -0.0134 | 0.0015 | 3.58E-20 | 0.0071 | 79.80 |
| rs12506221 | T | G | 0.436 | -0.013 | 0.0014 | 4.23E-20 | 0.0076 | 86.22 |
| rs10875121 | C | G | 0.836 | 0.0174 | 0.0019 | 4.46E-20 | 0.0074 | 83.87 |
| rs12761761 | T | C | 0.240 | 0.0153 | 0.0017 | 4.79E-20 | 0.0072 | 81.00 |
| rs2725370 | T | C | 0.302 | -0.0141 | 0.0015 | 4.96E-20 | 0.0078 | 88.36 |
| rs56391344 | A | G | 0.253 | 0.0148 | 0.0016 | 7.49E-20 | 0.0076 | 85.56 |
| rs72962169 | T | C | 0.160 | -0.0174 | 0.0019 | 1.26E-19 | 0.0074 | 83.87 |
| rs2838006 | T | C | 0.360 | 0.0132 | 0.0015 | 1.38E-19 | 0.0068 | 77.44 |
| rs72828517 | T | C | 0.827 | -0.0168 | 0.0019 | 1.38E-19 | 0.0069 | 78.18 |
| rs72829857 | A | G | 0.761 | -0.0149 | 0.0016 | 1.52E-19 | 0.0077 | 86.72 |
| rs2179152 | T | C | 0.367 | -0.0131 | 0.0015 | 1.68E-19 | 0.0067 | 76.27 |
| rs79265434 | A | G | 0.883 | -0.0197 | 0.0022 | 1.98E-19 | 0.0071 | 80.18 |
| rs62182994 | T | C | 0.689 | 0.0137 | 0.0015 | 2.55E-19 | 0.0074 | 83.42 |
| rs2290601 | T | C | 0.772 | -0.0149 | 0.0017 | 4.49E-19 | 0.0068 | 76.82 |
| rs178183 | T | C | 0.246 | 0.0145 | 0.0016 | 4.81E-19 | 0.0073 | 82.13 |
| rs6065080 | T | C | 0.359 | -0.013 | 0.0015 | 7.49E-19 | 0.0066 | 75.11 |
| rs2545795 | A | C | 0.444 | 0.0125 | 0.0014 | 1.03E-18 | 0.0070 | 79.72 |
| rs4757957 | C | G | 0.691 | 0.0133 | 0.0015 | 1.79E-18 | 0.0069 | 78.62 |
| rs57352738 | A | T | 0.204 | -0.0152 | 0.0017 | 2.51E-18 | 0.0071 | 79.94 |
| rs10460095 | A | G | 0.571 | -0.0124 | 0.0014 | 2.61E-18 | 0.0069 | 78.45 |
| rs790647 | A | C | 0.228 | -0.0145 | 0.0017 | 3.81E-18 | 0.0064 | 72.75 |
| rs575113 | A | G | 0.294 | 0.0133 | 0.0015 | 7.52E-18 | 0.0069 | 78.62 |
| rs76577427 | C | G | 0.901 | 0.0203 | 0.0024 | 7.78E-18 | 0.0063 | 71.54 |
| rs4719944 | T | C | 0.546 | -0.0121 | 0.0014 | 9.25E-18 | 0.0066 | 74.70 |
| rs9289300 | T | C | 0.841 | -0.0164 | 0.0019 | 1.00E-17 | 0.0066 | 74.50 |
| rs7920624 | A | T | 0.478 | 0.012 | 0.0014 | 1.06E-17 | 0.0065 | 73.47 |
| rs12643771 | T | C | 0.309 | 0.013 | 0.0015 | 1.32E-17 | 0.0066 | 75.11 |
| rs4726070 | A | G | 0.602 | 0.0122 | 0.0014 | 1.36E-17 | 0.0067 | 75.94 |
| rs17563464 | A | C | 0.217 | -0.0147 | 0.0017 | 1.74E-17 | 0.0066 | 74.77 |
| rs717996 | T | C | 0.599 | -0.0122 | 0.0014 | 1.89E-17 | 0.0067 | 75.94 |
| rs17502934 | T | G | 0.149 | -0.0167 | 0.002 | 2.12E-17 | 0.0062 | 69.72 |
| rs9882532 | T | C | 0.637 | 0.0124 | 0.0015 | 2.15E-17 | 0.0060 | 68.34 |
| rs6493265 | T | C | 0.392 | -0.0122 | 0.0014 | 2.26E-17 | 0.0067 | 75.94 |
| rs17489649 | A | G | 0.679 | 0.0127 | 0.0015 | 2.77E-17 | 0.0063 | 71.68 |
| rs76878669 | C | G | 0.761 | 0.0141 | 0.0017 | 3.10E-17 | 0.0061 | 68.79 |
| rs4467547 | T | G | 0.407 | 0.0121 | 0.0014 | 4.88E-17 | 0.0066 | 74.70 |
| rs3026996 | A | C | 0.761 | 0.0138 | 0.0016 | 5.42E-17 | 0.0066 | 74.39 |
| rs9933256 | A | G | 0.556 | 0.0119 | 0.0014 | 6.06E-17 | 0.0064 | 72.25 |
| rs2923424 | A | G | 0.607 | 0.012 | 0.0014 | 6.31E-17 | 0.0065 | 73.47 |
| rs17428076 | C | G | 0.760 | 0.0137 | 0.0016 | 6.41E-17 | 0.0065 | 73.32 |
| rs7977614 | A | G | 0.714 | -0.0134 | 0.0016 | 6.84E-17 | 0.0062 | 70.14 |
| rs77609760 | A | G | 0.935 | 0.0236 | 0.0028 | 7.71E-17 | 0.0063 | 71.04 |
| rs12591647 | T | C | 0.817 | -0.0152 | 0.0018 | 7.78E-17 | 0.0063 | 71.31 |
| rs12957463 | A | G | 0.795 | 0.0146 | 0.0018 | 9.20E-17 | 0.0058 | 65.79 |
| rs2496482 | T | C | 0.416 | 0.0121 | 0.0015 | 9.20E-17 | 0.0057 | 65.07 |
| rs11663602 | A | C | 0.276 | -0.013 | 0.0016 | 9.27E-17 | 0.0058 | 66.02 |
| rs75177132 | T | C | 0.048 | 0.0298 | 0.0036 | 1.01E-16 | 0.0061 | 68.52 |
| rs2081652 | A | T | 0.661 | 0.0123 | 0.0015 | 1.09E-16 | 0.0059 | 67.24 |
| rs7974852 | A | C | 0.524 | 0.0117 | 0.0014 | 1.14E-16 | 0.0062 | 69.84 |
| rs2570497 | T | C | 0.637 | -0.0121 | 0.0015 | 1.27E-16 | 0.0057 | 65.07 |
| rs17598675 | T | C | 0.513 | -0.0116 | 0.0014 | 1.37E-16 | 0.0061 | 68.65 |
| rs11121177 | A | G | 0.179 | 0.0151 | 0.0018 | 1.44E-16 | 0.0062 | 70.37 |
| rs6704768 | A | G | 0.565 | -0.0116 | 0.0014 | 1.84E-16 | 0.0061 | 68.65 |
| rs34098770 | A | G | 0.152 | -0.016 | 0.002 | 2.95E-16 | 0.0057 | 64.00 |
| rs4984541 | A | G | 0.767 | -0.0138 | 0.0017 | 3.40E-16 | 0.0058 | 65.90 |
| rs7326331 | A | G | 0.276 | -0.0128 | 0.0016 | 3.42E-16 | 0.0057 | 64.00 |
| rs9655780 | A | G | 0.829 | -0.0153 | 0.0019 | 3.70E-16 | 0.0057 | 64.84 |
| rs737945 | C | G | 0.444 | -0.0115 | 0.0014 | 3.73E-16 | 0.0060 | 67.47 |
| rs12646523 | T | C | 0.248 | -0.0132 | 0.0016 | 3.79E-16 | 0.0060 | 68.06 |
| rs11081529 | T | C | 0.711 | 0.0126 | 0.0015 | 5.22E-16 | 0.0062 | 70.56 |
| rs73581580 | A | G | 0.125 | -0.0181 | 0.0022 | 5.78E-16 | 0.0060 | 67.69 |
| rs5754753 | T | C | 0.718 | -0.0126 | 0.0016 | 6.15E-16 | 0.0055 | 62.02 |
| rs6690195 | T | C | 0.491 | -0.0113 | 0.0014 | 7.89E-16 | 0.0058 | 65.15 |
| rs115000530 | A | T | 0.945 | -0.025 | 0.0031 | 1.09E-15 | 0.0057 | 65.04 |
| rs10979613 | T | C | 0.639 | -0.0117 | 0.0015 | 1.25E-15 | 0.0054 | 60.84 |
| rs17248751 | A | G | 0.783 | -0.0136 | 0.0017 | 1.28E-15 | 0.0057 | 64.00 |
| rs2998299 | T | G | 0.214 | -0.0136 | 0.0017 | 1.51E-15 | 0.0057 | 64.00 |
| rs62379838 | T | C | 0.695 | 0.0121 | 0.0015 | 1.77E-15 | 0.0057 | 65.07 |
| rs9556958 | T | C | 0.523 | -0.011 | 0.0014 | 5.96E-15 | 0.0055 | 61.73 |
| rs13422673 | T | C | 0.466 | -0.011 | 0.0014 | 6.48E-15 | 0.0055 | 61.73 |
| rs2052285 | A | G | 0.596 | 0.0112 | 0.0014 | 7.25E-15 | 0.0057 | 64.00 |
| rs488476 | C | G | 0.619 | -0.0113 | 0.0015 | 8.29E-15 | 0.0050 | 56.75 |
| rs2764684 | T | C | 0.827 | 0.0143 | 0.0019 | 9.99E-15 | 0.0050 | 56.65 |
| rs10892807 | T | C | 0.567 | 0.0109 | 0.0014 | 1.09E-14 | 0.0054 | 60.62 |
| rs61527214 | A | G | 0.411 | 0.011 | 0.0014 | 1.13E-14 | 0.0055 | 61.73 |
| rs8024 | A | C | 0.329 | -0.0115 | 0.0015 | 1.13E-14 | 0.0052 | 58.78 |
| rs7603132 | A | G | 0.194 | 0.0137 | 0.0018 | 1.20E-14 | 0.0051 | 57.93 |
| rs10940921 | T | G | 0.419 | 0.0111 | 0.0014 | 1.31E-14 | 0.0056 | 62.86 |
| rs6731373 | A | G | 0.339 | -0.0115 | 0.0015 | 1.39E-14 | 0.0052 | 58.78 |
| rs303752 | A | G | 0.407 | -0.011 | 0.0014 | 1.47E-14 | 0.0055 | 61.73 |
| rs7254263 | T | C | 0.285 | -0.0119 | 0.0016 | 1.84E-14 | 0.0049 | 55.32 |
| rs4320563 | A | G | 0.509 | 0.0109 | 0.0014 | 2.24E-14 | 0.0054 | 60.62 |
| rs11871429 | A | G | 0.773 | 0.0128 | 0.0017 | 2.52E-14 | 0.0050 | 56.69 |
| rs10761251 | A | T | 0.663 | 0.0113 | 0.0015 | 2.52E-14 | 0.0050 | 56.75 |
| rs1866823 | A | G | 0.544 | 0.0107 | 0.0014 | 3.09E-14 | 0.0052 | 58.41 |
| rs7575637 | A | G | 0.454 | 0.0107 | 0.0014 | 3.43E-14 | 0.0052 | 58.41 |
| rs9529146 | T | C | 0.228 | 0.0127 | 0.0017 | 3.48E-14 | 0.0049 | 55.81 |
| rs4848924 | A | C | 0.710 | -0.0117 | 0.0015 | 3.56E-14 | 0.0054 | 60.84 |
| rs1747714 | T | C | 0.529 | 0.0106 | 0.0014 | 3.82E-14 | 0.0051 | 57.33 |
| rs929511 | T | C | 0.125 | -0.016 | 0.0021 | 3.91E-14 | 0.0051 | 58.05 |
| rs12602286 | T | G | 0.873 | 0.016 | 0.0021 | 4.62E-14 | 0.0051 | 58.05 |
| rs13163845 | T | C | 0.849 | -0.0149 | 0.002 | 4.66E-14 | 0.0049 | 55.50 |
| rs12273435 | A | G | 0.207 | -0.0132 | 0.0017 | 4.72E-14 | 0.0053 | 60.29 |
| rs10798888 | T | G | 0.173 | -0.014 | 0.0019 | 5.15E-14 | 0.0048 | 54.29 |
| rs1128956 | T | G | 0.825 | -0.014 | 0.0019 | 5.34E-14 | 0.0048 | 54.29 |
| rs17565975 | A | G | 0.556 | -0.0106 | 0.0014 | 5.54E-14 | 0.0051 | 57.33 |
| rs12332731 | A | T | 0.188 | 0.0135 | 0.0018 | 5.96E-14 | 0.0050 | 56.25 |
| rs7972246 | T | C | 0.345 | 0.011 | 0.0015 | 1.01E-13 | 0.0048 | 53.78 |
| rs11620355 | A | G | 0.091 | 0.0182 | 0.0025 | 1.09E-13 | 0.0047 | 53.00 |
| rs401526 | T | C | 0.526 | -0.0104 | 0.0014 | 1.15E-13 | 0.0049 | 55.18 |
| rs2287838 | A | G | 0.550 | -0.0104 | 0.0014 | 1.27E-13 | 0.0049 | 55.18 |
| rs6697584 | T | C | 0.785 | -0.0126 | 0.0017 | 1.43E-13 | 0.0049 | 54.93 |
| rs12571549 | A | G | 0.137 | 0.0151 | 0.002 | 1.45E-13 | 0.0050 | 57.00 |
| rs118134876 | T | C | 0.058 | -0.0223 | 0.003 | 1.50E-13 | 0.0049 | 55.25 |
| rs17551064 | A | G | 0.838 | 0.0141 | 0.0019 | 1.53E-13 | 0.0049 | 55.07 |
| rs9853928 | T | C | 0.210 | -0.0127 | 0.0017 | 1.59E-13 | 0.0049 | 55.81 |
| rs4500930 | T | C | 0.345 | -0.0109 | 0.0015 | 1.67E-13 | 0.0047 | 52.80 |
| rs4352658 | T | C | 0.081 | -0.019 | 0.0026 | 1.71E-13 | 0.0047 | 53.40 |
| rs12875339 | A | C | 0.645 | -0.0108 | 0.0015 | 1.71E-13 | 0.0046 | 51.84 |
| rs10060023 | T | C | 0.328 | 0.011 | 0.0015 | 1.88E-13 | 0.0048 | 53.78 |
| rs34720381 | T | C | 0.091 | -0.018 | 0.0024 | 1.90E-13 | 0.0050 | 56.25 |
| rs7460106 | T | C | 0.762 | -0.0123 | 0.0017 | 2.06E-13 | 0.0046 | 52.35 |
| rs10805383 | A | G | 0.488 | -0.0103 | 0.0014 | 2.22E-13 | 0.0048 | 54.13 |
| rs61997667 | T | C | 0.151 | -0.0144 | 0.002 | 2.24E-13 | 0.0046 | 51.84 |
| rs11657342 | A | G | 0.367 | 0.014 | 0.0019 | 2.32E-13 | 0.0048 | 54.29 |
| rs13085461 | C | G | 0.474 | 0.0103 | 0.0014 | 2.40E-13 | 0.0048 | 54.13 |
| rs2256965 | A | G | 0.419 | 0.0106 | 0.0014 | 2.40E-13 | 0.0051 | 57.33 |
| rs11542663 | A | C | 0.695 | 0.0112 | 0.0015 | 2.44E-13 | 0.0049 | 55.75 |
| rs2478208 | C | G | 0.520 | -0.0103 | 0.0014 | 2.89E-13 | 0.0048 | 54.13 |
| rs2364544 | A | G | 0.391 | -0.0105 | 0.0014 | 2.91E-13 | 0.0050 | 56.25 |
| rs1167827 | A | G | 0.435 | 0.0104 | 0.0014 | 2.93E-13 | 0.0049 | 55.18 |
| rs56099375 | T | C | 0.241 | 0.012 | 0.0017 | 3.10E-13 | 0.0044 | 49.83 |
| rs17048855 | A | G | 0.343 | 0.0108 | 0.0015 | 3.10E-13 | 0.0046 | 51.84 |
| rs10951590 | T | C | 0.328 | -0.0109 | 0.0015 | 3.62E-13 | 0.0047 | 52.80 |
| rs12503522 | T | C | 0.282 | -0.0113 | 0.0016 | 3.93E-13 | 0.0044 | 49.88 |
| rs9886703 | A | T | 0.167 | -0.0136 | 0.0019 | 4.46E-13 | 0.0045 | 51.24 |
| rs77025239 | A | G | 0.156 | -0.014 | 0.0019 | 4.65E-13 | 0.0048 | 54.29 |
| rs111852224 | T | C | 0.123 | 0.0156 | 0.0022 | 4.85E-13 | 0.0044 | 50.28 |
| rs4384309 | A | G | 0.464 | 0.0102 | 0.0014 | 4.89E-13 | 0.0047 | 53.08 |
| rs12524795 | T | C | 0.437 | 0.0102 | 0.0014 | 5.28E-13 | 0.0047 | 53.08 |
| rs72622559 | T | C | 0.230 | -0.012 | 0.0017 | 5.50E-13 | 0.0044 | 49.83 |
| rs62179650 | A | G | 0.300 | 0.0112 | 0.0016 | 5.66E-13 | 0.0043 | 49.00 |
| rs6065784 | C | G | 0.695 | 0.011 | 0.0015 | 5.78E-13 | 0.0048 | 53.78 |
| rs4673840 | T | C | 0.842 | -0.0138 | 0.0019 | 5.86E-13 | 0.0047 | 52.75 |
| rs3809634 | A | G | 0.684 | -0.0109 | 0.0015 | 6.82E-13 | 0.0047 | 52.80 |
| rs4899012 | C | G | 0.602 | -0.0103 | 0.0014 | 8.06E-13 | 0.0048 | 54.13 |
| rs730384 | A | G | 0.439 | 0.0101 | 0.0014 | 9.38E-13 | 0.0046 | 52.05 |
| rs34067381 | T | G | 0.362 | -0.0104 | 0.0015 | 9.77E-13 | 0.0042 | 48.07 |
| rs969512 | A | T | 0.660 | -0.0106 | 0.0015 | 9.84E-13 | 0.0044 | 49.94 |
| rs6994287 | A | G | 0.406 | 0.0102 | 0.0014 | 1.11E-12 | 0.0047 | 53.08 |
| rs62155873 | T | C | 0.122 | -0.0151 | 0.0021 | 1.65E-12 | 0.0046 | 51.70 |
| rs114593137 | A | T | 0.793 | -0.0122 | 0.0017 | 1.65E-12 | 0.0045 | 51.50 |
| rs35929923 | A | G | 0.250 | -0.0114 | 0.0016 | 1.77E-12 | 0.0045 | 50.77 |
| rs891793 | C | G | 0.540 | -0.0099 | 0.0014 | 1.87E-12 | 0.0044 | 50.01 |
| rs73648455 | T | C | 0.080 | -0.0183 | 0.0026 | 1.99E-12 | 0.0044 | 49.54 |
| rs192436652 | T | C | 0.027 | -0.0319 | 0.0045 | 2.06E-12 | 0.0044 | 50.25 |
| rs175325 | A | T | 0.597 | -0.01 | 0.0014 | 2.24E-12 | 0.0045 | 51.02 |
| rs9929556 | T | G | 0.569 | -0.0099 | 0.0014 | 2.31E-12 | 0.0044 | 50.01 |
| rs17604349 | A | G | 0.185 | 0.0127 | 0.0018 | 2.36E-12 | 0.0044 | 49.78 |
| rs2216144 | T | C | 0.531 | 0.0098 | 0.0014 | 2.58E-12 | 0.0043 | 49.00 |
| rs13197257 | T | G | 0.279 | 0.0109 | 0.0016 | 2.83E-12 | 0.0041 | 46.41 |
| rs6969783 | A | T | 0.471 | -0.0098 | 0.0014 | 2.87E-12 | 0.0043 | 49.00 |
| rs4846724 | A | G | 0.535 | 0.0098 | 0.0014 | 2.89E-12 | 0.0043 | 49.00 |
| rs12638072 | A | G | 0.299 | -0.0107 | 0.0015 | 3.13E-12 | 0.0045 | 50.88 |
| rs1245829 | A | T | 0.581 | -0.0099 | 0.0014 | 3.29E-12 | 0.0044 | 50.01 |
| rs77719387 | A | T | 0.017 | -0.0403 | 0.0058 | 3.78E-12 | 0.0043 | 48.28 |
| rs8008382 | T | C | 0.301 | -0.0106 | 0.0015 | 4.07E-12 | 0.0044 | 49.94 |
| rs7617204 | A | G | 0.522 | -0.0097 | 0.0014 | 4.33E-12 | 0.0042 | 48.01 |
| rs7041702 | A | G | 0.741 | -0.0111 | 0.0016 | 4.53E-12 | 0.0043 | 48.13 |
| rs1434630 | T | G | 0.148 | -0.0137 | 0.002 | 4.63E-12 | 0.0041 | 46.92 |
| rs4839155 | T | G | 0.767 | 0.0115 | 0.0017 | 4.66E-12 | 0.0040 | 45.76 |
| rs7910403 | T | G | 0.195 | 0.0122 | 0.0018 | 5.08E-12 | 0.0041 | 45.94 |
| rs7040995 | C | G | 0.532 | 0.0097 | 0.0014 | 5.39E-12 | 0.0042 | 48.01 |
| rs9373363 | A | G | 0.749 | -0.0111 | 0.0016 | 5.54E-12 | 0.0043 | 48.13 |
| rs79994730 | T | C | 0.043 | 0.024 | 0.0035 | 5.88E-12 | 0.0042 | 47.02 |
| rs74415461 | T | C | 0.084 | 0.0176 | 0.0026 | 6.25E-12 | 0.0040 | 45.82 |
| rs13133213 | A | G | 0.498 | 0.0096 | 0.0014 | 7.57E-12 | 0.0042 | 47.02 |
| rs10773208 | T | C | 0.253 | -0.011 | 0.0016 | 8.09E-12 | 0.0042 | 47.27 |
| rs13145650 | T | C | 0.917 | -0.0173 | 0.0025 | 8.99E-12 | 0.0042 | 47.89 |
| rs12888615 | T | C | 0.199 | 0.012 | 0.0018 | 9.42E-12 | 0.0039 | 44.44 |
| rs1842713 | A | G | 0.788 | -0.0117 | 0.0017 | 9.67E-12 | 0.0042 | 47.37 |
| rs3859523 | T | C | 0.821 | 0.0124 | 0.0018 | 9.92E-12 | 0.0042 | 47.46 |
| rs9929762 | A | G | 0.560 | 0.0096 | 0.0014 | 1.03E-11 | 0.0042 | 47.02 |
| rs4298514 | T | C | 0.699 | 0.0104 | 0.0015 | 1.07E-11 | 0.0042 | 48.07 |
| rs2447097 | T | G | 0.460 | 0.0096 | 0.0014 | 1.10E-11 | 0.0042 | 47.02 |
| rs4358081 | A | C | 0.529 | -0.0095 | 0.0014 | 1.28E-11 | 0.0041 | 46.05 |
| rs12967010 | T | C | 0.775 | 0.0114 | 0.0017 | 1.33E-11 | 0.0040 | 44.97 |
| rs75033012 | C | G | 0.039 | -0.0258 | 0.0038 | 1.38E-11 | 0.0041 | 46.10 |
| rs1426619 | T | C | 0.435 | 0.0096 | 0.0014 | 1.40E-11 | 0.0042 | 47.02 |
| rs7167688 | T | C | 0.492 | -0.0094 | 0.0014 | 1.71E-11 | 0.0040 | 45.08 |
| rs401966 | C | G | 0.390 | -0.0097 | 0.0014 | 1.72E-11 | 0.0042 | 48.01 |
| rs3751331 | A | G | 0.380 | -0.0097 | 0.0014 | 1.75E-11 | 0.0042 | 48.01 |
| rs79728014 | A | G | 0.855 | -0.0134 | 0.002 | 2.06E-11 | 0.0040 | 44.89 |
| rs11138947 | T | C | 0.724 | 0.0105 | 0.0016 | 2.36E-11 | 0.0038 | 43.07 |
| rs590013 | T | C | 0.677 | 0.01 | 0.0015 | 2.47E-11 | 0.0039 | 44.44 |
| rs4766424 | C | G | 0.124 | 0.0142 | 0.0021 | 2.47E-11 | 0.0040 | 45.72 |
| rs4328757 | T | C | 0.612 | 0.0096 | 0.0014 | 2.95E-11 | 0.0042 | 47.02 |
| rs6123924 | A | G | 0.845 | 0.0129 | 0.0019 | 3.20E-11 | 0.0041 | 46.10 |
| rs34807077 | A | C | 0.157 | 0.0128 | 0.0019 | 3.29E-11 | 0.0040 | 45.38 |
| rs12151248 | T | C | 0.114 | -0.0149 | 0.0023 | 3.81E-11 | 0.0037 | 41.97 |
| rs35606437 | A | G | 0.268 | 0.0105 | 0.0016 | 3.98E-11 | 0.0038 | 43.07 |
| rs1865955 | T | C | 0.824 | 0.0122 | 0.0019 | 4.06E-11 | 0.0036 | 41.23 |
| rs13327482 | A | G | 0.822 | -0.0121 | 0.0018 | 4.47E-11 | 0.0040 | 45.19 |
| rs4675248 | A | G | 0.400 | -0.0094 | 0.0014 | 4.50E-11 | 0.0040 | 45.08 |
| rs2964199 | T | C | 0.315 | -0.0099 | 0.0015 | 4.61E-11 | 0.0038 | 43.56 |
| rs6812533 | T | C | 0.744 | 0.0106 | 0.0016 | 4.85E-11 | 0.0039 | 43.89 |
| rs4778058 | T | C | 0.496 | -0.0092 | 0.0014 | 4.92E-11 | 0.0038 | 43.18 |
| rs6924023 | A | G | 0.755 | 0.0107 | 0.0016 | 5.17E-11 | 0.0040 | 44.72 |
| rs10830858 | T | C | 0.510 | 0.0092 | 0.0014 | 5.31E-11 | 0.0038 | 43.18 |
| rs4895650 | T | C | 0.547 | 0.0093 | 0.0014 | 5.31E-11 | 0.0039 | 44.13 |
| rs4369924 | A | G | 0.162 | 0.0125 | 0.0019 | 5.58E-11 | 0.0038 | 43.28 |
| rs12670376 | A | G | 0.446 | 0.0092 | 0.0014 | 5.99E-11 | 0.0038 | 43.18 |
| rs9927842 | T | C | 0.149 | -0.013 | 0.002 | 6.22E-11 | 0.0037 | 42.25 |
| rs1991585 | T | C | 0.706 | -0.0101 | 0.0015 | 6.26E-11 | 0.0040 | 45.34 |
| rs252991 | A | G | 0.370 | 0.0095 | 0.0015 | 6.30E-11 | 0.0035 | 40.11 |
| rs12789313 | T | C | 0.491 | 0.0092 | 0.0014 | 6.79E-11 | 0.0038 | 43.18 |
| rs743316 | T | C | 0.792 | 0.0112 | 0.0017 | 8.21E-11 | 0.0038 | 43.40 |
| rs7321274 | A | G | 0.800 | 0.0114 | 0.0018 | 8.21E-11 | 0.0035 | 40.11 |
| rs1329125 | T | C | 0.327 | -0.0097 | 0.0015 | 8.37E-11 | 0.0037 | 41.82 |
| rs57349798 | A | G | 0.409 | 0.0093 | 0.0014 | 8.63E-11 | 0.0039 | 44.13 |
| rs17144467 | A | C | 0.687 | 0.0098 | 0.0015 | 8.74E-11 | 0.0038 | 42.68 |
| rs2297293 | C | G | 0.314 | 0.0099 | 0.0015 | 8.80E-11 | 0.0038 | 43.56 |
| rs74787922 | A | C | 0.933 | 0.0182 | 0.0028 | 8.97E-11 | 0.0037 | 42.25 |
| rs2434672 | A | C | 0.532 | 0.0091 | 0.0014 | 1.06E-10 | 0.0037 | 42.25 |
| rs12519073 | T | C | 0.232 | -0.0107 | 0.0017 | 1.16E-10 | 0.0035 | 39.62 |
| rs1717204 | A | C | 0.182 | -0.0117 | 0.0018 | 1.20E-10 | 0.0037 | 42.25 |
| rs7012546 | T | C | 0.417 | 0.0092 | 0.0014 | 1.25E-10 | 0.0038 | 43.18 |
| rs1301838 | T | C | 0.323 | -0.0096 | 0.0015 | 1.29E-10 | 0.0036 | 40.96 |
| rs2212430 | T | C | 0.304 | -0.01 | 0.0016 | 1.32E-10 | 0.0035 | 39.06 |
| rs7692359 | T | C | 0.779 | -0.0109 | 0.0017 | 1.40E-10 | 0.0036 | 41.11 |
| rs1738050 | C | G | 0.620 | -0.0093 | 0.0014 | 1.42E-10 | 0.0039 | 44.13 |
| rs17110109 | T | C | 0.616 | -0.0093 | 0.0014 | 1.47E-10 | 0.0039 | 44.13 |
| rs12438177 | A | G | 0.368 | 0.0093 | 0.0015 | 1.48E-10 | 0.0034 | 38.44 |
| rs9359939 | A | C | 0.243 | -0.0105 | 0.0016 | 1.51E-10 | 0.0038 | 43.07 |
| rs72486027 | T | C | 0.246 | -0.0104 | 0.0016 | 1.61E-10 | 0.0037 | 42.25 |
| rs62155350 | A | G | 0.019 | 0.0338 | 0.0053 | 1.69E-10 | 0.0036 | 40.67 |
| rs10772644 | C | G | 0.889 | 0.0142 | 0.0022 | 1.69E-10 | 0.0037 | 41.66 |
| rs56794817 | A | G | 0.162 | 0.0122 | 0.0019 | 1.69E-10 | 0.0036 | 41.23 |
| rs112806496 | C | G | 0.913 | -0.016 | 0.0025 | 1.72E-10 | 0.0036 | 40.96 |
| rs13130765 | C | G | 0.472 | -0.0091 | 0.0014 | 1.89E-10 | 0.0037 | 42.25 |
| rs73191311 | A | G | 0.336 | -0.0094 | 0.0015 | 2.03E-10 | 0.0035 | 39.27 |
| rs11894424 | A | C | 0.952 | 0.0209 | 0.0033 | 2.14E-10 | 0.0035 | 40.11 |
| rs1603460 | T | G | 0.421 | 0.009 | 0.0014 | 2.20E-10 | 0.0037 | 41.33 |
| rs42210 | C | G | 0.712 | -0.0098 | 0.0016 | 2.74E-10 | 0.0033 | 37.52 |
| rs7650602 | T | C | 0.560 | -0.0089 | 0.0014 | 2.77E-10 | 0.0036 | 40.41 |
| rs137079 | T | C | 0.138 | 0.0128 | 0.002 | 3.06E-10 | 0.0036 | 40.96 |
| rs13261773 | C | G | 0.160 | 0.0122 | 0.0019 | 3.12E-10 | 0.0036 | 41.23 |
| rs80257979 | T | G | 0.968 | -0.0252 | 0.004 | 3.31E-10 | 0.0035 | 39.69 |
| rs3111251 | T | C | 0.433 | -0.009 | 0.0014 | 3.88E-10 | 0.0037 | 41.33 |
| rs3948495 | T | G | 0.408 | -0.0089 | 0.0014 | 3.95E-10 | 0.0036 | 40.41 |
| rs4663617 | A | T | 0.235 | 0.0104 | 0.0017 | 4.12E-10 | 0.0033 | 37.43 |
| rs11130380 | T | G | 0.614 | 0.009 | 0.0014 | 4.17E-10 | 0.0037 | 41.33 |
| rs76235882 | A | G | 0.966 | 0.0241 | 0.0039 | 4.52E-10 | 0.0034 | 38.19 |
| rs11030102 | C | G | 0.748 | 0.0101 | 0.0016 | 4.60E-10 | 0.0035 | 39.85 |
| rs2885198 | A | G | 0.532 | 0.0088 | 0.0014 | 4.63E-10 | 0.0035 | 39.51 |
| rs580652 | T | C | 0.902 | -0.0147 | 0.0024 | 4.65E-10 | 0.0033 | 37.52 |
| rs806816 | A | T | 0.750 | 0.0101 | 0.0016 | 4.80E-10 | 0.0035 | 39.85 |
| rs10844179 | A | G | 0.226 | 0.0104 | 0.0017 | 5.03E-10 | 0.0033 | 37.43 |
| rs11023764 | A | G | 0.642 | 0.0091 | 0.0015 | 5.32E-10 | 0.0033 | 36.80 |
| rs11003463 | T | G | 0.611 | 0.0091 | 0.0015 | 5.58E-10 | 0.0033 | 36.80 |
| rs1933264 | T | C | 0.749 | 0.01 | 0.0016 | 5.85E-10 | 0.0035 | 39.06 |
| rs76246107 | A | G | 0.094 | -0.0162 | 0.0026 | 6.52E-10 | 0.0034 | 38.82 |
| rs10080647 | A | C | 0.141 | 0.0124 | 0.002 | 7.05E-10 | 0.0034 | 38.44 |
| rs232496 | T | C | 0.366 | 0.009 | 0.0015 | 7.14E-10 | 0.0032 | 36.00 |
| rs68145588 | T | G | 0.140 | -0.0127 | 0.0021 | 7.53E-10 | 0.0032 | 36.57 |
| rs7171405 | A | G | 0.243 | -0.0101 | 0.0016 | 7.53E-10 | 0.0035 | 39.85 |
| rs11211123 | A | G | 0.223 | 0.0104 | 0.0017 | 7.62E-10 | 0.0033 | 37.43 |
| rs2517086 | C | G | 0.572 | -0.0088 | 0.0014 | 8.00E-10 | 0.0035 | 39.51 |
| rs2007655 | T | G | 0.498 | 0.0086 | 0.0014 | 8.64E-10 | 0.0033 | 37.73 |
| rs2554835 | A | G | 0.400 | 0.0088 | 0.0014 | 8.95E-10 | 0.0035 | 39.51 |
| rs72944064 | T | C | 0.744 | -0.0098 | 0.0016 | 9.39E-10 | 0.0033 | 37.52 |
| rs10021733 | T | C | 0.148 | -0.0164 | 0.0027 | 9.45E-10 | 0.0033 | 36.89 |
| rs7449561 | A | G | 0.226 | 0.0102 | 0.0017 | 9.73E-10 | 0.0032 | 36.00 |
| rs11598765 | A | G | 0.197 | -0.0106 | 0.0018 | 1.73E-09 | 0.0031 | 34.68 |
| rs1544 | A | G | 0.264 | -0.0096 | 0.0016 | 1.76E-09 | 0.0032 | 36.00 |
| rs28513882 | A | G | 0.178 | -0.011 | 0.0018 | 2.03E-09 | 0.0033 | 37.35 |
| rs11724690 | T | G | 0.289 | 0.0093 | 0.0015 | 2.08E-09 | 0.0034 | 38.44 |
| rs34394051 | A | G | 0.843 | -0.0117 | 0.002 | 2.30E-09 | 0.0030 | 34.22 |
| rs12054166 | C | G | 0.740 | 0.0096 | 0.0016 | 2.30E-09 | 0.0032 | 36.00 |
| rs8097125 | T | C | 0.380 | 0.0086 | 0.0014 | 2.31E-09 | 0.0033 | 37.73 |
| rs1564347 | T | G | 0.343 | 0.0088 | 0.0015 | 2.39E-09 | 0.0030 | 34.42 |
| rs736281 | T | C | 0.393 | 0.0086 | 0.0014 | 2.49E-09 | 0.0033 | 37.73 |
| rs12113634 | T | C | 0.650 | 0.0088 | 0.0015 | 2.50E-09 | 0.0030 | 34.42 |
| rs6917154 | T | C | 0.124 | 0.0127 | 0.0021 | 2.55E-09 | 0.0032 | 36.57 |
| rs2034631 | T | C | 0.651 | 0.0089 | 0.0015 | 2.64E-09 | 0.0031 | 35.20 |
| rs1542354 | A | G | 0.476 | -0.0084 | 0.0014 | 2.72E-09 | 0.0032 | 36.00 |
| rs112603734 | A | C | 0.770 | -0.0131 | 0.0022 | 2.72E-09 | 0.0031 | 35.46 |
| rs12981405 | T | C | 0.166 | -0.0112 | 0.0019 | 2.75E-09 | 0.0031 | 34.75 |
| rs62174974 | A | G | 0.195 | -0.0105 | 0.0018 | 2.76E-09 | 0.0030 | 34.03 |
| rs72917504 | T | C | 0.058 | -0.0178 | 0.003 | 2.91E-09 | 0.0031 | 35.20 |
| rs113615161 | T | C | 0.133 | -0.0123 | 0.0021 | 2.98E-09 | 0.0030 | 34.31 |
| rs17882802 | A | G | 0.430 | 0.0084 | 0.0014 | 3.00E-09 | 0.0032 | 36.00 |
| rs10193498 | A | T | 0.755 | 0.0097 | 0.0016 | 3.10E-09 | 0.0032 | 36.75 |
| rs2199409 | T | C | 0.796 | 0.0103 | 0.0017 | 3.14E-09 | 0.0032 | 36.71 |
| rs12614263 | A | G | 0.496 | 0.0083 | 0.0014 | 3.38E-09 | 0.0031 | 35.15 |
| rs6490618 | T | C | 0.327 | 0.0088 | 0.0015 | 3.48E-09 | 0.0030 | 34.42 |
| rs4810894 | A | G | 0.371 | -0.0085 | 0.0015 | 4.38E-09 | 0.0028 | 32.11 |
| rs10046069 | A | G | 0.120 | 0.0127 | 0.0022 | 4.40E-09 | 0.0029 | 33.32 |
| rs10006235 | T | C | 0.269 | -0.0093 | 0.0016 | 4.48E-09 | 0.0030 | 33.79 |
| rs143743568 | A | G | 0.154 | 0.0118 | 0.002 | 4.99E-09 | 0.0031 | 34.81 |
| rs9927137 | A | G | 0.530 | 0.0082 | 0.0014 | 5.11E-09 | 0.0030 | 34.31 |
| rs2039204 | A | T | 0.530 | 0.0082 | 0.0014 | 5.23E-09 | 0.0030 | 34.31 |
| rs7894722 | T | C | 0.375 | 0.0085 | 0.0014 | 5.41E-09 | 0.0033 | 36.86 |
| rs173003 | A | C | 0.493 | -0.0082 | 0.0014 | 5.50E-09 | 0.0030 | 34.31 |
| rs6977237 | T | C | 0.473 | 0.0082 | 0.0014 | 5.59E-09 | 0.0030 | 34.31 |
| rs162445 | A | G | 0.079 | 0.0152 | 0.0026 | 5.72E-09 | 0.0030 | 34.18 |
| rs42302 | A | G | 0.344 | 0.0086 | 0.0015 | 5.96E-09 | 0.0029 | 32.87 |
| rs912883 | T | C | 0.678 | 0.0087 | 0.0015 | 6.20E-09 | 0.0030 | 33.64 |
| rs4904523 | A | G | 0.521 | -0.0082 | 0.0014 | 6.27E-09 | 0.0030 | 34.31 |
| rs2964255 | A | G | 0.307 | 0.0088 | 0.0015 | 6.34E-09 | 0.0030 | 34.42 |
| rs1890132 | T | C | 0.706 | -0.0089 | 0.0015 | 6.56E-09 | 0.0031 | 35.20 |
| rs1505676 | C | G | 0.629 | 0.0084 | 0.0015 | 6.67E-09 | 0.0028 | 31.36 |
| rs4977885 | A | G | 0.397 | -0.0084 | 0.0014 | 6.78E-09 | 0.0032 | 36.00 |
| rs17148998 | A | G | 0.204 | 0.0101 | 0.0017 | 6.98E-09 | 0.0031 | 35.30 |
| rs13212041 | T | C | 0.798 | 0.0101 | 0.0018 | 7.42E-09 | 0.0028 | 31.48 |
| rs10996167 | C | G | 0.651 | 0.0113 | 0.002 | 7.47E-09 | 0.0028 | 31.92 |
| rs2929032 | A | G | 0.465 | -0.0081 | 0.0014 | 8.40E-09 | 0.0030 | 33.47 |
| rs150537577 | A | G | 0.920 | -0.0151 | 0.0026 | 9.50E-09 | 0.0030 | 33.73 |
| rs4915735 | A | G | 0.141 | 0.0116 | 0.002 | 9.56E-09 | 0.0030 | 33.64 |
| rs6043521 | T | C | 0.604 | -0.0083 | 0.0014 | 9.72E-09 | 0.0031 | 35.15 |
| rs2011603 | A | G | 0.736 | -0.0091 | 0.0016 | 1.12E-08 | 0.0029 | 32.35 |
| rs72993796 | T | C | 0.884 | 0.0127 | 0.0022 | 1.12E-08 | 0.0029 | 33.32 |
| rs117398064 | C | G | 0.091 | -0.0141 | 0.0025 | 1.16E-08 | 0.0028 | 31.81 |
| rs182902112 | A | C | 0.019 | -0.03 | 0.0053 | 1.17E-08 | 0.0028 | 32.04 |
| rs2469226 | A | T | 0.223 | 0.0096 | 0.0017 | 1.28E-08 | 0.0028 | 31.89 |
| rs13402497 | A | T | 0.490 | -0.008 | 0.0014 | 1.33E-08 | 0.0029 | 32.65 |
| rs382196 | T | G | 0.381 | -0.0082 | 0.0014 | 1.47E-08 | 0.0030 | 34.31 |
| rs66721975 | A | G | 0.290 | -0.0087 | 0.0015 | 1.51E-08 | 0.0030 | 33.64 |
| rs2131167 | A | G | 0.446 | 0.008 | 0.0014 | 1.52E-08 | 0.0029 | 32.65 |
| rs1220779 | A | G | 0.468 | -0.0079 | 0.0014 | 1.58E-08 | 0.0028 | 31.84 |
| rs4793090 | A | G | 0.664 | 0.0084 | 0.0015 | 1.67E-08 | 0.0028 | 31.36 |
| rs78648104 | T | C | 0.914 | -0.0141 | 0.0025 | 1.70E-08 | 0.0028 | 31.81 |
| rs13266287 | A | T | 0.669 | -0.0085 | 0.0015 | 1.75E-08 | 0.0028 | 32.11 |
| rs2183271 | T | C | 0.636 | 0.0084 | 0.0015 | 1.91E-08 | 0.0028 | 31.36 |
| rs72709560 | A | G | 0.687 | 0.0085 | 0.0015 | 2.03E-08 | 0.0028 | 32.11 |
| rs1955250 | A | C | 0.914 | -0.014 | 0.0025 | 2.26E-08 | 0.0028 | 31.36 |
| rs1747817 | T | C | 0.761 | -0.0092 | 0.0016 | 2.29E-08 | 0.0029 | 33.06 |
| rs1861786 | A | G | 0.381 | -0.0081 | 0.0014 | 2.39E-08 | 0.0030 | 33.47 |
| rs8030487 | A | G | 0.691 | 0.0085 | 0.0015 | 2.52E-08 | 0.0028 | 32.11 |
| rs11213482 | A | G | 0.836 | -0.0106 | 0.0019 | 2.68E-08 | 0.0027 | 31.12 |
| rs7226824 | T | C | 0.531 | -0.0078 | 0.0014 | 2.72E-08 | 0.0027 | 31.04 |
| rs7875078 | A | C | 0.456 | -0.0078 | 0.0014 | 2.72E-08 | 0.0027 | 31.04 |
| rs9633970 | T | C | 0.761 | 0.0091 | 0.0016 | 2.81E-08 | 0.0029 | 32.35 |
| rs12765185 | A | T | 0.277 | -0.0088 | 0.0016 | 2.96E-08 | 0.0027 | 30.25 |
| rs12574281 | A | C | 0.631 | -0.008 | 0.0015 | 3.04E-08 | 0.0025 | 28.44 |
| rs57661533 | T | C | 0.134 | -0.0114 | 0.0021 | 3.24E-08 | 0.0026 | 29.47 |
| rs2958182 | A | T | 0.341 | 0.0082 | 0.0015 | 3.46E-08 | 0.0026 | 29.88 |
| rs2989476 | C | G | 0.419 | 0.0078 | 0.0014 | 3.73E-08 | 0.0027 | 31.04 |
| rs628993 | A | G | 0.107 | 0.0128 | 0.0023 | 3.83E-08 | 0.0027 | 30.97 |
| rs17732878 | T | C | 0.791 | -0.0095 | 0.0017 | 4.06E-08 | 0.0028 | 31.23 |
| rs10799615 | A | G | 0.253 | -0.0089 | 0.0016 | 4.11E-08 | 0.0027 | 30.94 |
| rs11076962 | T | C | 0.719 | 0.0086 | 0.0016 | 4.29E-08 | 0.0026 | 28.89 |
| rs9492774 | C | G | 0.369 | 0.008 | 0.0015 | 4.33E-08 | 0.0025 | 28.44 |
| rs936496 | A | G | 0.623 | -0.0079 | 0.0014 | 4.38E-08 | 0.0028 | 31.84 |
| rs13050131 | A | C | 0.665 | -0.0081 | 0.0015 | 4.40E-08 | 0.0026 | 29.16 |
| rs2436760 | T | C | 0.972 | 0.0232 | 0.0042 | 4.52E-08 | 0.0027 | 30.51 |
| rs11657979 | A | G | 0.245 | -0.0091 | 0.0017 | 4.57E-08 | 0.0025 | 28.65 |
| rs7672622 | A | G | 0.746 | -0.0088 | 0.0016 | 4.72E-08 | 0.0027 | 30.25 |
| rs72672052 | A | T | 0.175 | -0.0101 | 0.0018 | 4.87E-08 | 0.0028 | 31.48 |
| rs73055556 | A | G | 0.139 | 0.0111 | 0.002 | 4.95E-08 | 0.0027 | 30.80 |
| rs2055940 | A | G | 0.322 | 0.0082 | 0.0015 | 4.95E-08 | 0.0026 | 29.88 |

**Supplementary table 10.** SNPs associated with average total household income before tax at the genome-wide level of significance (*P* < 5 x 10^-8^) and clumped for independence at kb = 10,000 and *r^2^* = 0.01

| **SNP** | **EA** | **OA** | **EAF** | **BETA** | **SE** | ***P*-value** | **R^2^ %** | **F** |
| --- | --- | --- | --- | --- | --- | --- | --- | --- |
| rs10429582 | T | C | 0.584 | -0.027 | 0.0027 | 7.60E-24 | 0.0255 | 101.39 |
| rs10761035 | G | A | 0.812 | -0.019 | 0.0034 | 3.70E-08 | 0.0076 | 30.32 |
| rs11191116 | C | T | 0.648 | 0.016 | 0.0028 | 3.50E-09 | 0.0088 | 34.89 |
| rs11588857 | G | A | 0.791 | -0.021 | 0.0032 | 4.90E-11 | 0.0109 | 43.22 |
| rs11665242 | A | G | 0.577 | 0.018 | 0.0027 | 6.00E-11 | 0.0108 | 42.82 |
| rs11678501 | T | C | 0.975 | 0.052 | 0.0085 | 1.20E-09 | 0.0093 | 36.98 |
| rs11714337 | G | A | 0.569 | -0.015 | 0.0027 | 9.40E-09 | 0.0083 | 32.97 |
| rs11877758 | T | G | 0.688 | 0.020 | 0.0029 | 1.20E-12 | 0.0127 | 50.43 |
| rs11917431 | C | T | 0.699 | -0.023 | 0.0029 | 3.10E-15 | 0.0156 | 62.19 |
| rs1229984 | T | C | 0.027 | 0.049 | 0.0080 | 1.00E-09 | 0.0094 | 37.23 |
| rs12477364 | G | A | 0.786 | -0.019 | 0.0032 | 1.80E-09 | 0.0091 | 36.17 |
| rs12531825 | G | A | 0.877 | 0.026 | 0.0041 | 2.10E-10 | 0.0102 | 40.40 |
| rs12692596 | C | T | 0.628 | 0.015 | 0.0027 | 1.70E-08 | 0.0080 | 31.78 |
| rs12883788 | C | T | 0.540 | 0.019 | 0.0027 | 1.40E-12 | 0.0126 | 50.14 |
| rs13002946 | T | A | 0.731 | -0.021 | 0.0030 | 7.30E-12 | 0.0118 | 46.94 |
| rs1421334 | A | C | 0.451 | -0.016 | 0.0027 | 1.00E-09 | 0.0094 | 37.24 |
| rs1455350 | T | A | 0.523 | 0.017 | 0.0027 | 9.20E-11 | 0.0106 | 41.99 |
| rs2068428 | C | T | 0.761 | -0.017 | 0.0031 | 4.20E-08 | 0.0076 | 30.07 |
| rs2332719 | A | G | 0.721 | 0.018 | 0.0030 | 5.70E-10 | 0.0097 | 38.42 |
| rs2362523 | A | G | 0.660 | -0.016 | 0.0028 | 1.40E-08 | 0.0081 | 32.15 |
| rs2422859 | T | G | 0.528 | -0.016 | 0.0026 | 6.20E-10 | 0.0096 | 38.25 |
| rs2515919 | A | G | 0.630 | 0.016 | 0.0027 | 5.70E-09 | 0.0085 | 33.95 |
| rs2820314 | A | C | 0.662 | 0.017 | 0.0028 | 2.90E-09 | 0.0089 | 35.27 |
| rs3130264 | C | G | 0.486 | 0.015 | 0.0026 | 5.10E-09 | 0.0086 | 34.16 |
| rs32940 | T | C | 0.299 | -0.021 | 0.0029 | 2.90E-13 | 0.0134 | 53.28 |
| rs34473884 | G | A | 0.751 | -0.017 | 0.0031 | 1.70E-08 | 0.0080 | 31.77 |
| rs34811474 | G | A | 0.768 | -0.018 | 0.0031 | 1.40E-08 | 0.0081 | 32.17 |
| rs387780 | T | C | 0.326 | -0.017 | 0.0028 | 3.30E-09 | 0.0088 | 35.01 |
| rs4115668 | G | A | 0.653 | 0.018 | 0.0028 | 1.10E-10 | 0.0104 | 41.56 |
| rs4673904 | A | T | 0.609 | 0.016 | 0.0027 | 3.30E-09 | 0.0088 | 35.02 |
| rs488786 | C | T | 0.836 | -0.021 | 0.0036 | 6.70E-09 | 0.0085 | 33.63 |
| rs55680124 | C | T | 0.845 | 0.021 | 0.0037 | 4.40E-09 | 0.0087 | 34.45 |
| rs55938136 | A | G | 0.776 | 0.022 | 0.0032 | 1.60E-12 | 0.0126 | 49.96 |
| rs5754738 | A | G | 0.290 | 0.016 | 0.0029 | 2.70E-08 | 0.0078 | 30.95 |
| rs589914 | A | G | 0.315 | -0.016 | 0.0029 | 4.40E-08 | 0.0075 | 29.98 |
| rs6035877 | A | C | 0.531 | 0.015 | 0.0027 | 4.30E-08 | 0.0075 | 29.99 |
| rs62183028 | G | T | 0.689 | 0.019 | 0.0029 | 3.20E-11 | 0.0111 | 44.02 |
| rs6429636 | G | T | 0.280 | -0.019 | 0.0029 | 5.00E-11 | 0.0109 | 43.17 |
| rs6690195 | C | T | 0.513 | 0.016 | 0.0027 | 1.70E-09 | 0.0091 | 36.34 |
| rs6699397 | A | G | 0.630 | 0.019 | 0.0027 | 3.50E-12 | 0.0122 | 48.41 |
| rs6868457 | T | C | 0.522 | -0.021 | 0.0027 | 3.10E-15 | 0.0156 | 62.20 |
| rs71576284 | C | A | 0.991 | 0.084 | 0.0152 | 3.30E-08 | 0.0077 | 30.55 |
| rs73015322 | G | T | 0.923 | 0.027 | 0.0050 | 4.10E-08 | 0.0076 | 30.12 |
| rs75413320 | T | C | 0.892 | 0.026 | 0.0043 | 9.50E-10 | 0.0094 | 37.43 |
| rs7700107 | A | C | 0.862 | 0.023 | 0.0038 | 2.50E-09 | 0.0089 | 35.53 |
| rs77126132 | G | A | 0.907 | -0.027 | 0.0046 | 5.20E-09 | 0.0086 | 34.12 |
| rs784256 | G | A | 0.189 | 0.025 | 0.0034 | 7.90E-14 | 0.0140 | 55.83 |
| rs7896518 | A | G | 0.572 | -0.015 | 0.0027 | 4.10E-08 | 0.0076 | 30.09 |
| rs891953 | C | G | 0.408 | 0.015 | 0.0027 | 4.50E-08 | 0.0075 | 29.91 |
| rs9388490 | C | T | 0.560 | -0.015 | 0.0027 | 1.50E-08 | 0.0081 | 32.04 |
| rs9556958 | C | T | 0.476 | 0.015 | 0.0027 | 8.50E-09 | 0.0083 | 33.16 |
| rs9596313 | G | A | 0.494 | -0.015 | 0.0026 | 7.80E-09 | 0.0084 | 33.34 |
| rs968050 | C | T | 0.517 | -0.022 | 0.0027 | 3.10E-17 | 0.0179 | 71.30 |
| rs9891103 | C | T | 0.771 | 0.023 | 0.0031 | 9.40E-14 | 0.0139 | 55.49 |

**Supplementary table 11.** SNPs associated with Townsend deprivation index at recruitment at the genome-wide level of significance (*P* < 5 x 10^-8^) and clumped for independence at kb = 10,000 and *r^2^* = 0.01

| **SNP** | **EA** | **OA** | **EAF** | **BETA** | **SE** | ***P*-value** | **R^2^ %** | **F** |
| --- | --- | --- | --- | --- | --- | --- | --- | --- |
| rs2403326 | A | G | 0.514 | -0.012 | 0.0020 | 1.20E-08 | 0.0070 | 32.45 |
| rs7740440 | G | A | 0.513 | -0.012 | 0.0020 | 5.70E-09 | 0.0073 | 33.93 |
| rs1947083 | G | A | 0.358 | -0.012 | 0.0021 | 1.90E-08 | 0.0068 | 31.61 |
| rs704067 | G | A | 0.546 | -0.012 | 0.0020 | 3.90E-09 | 0.0075 | 34.67 |
| rs253125 | T | C | 0.250 | -0.013 | 0.0023 | 1.90E-08 | 0.0068 | 31.61 |
| rs4785187 | G | A | 0.777 | -0.014 | 0.0024 | 1.50E-08 | 0.0069 | 32.06 |
| rs989532 | A | G | 0.647 | -0.014 | 0.0021 | 7.40E-11 | 0.0092 | 42.41 |
| rs12133063 | C | A | 0.653 | -0.015 | 0.0021 | 6.10E-12 | 0.0102 | 47.30 |
| rs990706 | C | T | 0.829 | -0.016 | 0.0027 | 7.90E-09 | 0.0072 | 33.29 |
| rs113345285 | T | C | 0.863 | -0.016 | 0.0030 | 3.20E-08 | 0.0066 | 30.56 |
| rs12203592 | C | T | 0.780 | -0.017 | 0.0024 | 1.70E-12 | 0.0108 | 49.77 |
| rs56142341 | G | C | 0.938 | 0.026 | 0.0044 | 5.20E-09 | 0.0074 | 34.12 |
| rs1483246 | T | C | 0.445 | 0.012 | 0.0020 | 3.10E-09 | 0.0076 | 35.10 |
| rs78257128 | C | T | 0.846 | 0.017 | 0.0028 | 2.40E-09 | 0.0077 | 35.58 |
| rs6931604 | C | T | 0.399 | 0.012 | 0.0021 | 4.10E-09 | 0.0075 | 34.58 |
| rs62477310 | T | C | 0.513 | 0.012 | 0.0020 | 5.40E-09 | 0.0074 | 34.02 |
| rs3865018 | C | T | 0.734 | 0.013 | 0.0023 | 4.20E-09 | 0.0075 | 34.52 |
| rs11855821 | G | A | 0.717 | 0.013 | 0.0023 | 3.90E-08 | 0.0065 | 30.20 |

**Supplementary table 12.** SNPs associated with CAD in individuals with diabetes at the genome-wide level of significance (*P* < 5x10^-6^) and clumped for independence at kb = 10,000 and r^2^ = 0.01;

| **SNP** | **EA** | **OA** | **EAF** | **BETA** | **SE** | ***P*-value** | **R^2^ %** | **F** |
| --- | --- | --- | --- | --- | --- | --- | --- | --- |
| rs743241 | A | G | 0.503 | -0.126 | 0.0269 | 2.69E-06 | 0.1404 | 22.02 |
| rs75567373 | G | C | 0.104 | 0.193 | 0.0422 | 4.96E-06 | 0.1329 | 20.85 |
| rs150727872 | A | G | 0.022 | -0.506 | 0.1092 | 3.56E-06 | 0.1370 | 21.49 |
| rs646776 | T | C | 0.777 | 0.165 | 0.0327 | 4.82E-07 | 0.1615 | 25.33 |
| rs74617384 | T | A | 0.082 | 0.324 | 0.0465 | 3.19E-12 | 0.3091 | 48.56 |
| rs56921855 | G | A | 0.168 | 0.172 | 0.0348 | 8.46E-07 | 0.1546 | 24.25 |
| rs10811652 | C | A | 0.494 | 0.177 | 0.0270 | 6.04E-11 | 0.2725 | 42.80 |

**Supplementary Table 13.** The complementary MR analyses results of the causal effects of exposures on CAD in diabetic patients.

| **Exposures** | **MR methods** | ***OR* (95% *CI*)** | ***P* -value** |
| --- | --- | --- | --- |
| **Lifestyle factors** |  |  |  |
| Smoking initiation | Weighted Median | 1.27 (0.99-1.63) | 0.065 |
|  | MR-Egger regression | 0.93 (0.44-2.00) | 0.860 |
|  | MR-RAPS | 1.30 (1.08-1.57) | 0.005 |
|  | MR-PRESSO | 1.30 (1.09-1.54) | 0.004 |
| Lifetime smoking index | Weighted Median | 1.66 (0.78-3.54) | 0.186 |
|  | MR-Egger regression | 0.60 (0.07-5.41) | 0.649 |
|  | MR-RAPS | 1.88 (1.13-3.12) | 0.015 |
|  | MR-PRESSO | 1.84 (1.08-3.16) | 0.027 |
| Alcohol drinking | Weighted Median | 1.96 (0.75-5.09) | 0.168 |
|  | MR-Egger regression | 1.20 (0.21-6.79) | 0.838 |
|  | MR-RAPS | 1.33 (0.72-2.44) | 0.362 |
|  | MR-PRESSO | 1.32 (0.71-2.44) | 0.378 |
| Sleep duration | Weighted Median | 0.79 (0.38-1.63) | 0.516 |
|  | MR-Egger regression | 1.71 (0.35-8.42) | 0.513 |
|  | MR-RAPS | 0.72 (0.44-1.18) | 0.192 |
|  | MR-PRESSO | 0.73 (0.42-1.27) | 0.267 |
| Insomnia | Weighted Median | 1.13 (0.98-1.31) | 0.086 |
|  | MR-Egger regression | 0.99 (0.68-1.44) | 0.965 |
|  | MR-RAPS | 1.16 (1.05-1.29) | 0.003 |
|  | MR-PRESSO | 1.16 (1.06-1.27) | 0.002 |
| AMPA | Weighted Median | 0.99 (0.86-1.14) | 0.849 |
|  | MR-Egger regression | 0.93 (0.60-1.45) | 0.763 |
|  | MR-RAPS | 0.99 (0.88-1.12) | 0.868 |
|  | MR-PRESSO | 0.99 (0.93-1.06) | 0.774 |
| Coffee consumption | Weighted Median | 1.00 (0.99-1.01) | 0.843 |
|  | MR-Egger regression | 1.00 (0.98-1.02) | 0.886 |
|  | MR-RAPS | 1.00 (0.99-1.01) | 0.700 |
|  | MR-PRESSO | 1.00 (0.99-1.01) | 0.621 |
| **Socioeconomic status** |  |  |  |
| Educational attainment | Weighted Median | 0.71 (0.49-1.03) | 0.070 |
|  | MR-Egger regression | 0.53 (0.22-1.31) | 0.171 |
|  | MR-RAPS | 0.60 (0.47-0.78) | <0.001 |
|  | MR-PRESSO | 0.61 (0.47-0.78) | <0.001 |
| Average total household income before tax | Weighted Median | 0.74 (0.39-1.41) | 0.360 |
|  | MR-Egger regression | 1.13 (0.15-8.59) | 0.904 |
|  | MR-RAPS | 0.63 (0.40-1.00) | 0.048 |
|  | MR-PRESSO | 0.64 (0.41-0.98) | 0.047 |
| Townsend deprivation index | Weighted Median | 1.48 (0.35-6.17) | 0.593 |
|  | MR-Egger regression | 228.02 (0.45-1.16E+5) | 0.108 |
|  | MR-RAPS | 1.79 (0.61-5.27) | 0.293 |
|  | MR-PRESSO | 1.76 (0.64-4.85) | 0.291 |

**Supplementary table 14.** The complementary MR analyses results of the causal effects of CAD in diabetic patients on outcomes.

| **Outcomes** | **MR methods** | ***OR* (95% *CI*)** | ***P* -value** |
| --- | --- | --- | --- |
| **Lifestyle factors** |  |  |  |
| Smoking initiation | Weighted Median | 1.002 (0.989-1.015) | 0.791 |
|  | MR-Egger regression | 1.004 (0.967-1.044) | 0.833 |
|  | MR-RAPS | 1.001 (0.992-1.011) | 0.825 |
|  | MR-PRESSO | 1.001 (0.989-1.013) | 0.869 |
| Lifetime smoking index | Weighted Median | 0.991 (0.982-1.000) | 0.048 |
|  | MR-Egger regression | 0.998 (0.978-1.019) | 0.885 |
|  | MR-RAPS | 0.991 (0.984-0.999) | 0.021 |
|  | MR-PRESSO | 0.991 (0.987-0.996) | 0.010 |
| Alcohol drinking | Weighted Median | 1.009 (0.996-1.023) | 0.179 |
|  | MR-Egger regression | 1.023 (0.982-1.066) | 0.327 |
|  | MR-RAPS | 1.005 (0.994-1.015) | 0.366 |
|  | MR-PRESSO | 1.005 (0.991-1.018) | 0.537 |
| Sleep duration | Weighted Median | 1.000 (0.984-1.016) | 0.983 |
|  | MR-Egger regression | 0.999 (0.963-1.037) | 0.975 |
|  | MR-RAPS | 1.001 (0.988-1.014) | 0.908 |
|  | MR-PRESSO | 1.001 (0.998-1.005) | 0.710 |
| Insomnia | Weighted Median | 0.998 (0.968-1.030) | 0.925 |
|  | MR-Egger regression | 0.996 (0.922-1.077) | 0.932 |
|  | MR-RAPS | 0.999 (0.972-1.027) | 0.963 |
|  | MR-PRESSO | 0.999 (0.984-1.015) | 0.937 |
| AMPA | Weighted Median | 1.173 (0.931-1.478) | 0.177 |
|  | MR-Egger regression | 0.820 (0.465-1.446) | 0.523 |
|  | MR-RAPS | 1.067 (0.880-1.294) | 0.510 |
|  | MR-PRESSO | 1.065 (0.877-1.291) | 0.549 |
| Coffee consumption | Weighted Median | 1.003 (0.993-1.013) | 0.544 |
|  | MR-Egger regression | 0.995 (0.972-1.018) | 0.697 |
|  | MR-RAPS | 0.999 (0.991-1.008) | 0.872 |
|  | MR-PRESSO | 0.999 (0.992-1.006) | 0.857 |
| **Socioeconomic status** |  |  |  |
| Educational attainment | Weighted Median | 0.997 (0.986-1.009) | 0.662 |
|  | MR-Egger regression | 0.998 (0.969-1.025) | 0.831 |
|  | MR-RAPS | 1.000 (0.991-1.009) | 0.974 |
|  | MR-PRESSO | 1.000 (0.991-1.009) | 0.974 |
| Average total household income before tax | Weighted Median | 0.973 (0.956-0.991) | 0.003 |
|  | MR-Egger regression | 0.971 (0.932-1.011) | 0.207 |
|  | MR-RAPS | 0.979 (0.965-0.993) | 0.003 |
|  | MR-PRESSO | 0.979 (0.967-0.992) | 0.018 |
| Townsend deprivation index | Weighted Median | 0.996 (0.981-1.011) | 0.613 |
|  | MR-Egger regression | 1.004 (0.961-1.049) | 0.858 |
|  | MR-RAPS | 0.991 (0.981-1.002) | 0.116 |
|  | MR-PRESSO | 0.992 (0.978-1.006) | 0.305 |
